# Tracking the Amide I and αCOO− Terminal ν(C=O) Raman Bands in a Family of l-Glutamic Acid-Containing Peptide Fragments: A Raman and DFT Study

**DOI:** 10.3390/molecules26164790

**Published:** 2021-08-07

**Authors:** Ashley E. Williams, Nathan I. Hammer, Ryan C. Fortenberry, Dana N. Reinemann

**Affiliations:** 1 Department of Chemistry and Biochemistry, University of Mississippi, University, MS 38677, USA; aewilli5@go.olemiss.edu (A.E.W.); nhammer@olemiss.edu (N.I.H.); 2 Department of Biomedical Engineering, University of Mississippi, University, MS 38677, USA; 3 Department of Chemical Engineering, University of Mississippi, University, MS 38677, USA

**Keywords:** peptide, E-hook, Raman, DFT, vibrational spectroscopy

## Abstract

The E-hook of β-tubulin plays instrumental roles in cytoskeletal regulation and function. The last six C-terminal residues of the βII isotype, a peptide of amino acid sequence EGEDEA, extend from the microtubule surface and have eluded characterization with classic X-ray crystallographic techniques. The band position of the characteristic amide I vibration of small peptide fragments is heavily dependent on the length of the peptide chain, the extent of intramolecular hydrogen bonding, and the overall polarity of the fragment. The dependence of the E residue’s amide I ν(C=O) and the αCOO− terminal ν(C=O) bands on the neighboring side chain, the length of the peptide fragment, and the extent of intramolecular hydrogen bonding in the structure are investigated here via the EGEDEA peptide. The hexapeptide is broken down into fragments increasing in size from dipeptides to hexapeptides, including EG, ED, EA, EGE, EDE, DEA, EGED, EDEA, EGEDE, GEDEA, and, finally, EGEDEA, which are investigated with experimental Raman spectroscopy and density functional theory (DFT) computations to model the zwitterionic crystalline solids (in vacuo). The molecular geometries and Boltzmann sum of the simulated Raman spectra for a set of energetic minima corresponding to each peptide fragment are computed with full geometry optimizations and corresponding harmonic vibrational frequency computations at the B3LYP/6-311++G(2df,2pd) level of theory. In absence of the crystal structure, geometry sampling is performed to approximate solid phase behavior. Natural bond order (NBO) analyses are performed on each energetic minimum to quantify the magnitude of the intramolecular hydrogen bonds. The extent of the intramolecular charge transfer is dependent on the overall polarity of the fragment considered, with larger and more polar fragments exhibiting the greatest extent of intramolecular charge transfer. A steady blue shift arises when considering the amide I band position moving linearly from ED to EDE to EDEA to GEDEA and, finally, to EGEDEA. However, little variation is observed in the αCOO− ν(C=O) band position in this family of fragments.

## 1. Introduction

The amide I vibration has been found to be heavily dependent on the formation of intramolecular hydrogen bonds in proteins, and hydrogen bonds, in turn, are integral in the stabilization of various protein secondary structures [1,2,3,4,5,6,7,8,9]. As a result, vibrational spectroscopy combined with density functional theory (DFT) computations is commonly employed to investigate the molecular geometries, dynamics, and secondary structures of short peptide chains [9,10,11,12,13,14,15,16,17,18,19,20,21,22,23,24,25,26,27,28,29,30,31,32,33,34,35,36,37,38,39,40,41,42,43]. Navarette and coworkers reported the Raman and IR vibrational spectra for l-glutamic acid [44], l-aspartic acid (D) [35], and the glutamic acid (EE) and aspartic acid (DD) dipeptides [35] in their seminal works and found that these molecules are zwitterionic in the crystalline state, confirmed with both X-ray and neutron diffraction techniques. The Raman spectra of the zwitterionic dipeptide, l-aspartyl-l-glutamic acid (DE), in solid and solution have also been reported compared to simulated spectra from DFT computations at the B3LYP/cc-pVDZ level of theory [41]. Kauser et al. concluded that the zwitterionic form of DE in the solid state is stabilized by strong inter- and intra-molecular hydrogen bonds and, furthermore, characterize the amide I band position of the DE dipeptide at 1674 cm^−1^ in solid state Raman spectra [41]. In one study, the vibrational band positions of GxG (x = D, aspartic acid; N, asparagine; or C, cysteine) and various other tripeptides were analyzed with Raman spectroscopy, circular dichroism, and DFT computations [11,21,45,46]. These fully protonated tripeptides were found to have an above-average propensity for conformations, which are usually found in turn regions of peptide chains. Rybka and coworkers concluded that these tripeptides, containing the D, N, or C amino acids at the core, might facilitate the formation of hairpin-like regions in the unfolded state of proteins and could potentially support the initiation of protein folding processes [11].

Much less work has been carried out in the application of quantum chemical techniques to investigate larger peptide systems due to the computational cost required for such analyses. The conformational complexity of polypeptides has thus led to the development of various methods to provide accurate structures and frequencies of large systems while minimizing the computational cost. Bou$\overset{ˇ}{r}$ and Keiderling introduced the Cartesian transfer method, which applies a direct transfer of Cartesian molecular force fields (FF) and electric property tensors as opposed to the traditionally employed internal coordinates [44]. They included atomic polar and axial tensors in the transfer for computation of the vibrational frequencies. They investigated N-methylacetamide, a tripeptide, and a helical heptapeptide and found that their Cartesian transfer method performed well in the systems selected but has many limitations, most importantly that it cannot be used for aromatic systems with conjugated π-bond systems. Interestingly, they assembled the force fields for larger molecules from smaller fragments to decrease the computational cost even further [44]. Bou$\overset{ˇ}{r}$ and Keiderling applied their Cartesian transfer method to optimize five standard helical structures (α, 3_10_-,3_1_-, and left handed) at the B3LYP/SV(P) level of theory, while simulating the solvent effects with COSMO (conductor-like screening solvent model) [28]. They computed the vibrational frequencies with the BPW91/6-31G* level of theory. Use of the polarized continuum stabilizes the hydrogen bonds in each system under study, and their computed structural parameters agree well with previously reported X-ray structures for native-state proteins [28]. Bou$\overset{ˇ}{r}$ and Keiderling also developed a normal mode-based method for quantum chemical optimization of molecular geometries [45], which was found to provide smooth convergence for each of the systems under study. Although their method cannot be used if the exact valence coordinates are desired, it provides a useful complementary tool when employing traditional internal valence coordinate-based optimizations.

Reiher introduced his mode-tracking algorithm in 2007, which selectively calculates specific areas of vibrations instead of computing all of the normal modes and frequencies at once, called localized normal modes [46,47]. In his model polypeptide, (Ala)_20_, Reiher demonstrates that the localized modes represent the displacements of only a few atoms at a time and are obtained by first optimizing the structure with a DFT method and then performing vibrational frequency computations to find the sole transformation of the normal modes within one band of the spectrum whose accuracy was derived by previously established localization criterion [46]. The localized modes method proved to be more accurate at computing the band positions of the normal modes than those previously described, including its use in computing the coupling constants that arise when the modes are delocalized throughout the structure. Their use of the highly structured (Ala)_20_ peptide made arriving at an optimized geometry relatively simple, though, and they only considered a single conformation, without further investigation of the conformational space. Reiher and coworkers have applied this method to investigate the Raman optical activity (ROA) signatures of four structurally similar peptides with a common backbone conformation but varying sequences of amino acid configurations at the BP86/TZVP level of theory [48]. They found that the amino acid configuration plays a significant role on the ROA peaks in the amide I, II, and III regions. Additionally, Reiher applied the localized mode methodology to investigate secondary structure effects on the IR and Raman spectra of the (Ala)_20_ polypeptide in the α-helical or 3_10_-helical conformation [49].

Hydrogen bonding has been found to play an important role in protein stability both in the folded and unfolded states [2,4,5]. Several studies have investigated hydrogen bonding patterns in peptide chains and the effects of intramolecular hydrogen bonding and other noncovalent interactions on the position of the amide I band [2,3,4,5,6,8,9,15,32,50,51,52,53,54,55,56,57,58]. A study by Görbitz et al. investigated the hydrogen bonding interactions in the crystal structures of unprotected, zwitterionic dipeptides and found that hydrogen-bonding patterns arise in which the dipeptides orient themselves in head-to-tail chains involving the N-terminal amino and C-terminal carboxylate groups [51]. Kumar and coworkers recently investigated the folded structures of Z-Gly-Pro-OH dipeptides (Z = benzyloxycarbonyl) with gas phase spectroscopy and DFT computations and observed a weak intramolecular hydrogen bond in the experimental spectrum that corresponds to the backbone amide N-H and backbone carbonyl C=O hydrogen bond (termed a C5 hydrogen bond). Their natural bond order (NBO) analysis provides evidence of delocalization of the electrons in the *p*-type lone-pair orbital of the carbonyl oxygen atom to the σ* orbital of the N-H group [50]. The hydrogen-bonding patterns formed by these structures can also provide evidence for the secondary structure of the protein of interest.

L-glutamic acid (E) is a polar amino acid, and the reactivity of E’s side chain results in the facilitation and regulation of many biochemical reactions. For example, the E-hook of β-tubulin is instrumental in cytoskeletal regulation and function. The last six C-terminal residues of the βII isotype, amino acid sequence EGEDEA, protrude from the microtubule surface and facilitate protein binding and molecular motor motility [59,60,61,62,63,64,65,66,67,68,69,70,71,72,73,74,75,76,77,78,79,80,81,82,83,84]. Unlike investigations by Bour and Reiher, which investigate ordered peptide chains falling into the classic α-helical and β-sheet secondary structure domains [28,44,45,46,48,49,85,86,87], EGEDEA is thought to be an intrinsically disordered protein, in part due to the inability to resolve a crystal structure. Knowing that many peptide conformers are possible, an approach using experimental Raman and simulated DFT characterization together facilitates the approximation of the solid phase behavior of the molecule in vacuo. A recent publication by our group characterizes the vibrational band positions of the E-hook hexapeptide, EGEDEA, and the peptide fragments used to build it computationally, EG, ED, EA, EGED, and EDEA, using experimental Raman spectroscopy combined with DFT computations [10]. Since there is limited conformational freedom due to the partial rigidity of the peptide backbone, small variations in the molecular geometry are not represented in the Raman spectrum. The remarkable similarity observed in the experimental Raman spectra as the size of the peptide fragment increases along with the observed dependence of the amide I vibration on the overall amino acid composition of the fragment warrants further investigation into the intramolecular interactions occurring in these fragments and how they affect the vibrational band positions. In this study, the EGEDEA hexapeptide is broken down into 11 fragments (EG, ED, EA, EGE, EDE, DEA, EGED, EDEA, EGEDE, GEDEA, and, finally, EGEDEA) and analyzed with experimental Raman spectroscopy and DFT computations, including natural bond order analyses (NBO), to track the amide I vibration and the extent of intramolecular charge transfer as the size of the fragment increases and with variation of the neighboring residue.

## 2. Methods

### 2.1. Experimental Methods

The β-TUBB2A E-hook in the form of a synthetic peptide of charged amino acid sequence EGEDEA^4−^ is acquired in crystalline form from Genscript at >95% purity. Additional peptide fragments EG^1−^, ED^2−^, EA^1−^, EGED^3−^, and EDEA^3−^ are also acquired in the same manner and purity. The samples are stored at −20 °C to ensure that they remained in their charged state for Raman analysis. A Horiba Scientific LabRAM HR Evolution Raman Spectroscopy system with CCD camera detection is used to analyze the crystalline solids of the peptide fragments. A 532 nm laser line is used as the excitation source, and either a 600 or 1800 grooves/mm grating is used for detection, affording a resolution of less than 1 cm^−1^. Additionally, Raman studies at −100 °C utilize a temperature-controlled stage allowing the formation of crystals via a controlled flow of liquid nitrogen over the sample. The vibrational characterization of only the EGEDEA hexapeptide is performed with the temperature-controlled data acquired.

### 2.2. Theoretical Methods

Full geometry optimizations and harmonic vibrational frequency computations are employed to identify a set of energetic minima for each of the peptide fragments of interest and the corresponding vibrational frequencies and Raman activities of the normal modes. Each fragment is optimized in a step-wise fashion at the B3LYP/3-21G, 6-31G, 6-31+G(*d*,*p*), and, finally, 6-311++G(2*df*,2*pd*) levels of theory [88,89,90]; the last of which the vibrational frequencies are also computed. The B3LYP/6-311+G(2*df*,2*pd*) level of theory has been used in the literature to describe smaller biological peptides [42,43,91,92]. Additional methods are employed to investigate the EG dipeptide, all with the 6-311++G(2*df*,2*pd*) basis set, including M06-2X [93], PBEPBE [94], and MP2 [91], to determine the validity of B3LYP/6-311++G(2*df*,2*pd*). Natural bond order (NBO) computations are performed at the B3LYP/6-311++G(2*df*,2*pd*) level of theory on every structure identified to investigate the effects of intramolecular hydrogen bonding [92]. All computations employ the Gaussian16 computational chemistry program [95].

In lieu of molecular dynamics simulations to locate all of the possible local minima for each fragment, a Maxwell–Boltzmann statistical distribution of energetic minima for each is collected by first isolating a single energetic minimum and then varying the peptide bond dihedral angles to search for additional low-energy conformations that fall within an appropriate range. From this set of candidate structures, a Boltzmann sum of the vibrational frequencies and intramolecular hydrogen bonds are employed to describe the experimental band positions of the amide I and αCOO− terminal ν(C=O) vibrational modes. Maxwell-Boltzmann statistics give the distribution of microstates in a system (termed the macrostate) as a function of temperature, defining the probability that a molecule will exist in a certain “state” of given energy at a certain temperature. This method assumes that an array of particles, N, has a total energy and that the energies of the individual particles take on discrete values, *E*_0_, *E*_1_, …, *E_i_*. The number of particles with energy *E*_0_ is *N*_0_, with *E*_1_ is *N*_1_, etc. The word ”macrostate” is now applied to describe the gross state that corresponds to a given set of numerical values, *N_1_*, *N*_2_, …, *N_i_* [96]. The logarithm of the fraction of particles in a given microstate is proportional to the ratio of the energy of that state to the temperature of the system:

(1) $$-\log\left(\frac{N_{i}}{N}\right)\propto \frac{E_{i}}{T}$$

Maxwell–Boltzmann statistical thermodynamics assumes the molecules are independent from one another. Additionally, the states are considered to be in thermal equilibrium. Knowing Boltzmann’s factor of e^−*Ei*/*kT*^, the above equation can be rearranged and represents the absolute probability for the occurrence of state *i*:

(2) $$N_{i}=\frac{e^{\left(-\frac{E_{i}}{kT}\right)}}{\Sigma_{j}e^{\left(-\frac{E_{j}}{kT}\right)}}$$

where *N_i_* is the expected number of particles in the single-particle microstate, *i*; *N* is the total number of particles in the system; *E_i_* is the energy of the microstate, *i*; *T* is the equilibrium temperature of the system; and *k* is the Boltzmann constant at 1.38 × 10^−23^ J/K [96,97].

Simulated Raman spectra are created by summing Lorentzian profiles for each calculated normal mode and weighting with the corresponding Raman activity. A scaling factor is employed in order to partially account for the anharmonicity of the computed harmonic vibrations. The overestimation of calculated vibrational frequencies is fairly uniform allowing for a single scaling factor to be used for the methods and basis sets employed here. A scaling factor of 0.97 is used here for all levels of theory as has been previously reported to correct for the disagreement of the vibrational frequencies acquired using B3LYP/6-311+G(2*df*,2*pd*) when compared to experiment [98]. The Boltzmann sum of the simulated spectra, *B_T_*, is created by weighting the simulated spectra, W, with the corresponding *N_i_* values of the microstate and then summing over the total available states.

(3) $$B_{T}=\sum^{\;}(N_{1}{*W}_{1})+\left(N_{2}{*W}_{2}\right)+\ldots \left(N_{i}{*W}_{i}\right)$$

**Figure 1:**
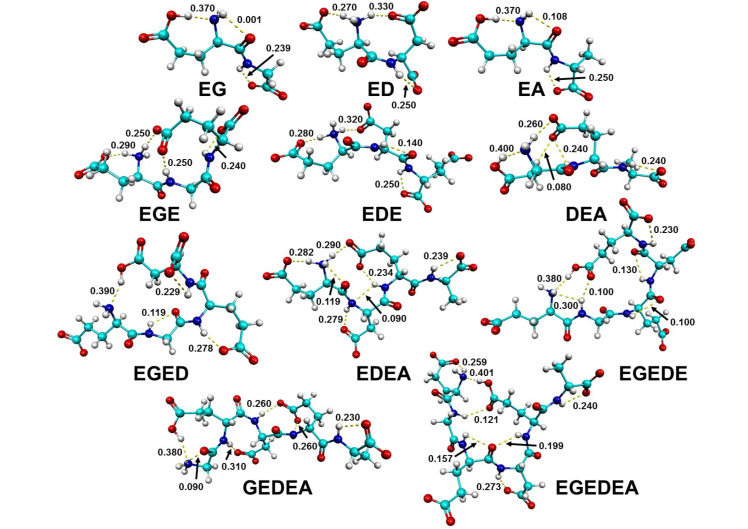
Lowest energy molecular geometries of the EG, ED, EA, EGE, EDE, DEA, EGED, EDEA, EGEDE, GEDEA, and EGEDEA peptide fragments, computed at the B3LYP/6-311++G(2*df*,2*pd*) level of theory. Intramolecular hydrogen bonds are represented by dashed yellow lines and the magnitudes are displayed for each, computed from natural bond order computations, presented in transferred electrons, e^−^.

Sixty-nine geometries are found, with six total energetic minima found corresponding to the ED (Figure S2), EA (Figure S3), EGE (Figure S4), EDE (Figure S5), DEA (Figure S6), EDEA (Figure S8), EGEDE (Figure S9), and GEDEA (Figure S10) peptide fragments and seven total energetic minima found corresponding to the EG (Figure S1), EGED (Figure S7), and EGEDEA (Figure S11) peptide fragments. The lowest energy molecular geometries for each fragment of interest are presented in Figure 1, along with the computed Boltzmann-weighted magnitudes of the intramolecular hydrogen bonds, in millielectrons, e^−^. Arrival at a set of energetic minima for each fragment is achieved by manipulating the φ and ψ bond angles and, as mentioned before, performing the step-wise optimizations beginning first at the B3LYP/3-21G level of theory and building up to 6-31G, 6-31+G(*d*,*p*) and finally 6-311++G(2*df*,2*pd*). B3LYP/6-311++G(2*df*,2*pd*) has been shown to perform well in the literature in description of the experimental Raman spectra of small biomolecules and peptide fragments [40,41,42,99,100,101,102,103]. Regardless, additional methods are employed to compute the lowest energy molecular geometry of the EG dipeptide, shown in Figure S12, to investigate the accuracy of B3LYP/6-311++G(2*df*,2*pd*). Very little variation is observed when comparing the simulated spectra computed with the 6-311++G(2*df*,2*pd*) basis set and the B3LYP, M06-2X, PBEPBE, and MP2 methods. Thus, due to the good performance of the B3LYP/6-311++G(*2df*,*2pd*) level of theory when compared to experiments in peptide studies, only these results are presented and discussed herein. The Boltzmann distribution of the energetic minima for each peptide fragment is computed, employing Equation (2), giving the probability that a molecule will populate a certain state based on the distribution of energies in the set of states considered, as a function of temperature. Since the experimental Raman spectra are acquired under standard room temperature conditions, 298 K is employed in all cases in the Boltzmann equations for the theoretical data. Table 1 presents the relative energies of the six lowest energetic minima for each fragment, along with the computed Boltzmann probabilities (*N_i_*), the number of intramolecular hydrogen bonds formed (HBs), Boltzmann-weighted magnitudes of the intramolecular hydrogen bonds, the Boltzmann-weighted total charge transferred via intramolecular hydrogen bonding (qT), and the computed and scaled harmonic vibrational frequencies of the amide I and αCOO− terminal ν(C=O) bands. The raw data for each fragment can be found in the supporting information (Tables S1–S11). The intramolecular hydrogen bonds are computed from natural bond order analyses by calculating the difference between the computed electron densities of the two participating atoms. The relative energies for each set of energetic minima range from 0 to 50 kcal mol^−1^ to ensure the Boltzmann distribution is fully representative. All computations begin with the fragment in a charged state, including αNH_3_^+^, αCOO−, and all side chains in their zwitterionic states at pH 7, as is the composition of the crystalline solids analyzed experimentally. Interestingly, the molecules consistently tend to protonate themselves in isolation. Upon visualizing several of these protonated bands in the experimental spectra, these molecular geometries (found in the Supporting Information) are included in the Boltzmann distribution of the simulated Raman vibrational spectra. In many cases, protonation is exhibited in the lowest energy conformations (EG, EA, DEA, EGED, EGEDE, GEDEA, and EGEDEA, Figure 1).

## 3. Results and Discussion

### 3.1. Summed Simulated Spectra to Experiment

Many previous investigations have searched for the global minimum of peptides and peptide fragments in order to describe acquired experimental data [104,105,106,107,108,109,110,111,112]. There is no need to search for the actual minimum to describe the experimental band positions because, in the case of these short peptides, the partial rigidity of the peptide bond introduces some extent of structural rigidity, preventing vibrations from deviating tremendously from structure to structure, as is represented in the comparison of the simulated spectra for the various conformations of each peptide fragment in vacuo (Figures S13–S23). A recent publication by our group characterizes the experimental Raman vibrational band positions of the crystalline solid form of the EGEDEA hexapeptide with DFT computations. This is achieved by breaking the hexapeptide down into components (EG, ED, EA, EGED, and EDEA), which are then used to build the EGEDEA hexapeptide computationally [10]. In the previous work, a single energetic minimum for each fragment was acquired for description of the experimental spectra of the crystalline solids. The agreement between experiment and theory is good, but with hopes to more accurately characterize the amide I region and investigate the effects of intramolecular hydrogen bonding on the position of the vibrational band positions, a more robust computational investigation is presented here. All experimental data referenced here can be found in [10] and the corresponding supplementary material.

**Figure 2:**
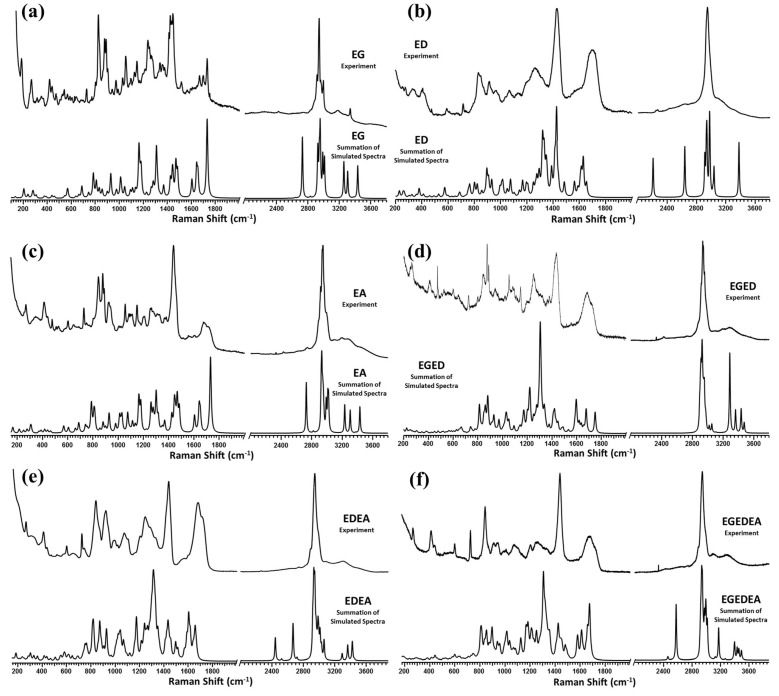
Comparison of the experimental Raman spectrum to the Boltzmann-summed simulated Raman spectrum for the (**a**) EG, (**b**) ED, (**c**) EA, (**d**) EGED, (**e**) EDEA, and (**f**) EGEDEA peptide fragments, presented in wavenumbers, cm^−1^.

To track the amide I and αCOO− terminal ν(C=O) band positions as the size of the fragment increases linearly, the tripeptides EGE, EDE, and DEA and the pentapeptides EGEDE and GEDEA are added to provide a complete picture, and, thus, a theoretical investigation of these fragments is included in this investigation. Figure 2 presents the comparison of the Boltzmann-summed simulated Raman spectra (in vacuo) to the experimental Raman spectra acquired for the EG, ED, EA, EGED, EDEA, and EGEDEA solid peptide fragments. No experimental data corresponding to the tripeptides or pentapeptides are acquired, as the stepwise pathway does not indicate that such would be beneficial.

In all cases, the agreement between experiment and theory improves, supporting the use of a Boltzmann distribution of the energetic minima to describe the vibrational frequencies of short peptide systems when other methods are unfeasible. The experimental Raman spectra are all remarkably similar, reminiscent of the presence of the strongly polar E residue dominating the signal in each case. The variations arise from the composition of the neighboring side chains and length of the peptide chain, which can be investigated by identifying specific vibrational bands that are shared by all peptides and give important information regarding protein secondary structure.

### 3.2. Tracking the Amide I and αCOO− Terminal ν(C=O) Bands

The amide I band in the analyzed peptides appears in the range of 1600–1700 cm^−1^ and consists mainly of the backbone C=O stretching vibration, with minor contributions from the out-of-phase CN stretching vibration, the CCN deformation, and the NH in-plane bend [54]. Table 2 presents the Boltzmann sums of the total charge transferred via intramolecular hydrogen bonding (q_B_T) along with the Boltzmann sums of the amide I and αCOO− terminal ν(C=O) band positions for each peptide fragment of interest compared to the experimental value. An amide I ν(C=O) band is associated with each of the peptide bonds in a protein.

Therefore, throughout this study, the amide I ν(C=O) band is in reference to the first peptide bond from the αNH_3_^+^ terminal end, physically furthest from the αCOO− terminal. These two vibrations are chosen because the atoms involved consistently participate in intramolecular hydrogen bonding, and the band position of the amide I vibration can provide valuable information regarding the secondary structure of the peptide under study. The *y*-axis in Raman spectra is the intensity of the scattered light, related to the number of photons the detector records at each Raman shift. The units are arbitrary and are thus not presented. As shown in Table 2, there is good agreement between the Boltzmann-summed vibrations and those experimentally obtained, all of which have been scaled by 0.97 to partially correct for the anharmonicity of the computed harmonic vibrations.

#### 3.2.1. Amide I and αCOO− Vibrations Depend on Peptide Chain Length and Side-Chain Polarity

**Figure 3:**
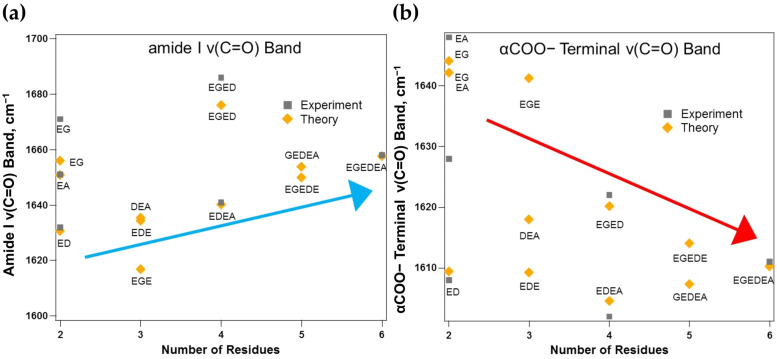
Correlation of the experimental Raman and Boltzmann-summed simulated band positions of the (**a**) amide I ν(C=O) vibration and (**b**) αCOO− terminal ν(C=O) vibration as the length, *n*, of the peptide fragment increases from *n* = 2 to *n* = 6 amino acid residues. All vibrations are presented in wavenumbers, cm^−1^, and simulated spectra are computed at the B3LYP/6-311++G(2*df*,2*pd*) level of theory. The arrows are meant to serve as a visual guide for the trend and do not represent a linear correlation.

Figure 3a,b correlate the experimental and Boltzmann-summed simulated band positions of the amide I and αCOO− ν(C=O) vibrations with the increasing number of residues in the peptide chain. In the case of the dipeptides, EG, the smallest and most nonpolar fragment, possesses an overall 1- charge, three intramolecular hydrogen bonds, and exhibits the amide I ν(C=O) band at 1656 cm^−1^ (exp: 1671 cm^−1^) and the αCOO− ν(C=O) band at 1644 cm^−1^ (exp: 1648 cm^−1^), roughly 10 cm^−1^ apart. The experiment suggests these bands to be almost 20 cm^−1^ apart, which is among the greatest deviations between experiment and theory found in this study, warranting transition dipole computations to correct for the coupling of these vibrational bands in quantum chemical approximations of the vibrational frequencies. In moving from a neighboring glycine to a neighboring alanine residue, only slightly more polar than EG due to the larger electron-withdrawing effects of alanine’s methyl side chain and still possessing a 1- charge and three intramolecular hydrogen bonds, causes the amide I ν(C=O) band to red shift slightly to 1651 cm^−1^ (exp: 1651 cm^−1^) and the αCOO− ν(C=O) band to red shift by only 2 cm^−1^ to 1642 cm^−1^. The experiment, however, exhibits a 20 cm^−1^ red shift from the EG to EA dipeptide for both the amide I and αCOO− ν(C=O) bands. The ED dipeptide is the most polar and largest of the three dipeptides considered, containing three charged carboxylic acids along with the charged αNH_3_^+^ terminal, an overall 2- charge, and, again, three intramolecular hydrogen bonds. The amide I band appears at 1631 cm^−1^ (exp: 1632 cm^−1^), and the αCOO− ν(C=O) band appears at 1609 cm^−1^ (exp: 1608 cm^−1^). Both are dramatically red shifted when compared to these bands in the EG and EA dipeptides. Due to the partial conjugation of the peptide backbone, electron density can be delocalized throughout, and fragments containing only charged residues exhibit no barriers through which the density must travel, exhibiting an overall stabilizing effect on the energies of participating vibrational bands. In all three dipeptides, the amide I band appears higher in energy than the αCOO− ν(C=O) band.

All three tripeptide fragments exhibit some degree of polarity, with the EGE tripeptide being the least polar due to the separation of the two polar E residues by the nonpolar G residue, and the EDE residue being the most polar due to the presence of three charged side chains. The EGE tripeptide possesses an overall 2- charge and four intramolecular hydrogen bonds. EGE’s amide I band position appears at 1617 cm^−1^, and the αCOO− ν(C=O) band appears at 1641 cm^−1^, which is interesting because the amide I band position is now shifted to appear lower in energy than the αCOO− ν(C=O) band, opposite to that seen in the EG dipeptide. The DEA tripeptide is the second most polar of the three fragments, possessing an overall 2- charge and five intramolecular hydrogen bonds. In DEA’s case, the amide I band is blue shifted from EGE to 1634 cm^−1^, and the αCOO− ν(C=O) band is red shifted to 1618 cm^−1^. Unlike EGE, the amide I band shifts back to higher in energy than the αCOO− ν(C=O) band. DEA’s amide I vibration is blue shifted only 4 cm^−1^ from the predicted position for the ED dipeptide, while this same vibration is red shifted by 16 cm^−1^ from the EA dipeptide. This behavior provides evidence of the dependence of the amide I band on neighboring side-chain polarity. The most polar tripeptide under study, EDE, exhibits an overall 3- charge and four intramolecular hydrogen bonds, with the amide I band appearing very close to that predicted for the DEA tripeptide at 1634 cm^−1^ and the αCOO− ν(C=O) band appearing at 1609 cm^−1^. The amide I band is blue shifted by only 3 cm^−1^ from the predicted position for the ED dipeptide. The αCOO− ν(C=O) band steadily red shifts with increasing side-chain polarity from EGE (1641 cm^−1^) to DEA (1618 cm^−1^) to EDE (1609 cm^−1^).

In the case of the tetrapeptides, EGED and EDEA, EDEA is the most polar due to the presence of three neighboring polar residues. The less polar tetrapeptide, EGED, possesses an overall 3- charge and exhibits four intramolecular hydrogen bonds, with the amide I band appearing at 1676 cm^−1^ (exp: 1686 cm^−1^) and the αCOO− ν(C=O) band appearing at 1620 cm^−1^ (exp: 1622 cm^−1^). Interestingly, unlike the EGE tripeptide’s amide I band position, EGED’s amide I band is red shifted from the EG dipeptide, with no clear trend arising in the transition from EG to EGE to EGED. The αCOO− ν(C=O) band, however, appears to steadily red shift from EG (1644 cm^−1^) to EGE (1641 cm^−1^) and finally to EGED (1620 cm^−1^). The largest red shift in this band is observed upon adding a neighboring polar residue where there was not one before (moving from EGE to EGED). The more polar EDEA residue, which also possesses an overall 3- charge but displays seven intramolecular hydrogen bonds, exhibits the amide I band at 1640 cm^−1^ (exp: 1641 cm^−1^) and the αCOO− ν(C=O) band at 1605 cm^−1^ (exp: 1602 cm^−1^). In this case, a clear trend is arising in the amide I band, with a steady blue shift moving from the ED dipeptide (1631 cm^−1^) to the EDE tripeptide (1634 cm^−1^) to the EDEA tetrapeptide (1640 cm^−1^). In the αCOO− ν(C=O) case, very little change is observed as the length of the peptide chain increases, without varying the overall polarity of the system. The αCOO− ν(C=O) band consistently appears at 1609 cm^−1^ in the ED and EDE peptide fragments, with a slight red shift observed moving up to the EDEA tetrapeptide (1605 cm^−1^).

Two pentapeptides are considered, including EGEDE and GEDEA. The EGEDE pentapeptide is considered the most polar of the two, as it possesses four polar amino acid residues. GEDEA poses a unique case because it is capped by two nonpolar residues, G and A, which could be interesting when considering the observed shifts thus far. GEDEA possesses an overall 3- charge and exhibits six intramolecular hydrogen bonds. GEDEA’s amide I band appears at 1654 cm^−1^, and the αCOO− ν(C=O) band appears at 1607 cm^−1^. The αCOO− ν(C=O) band is blue shifted by only 2 cm^−1^ from EDEA’s αCOO− ν(C=O) band position, expected since only a G residue is added from EDEA to GEDEA. GEDEA fits well into the trend observed in the vibration moving from ED to EDE to EDEA and now to GEDEA. The amide I band also blue shifts by 14 cm^−1^, holding with the trend observed for the amide I band’s steady blue shift moving from ED to EDE to EDEA and now to GEDEA. The more polar EGEDE pentapeptide possesses an overall 4- charge and six intramolecular hydrogen bonds. EGEDE’s amide I band appears at 1650 cm^−1^, while the αCOO− ν(C=O) band appears at 1614 cm^−1^. The addition of a third neighboring side chain to EGED to make EGEDE causes the amide I vibration to dramatically red shift by 26 cm^−1^ and the αCOO− ν(C=O) band to red shift by 6 cm^−1^. EGEDE also holds with the amide I trend observed in the ED/EDE/EDEA/GEDEA case. A clear trend also arises when considering the αCOO− ν(C=O) band position moving from EG (1644 cm^−1^) to EGE (1641 cm^−1^) to EGED (1620 cm^−1^) to EGEDE (1614 cm^−1^).

The final fragment considered in this study, the hexapeptide, EGEDEA, possesses an overall 4- charge and seven intramolecular hydrogen bonds. The amide I band appears at 1658 cm^−1^ (exp: 1658 cm^−1^), and the αCOO− ν(C=O) band appears at 1610 cm^−1^ (exp: 1611 cm^−1^). The amide I band position is slightly blue shifted from both of the pentapeptide band positions, holding with the steady blue shift that arises moving from ED to EDE to EDEA to EGEDE/GEDEA and now to EGEDEA. The αCOO− ν(C=O) band position is slightly red shifted from the EGEDE pentapeptide, again holding with the trend observed in the αCOO− ν(C=O) band position moving from EG to EGE to EGED to EGEDE and now to EGEDEA. Again, very little variation is observed in the αCOO− ν(C=O) band position when moving from ED to EDE to EDEA to GEDEA and on to EGEDEA.

#### 3.2.2. Intramolecular Charge Transfer Disparately Influences Amide I and αCOO− Vibrations

**Figure 4:**
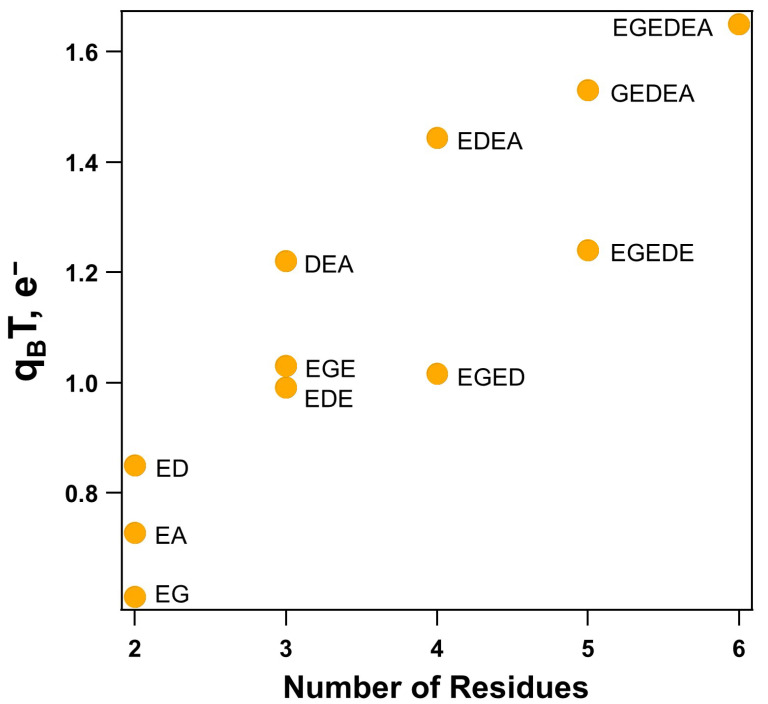
Correlation of the computed Boltzmann-weighted sum of the total magnitude of the intramolecular charge transfer, q_B_T, as the size, *n*, of the peptide chain increases from *n* = 2 to *n* = 6, presented in millielectrons, e^−^.

As shown in Table 2 and Figure 4, the extent of intramolecular hydrogen bonding increases as the size of the peptide chain increases, while the extent of intramolecular hydrogen bonding in each size class depends on the amino acid composition. The magnitude of intramolecular charge transfer in these systems does not follow the same trends shown in the amide I and αCOO− ν(C=O) band positions, except in the dipeptide and tetrapeptide cases. Of the dipeptides, all three fragments exhibit three intramolecular hydrogen bonds, with the most nonpolar dipeptide fragment, EG, exhibiting the smallest extent of intramolecular hydrogen bonding, q_B_T of 0.611 e^−^, and the most polar dipeptide, ED, exhibiting the greatest extent of intramolecular hydrogen bonding, with a q_B_T of 0.850 e^−^. This is also reminiscent of the additional carboxylic acid found in the ED fragment and not in the EG and EA fragments.

For the tripeptides, all three exhibit a greater extent of intramolecular charge transfer than the dipeptides. The most polar DEA tripeptide possesses the greatest magnitude of charge transfer and five intramolecular hydrogen bonds, q_B_T of 1.220 e^−^. The less polar tripeptides, EGE and EDE, exhibit a magnitude of 1.030 e^−^ and 0.990 e^−^, respectively, transferred within the system, both displaying only four intramolecular hydrogen bonds. Considering the molecular geometries, all three carboxylic acids in the DEA tripeptide participate in intramolecular hydrogen bonds, with protonation observed in D residue’s carboxylic acid by the nearby αNH_3_^+^ group. This leads to the formation of a strong hydrogen bond (0.400 e^−^) between the protonated O-H and the now neutral αNH_2_ group. In the EGE tripeptide, again all three of the carboxylic acids are shown to participate in intramolecular hydrogen bonds. However, no protonation occurs, and the magnitudes of the formed hydrogen bonds are not as great as those in the DEA tripeptide. The EDE tripeptide is considered the most polar of the three, but, unlike the dipeptides, this fragment exhibits the smallest extent of intramolecular charge transfer, which is surprising considering the stabilization observed in the vibrational frequencies discussed above. This is possibly due to the fact that although there is an additional carboxylic acid present in this fragment, it does not participate in an intramolecular hydrogen bond, and, thus, there is no computed increase in the magnitude of intramolecular charge transfer.

Of the two tetrapeptides considered, the most polar, EDEA, exhibits the greatest magnitude of intramolecular charge transfer (q_B_T of 1.444 e^−^). This behavior is unsurprising considering the formation of seven intramolecular hydrogen bonds when compared to the EGED tetrapeptide (q_B_T of 1.016 e^−^), which forms only four. EGED is predicted with protonation in D residue’s carboxylic acid by the nearby αNH_3_^+^ group. Despite the protonation and formation of a strong hydrogen bond between the protonated O−H and the now neutral αNH_2_ group (0.39 e^−^), interruption of the polar EED residues by the nonpolar G residue dramatically reduces the ability of the side chains to form intramolecular hydrogen bonds with each other, which is also observed in the shifts of the amide I and αCOO− ν(C=O) band positions described above. EGED exhibits even less charge transfer than the EGE tripeptide, explaining the low energy position of the amide I band when compared to the rest of the fragments (Figure 4). EDEA falls in line with a trend arising from a steady increase in the magnitude of intramolecular charge transfer from ED/EA to DEA to EDEA.

The pentapeptide, GEDEA, also falls into this trend, with the formation of six intramolecular hydrogen bonds and a q_B_T of 1.530 e^−^. The carboxylic acid of the first E residue in GEDEA is predicted to be protonated by the nearby αNH_3_^+^ group, leading to the formation of a strong hydrogen bond between the protonated O-H and the now neutral αNH_2_ group (0.39 e^−^); however, in this case, there is no interruption of the remaining three residues, which all participate in at least one intramolecular hydrogen bond with the peptide backbone and, in the E2 residues case, two stabilizing hydrogen bonds with the backbone (both at 0.26 e^−^). The EGEDE pentapeptide also displays six intramolecular hydrogen bonds, but the magnitude of intramolecular charge transfer is less, with a q_B_T of 1.240 e^−^. The EGEDE pentapeptide is also protonated with the third E residue’s carboxylic acid interacting with the αNH_3_^+^ group, again leading to the formation of a strong hydrogen bond between the protonated O-H and the now neutral αNH_2_ group (0.38 e^−^). The first E residue’s carboxylic acid does not participate in intramolecular hydrogen bonding, leading to the overall lower magnitude of charge transfer observed in EGEDE compared to GEDEA. EGEDE falls in line well with a trend arising for a steady increase in the magnitude of intramolecular hydrogen e^−^ bonded charge transfer moving from the EG and ED dipeptides to EDE and EGE tripeptides to the EGED tetrapeptide and now to the EGEDE pentapeptide. In the hexapeptide case, EGEDEA, seven intramolecular hydrogen bonds are formed and a total of 1.650 e^−^ are transferred in the system.

The hexapeptide again exhibits protonation, this time of the third E residue’s carboxylic acid by the αNH_3_^+^ group, with the formation of the strongest computed hydrogen bond in this study of 0.401 e^−^ between the protonated O-H and the now neutral αNH_2_ group. The trends observed in the magnitude of intramolecular hydrogen bonded charge transfer appear to depend upon the nature of the polarity of the amino acids in the peptide chain, the protonation state of the αNH_3_^+^ group, and the length of the peptide chain, with overall more polar and longer fragments exhibiting an observably greater extent of intramolecular charge transfer.

### 3.3. Experimental and Computational Vibrational Analyses Predict EGEDEA Secondary Structure

**Figure 5:**
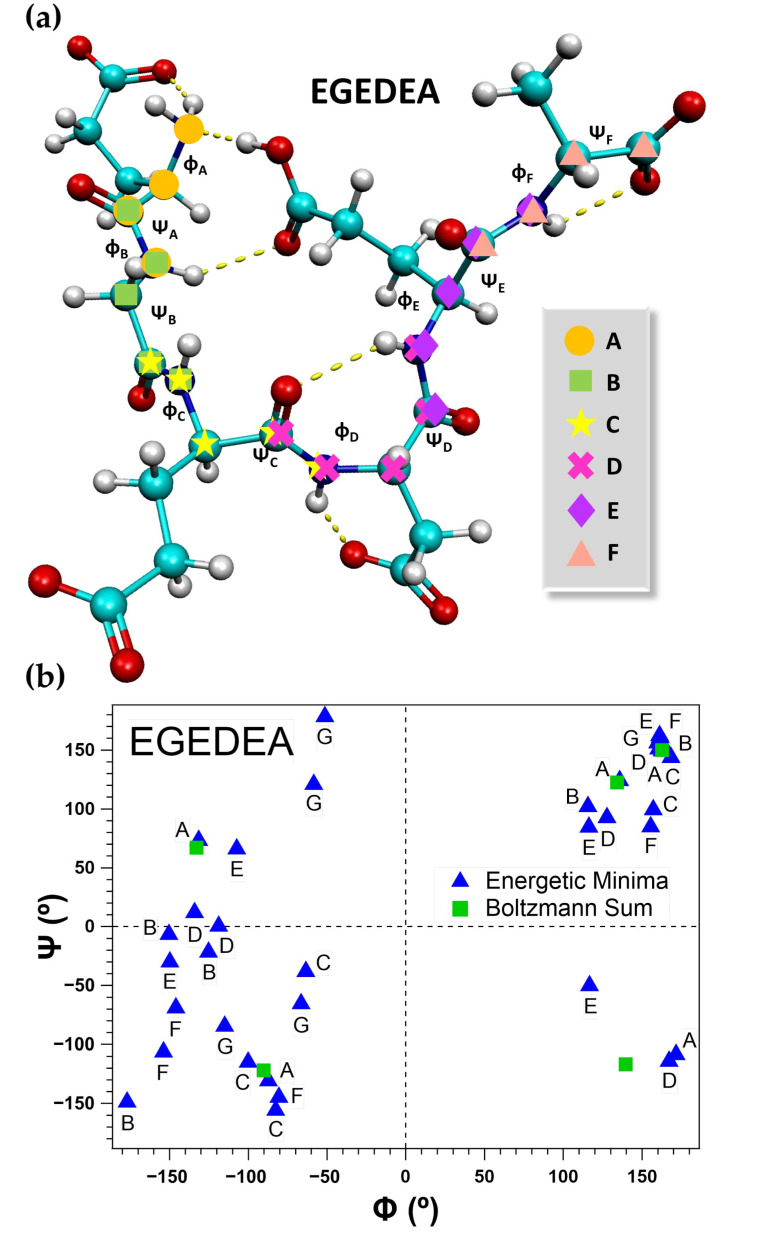
(**a**) Computed molecular geometry of the lowest energy conformation of EGEDEA, A, with the assigned Φ and Ψ bond angles (presented in degrees, °) labeled for each peptide bond. (**b**) Ramachandran plot of the computed φ and ψ bond angles for each of the considered energetic minima of the EGEDEA hexapeptide, presented in degrees (°). The Boltzmann sums of the weighted values are also presented.

The E-hook of β-tubulin protrudes from the microtubule surface and is thought to adopt a mostly disordered structure due to the inability to resolve its crystal structure. Hence, there is no current expectation for the peptide to fall under typical secondary structure parameters. Figure 5a presents the molecular geometry for the lowest energy conformation of the EGEDEA hexapeptide computed here, structure A, with the Φ and Ψ bond angles labeled for each peptide bond in °. Figure 5b is a Ramachandran plot of the computed φ and ψ bond angles for each of the energetic minima found for the EGEDEA hexapeptide (Figure S11), along with the sums of the Boltzmann-weighted angles. The Ramachandran plots for the rest of the fragments considered in this study can be found in the Supporting Information, Figures S24–S33.

Considering only Boltzmann sums of the φ and ψ bond angles and the Boltzmann-weighted position of the amide I ν(C=O) band predicted at 1658 cm^−1^ (experiment also at 1658 cm^−1^), the EGEDEA hexapeptide appears to take on a helical conformation in the crystalline state, with the mean frequency for α-helical structures reported at 1652 cm^−1^ for the amide I band [7]. The φ and ψ angles that appear in the restricted region (+120°, −120°) in proteins traditionally belong to G residues, as the lack of substitution on the C_α_ permits a greater extent of flexibility about the peptide bond when compared to other amino acid residues in the chain, as is also the case here. The rest of the angles that fall in the region are typically associated with α-helices; however, further analysis of the vibrational coupling constants in the amide I region is needed to reliably predict the EGEDEA secondary structure [7,53,54,55,114,115,116]. Although it is unlikely that the peptides form short helices without external stabilization, it is possible that a portion of the external α-helices would extend and specialize to perform E-hook’s duties. Additionally, interaction with the local environment and MAPs potentially support its helical structure.

### 3.4. Local Structure within E-Hooks May Play Pivotal Roles in Protein Recruitment and Retention

The full sequence of the βII isotype of tubulin is DATADEQGEFEEEEGEDEA with an 11- overall charge, 8 glutamates, and is 19 residues long [117]. If the overall peptide is considered disordered and has not been resolved in crystallographic structures, what is the role of the seemingly local helical structure of the C-terminal end? The very end of the long, disordered E-hook possibly acts as a local “hook” when binding microtubule (MT)-associated proteins (MAPs) or molecular motors, facilitating the delicate balance of having enough affinity to be recruited to the MT surface but not so tightly that it cannot exhibit motility. Further, if the spectroscopic clues from this study are used to dissect the remaining E-hook structure, there are sections of high electronegativity that appear broken up by segments of uncharged residues with varying hydrogen bonding potential. Only one such interruption appears in the EGEDEA hexapeptide, by the G residue, which results in the first three residues arranging in an extended linear backbone conformation. The first turn begins at the conjunction of the second E residue and D residue (shown in Figure 1), which is accompanied by a strong intramolecular hydrogen bond (0.273 e^−^) stabilizing the helical turn. The resulting residues in the EGEDEA hexapeptide appear to exhibit a greater extent of helical behavior than the first three.

The amide I vibration is shown to shift from 1656 to 1617 cm^−1^ (Table 2) when moving from EG to EGE, which disrupts the delocalization of electron density between the two glutamic acid residues. The addition of two more polar residues, creating EGEDE, results in the amide I vibrations shifting back to 1650 cm^−1^. The results here indicate that the amide I mode is highly influenced by the surrounding residues and the local hydrogen bonding environment, as would be expected for forming secondary structures. Furthermore, this suggests that these interruptions do not foster the proper environment for the E-hook to form higher order architectures on its own. However, when the peptide binds MAPs or molecular motors such as kinesin or dynein, perhaps this flexibility allows the E-hook to form customized attachments to these different proteins. For instance, the presence of E-hooks on MTs differentially affects binding and processivity of different kinesins, ranging from small modulations in motility to complete inhibition [63,64,75]. Additionally, E-hooks are the diversity site of tubulin, where the core is mostly conserved, but these disordered domains undergo a variety of post-translational modifications (PTMs), forming what is often referred to as the “tubulin code” [117,118]. In particular, β-tubulin E-hooks undergo glutamylation, tyrosination, and phosphorylation [117,118]. It is plausible that these additions further prevent the formation of secondary structures under physiological conditions, as well as custom-tune E-hook fit to motor and MAP binding sites. However, the effects of PTMs on local E-hook structure are not well understood and will be the subject of future study.

There are limitations to the method selected, including the potential that the global minimum was been identified; thus, future work will include additional computational models to search for the many conformations of the EGEDEA hexapeptide. This will allow for comparison to our method to determine its accuracy. Furthermore, this investigation does not consider the involvement of protein/peptide dynamics, which is vital due to E-hook’s key function as a “hook” for microtubule-associated proteins (MAPs). An analysis of the interaction of E-hook with the local environment in various solvent models is also a subject of future study, including implicit and explicit solvation of the hexapeptide in water.

## 4. Conclusions

In summary, a Boltzmann sum of the simulated Raman spectra compares well with the experiment to track the amide I and αCOO− ν(C=O) band positions for each in a family of l-glutamic acid-containing peptide fragments (EG, ED, EA, EGE, EDE, DEA, EGED, EDEA, EGEDE, GEDEA, and EGEDEA). The computational protocol presented here gives comparable results to the experiment, and, together, these tools provide deeper insights into the biophysical properties of these molecules of interest. The Raman experimental band position for the EGEDEA hexapeptide appears at 1658 cm^−1^, confirmed by the computed Boltzmann sum of the corresponding vibration (also predicted at 1658 cm^−1^). Similarly, the experimental band position for the EDEA tetrapeptide appears at 1641 cm^−1^, compared to the Boltzmann, scaled DFT computed band position at 1640 cm^−1^. This greatly improves upon the previous computed prediction of the amide I vibration for this fragment at 1705 cm^−1^.

A steady blue shift arises when moving linearly from ED to EDE to EDEA to EGEDE/GEDEA and finally to the EGEDEA hexapeptide. However, very little variation is observed in the αCOO− ν(C=O) band position when considering this group of fragments. Conversely, a steady red shift arises in the αCOO− ν(C=O) band position when moving from EG to EGE to EGED to EGEDE and finally to EGEDEA, while no clear trend arises in the amide I band positions for this group of fragments. The extent of intramolecular charge transfer is found to be heavily dependent on the overall polarity and size of the peptide fragment, with larger and more polar fragments exhibiting the greatest magnitude of intramolecular charge transfer. NBO computations reveal that the EGEDEA hexapeptide exhibits the greatest extent of intramolecular charge transfer (q_B_T of 1.650 e^−^) and forms seven intramolecular hydrogen bonds.

Although the E-hook of β-tubulin is currently thought to be an intrinsically disordered protein tail that protrudes from the microtubule surface, the computed φ and ψ bond angles of the EGEDEA hexapeptide suggest it takes on an α-helical secondary structure, confirmation of which will be the focus of future studies.

## Figures and Tables

**Table 1:** Relative energies of the energetic minima for the EG, ED, EA, EGE, EDE, DEA, EGED, EDEA, EGEDE, GEDEA, and EGEDEA peptide fragments, computed at the B3LYP/6-311++G(2*df*,2*pd*) level of theory. The number of intramolecular hydrogen bonds (HBs); Boltzmann probability (*N_i_*); Boltzmann-weighted magnitudes of the hydrogen bonds, in e^−^; Boltzmann-weighted total charge transferred into the system (qT); and the amide I and αCOO− terminal ν(C=O) bands and shifts for each structure are also computed, in cm^−1^.

Structure	ΔE	*N_i_*	HBs	αNH_3_^+^ H Bond, Δq, e^−^	αCOO− H Bond, Δq, e^−^	qT, e^−^	Amide I ν(C=O) Band, cm^−1^	αCOO− Terminal ν(C=O) Band, cm^−1^
EG-A	0.00	9.74 × 10^−1^	2	0.360	0.234	5.94 × 10^−1^	1655	1645
EG-B	2.39	1.73 × 10^−2^	2	0.006	0.003	9.70 × 10^−3^	−4	0
EG-C	3.03	5.94 × 10^−3^	3	0.002	0.002	5.28 × 10^−3^	+29	−6
EG-D	3.65	2.06 × 10^−3^	3	0.001	0.001	1.85 × 10^−3^	+26	−13
EG-E	4.04	1.07 × 10^−3^	2	0.000	0.000	6.71 × 10^−4^	−3	−1
EG-F	8.91	2.83 × 10^−7^	2	0.000	0.000	1.98 × 10^−7^	+43	+81
ED-A	0.00	9.99 × 10^−1^	3	0.270	0.250	8.49 × 10^−1^	1631	1609
ED-B	4.06	1.04 × 10^−3^	3	0.000	0.000	7.60 × 10^−4^	+34	+138
ED-C	4.95	2.33 × 10^−4^	4	0.000	0.000	1.72 × 10^−4^	+38	+146
ED-D	7.03	6.94 × 10^−6^	3	0.000	0.000	5.83 × 10^−6^	+21	−1
ED-E	9.45	1.16 × 10^−7^	3	0.000	0.000	1.03 × 10^−7^	+37	+4
ED-F	9.69	7.81 × 10^−8^	3	0.000	0.000	6.64 × 10^−8^	+39	+3
EA-A	0.00	9.71 × 10^−1^	3	0.359	0.243	7.09 × 10^−1^	1651	1642
EA-B	2.13	2.64 × 10^−2^	2	0.010	0.007	1.63 × 10^−2^	−2	0
EA-C	3.49	2.66 × 10^−3^	3	0.001	0.001	2.34 × 10^−3^	+28	−14
EA-D	7.08	6.24 × 10^−6^	3	0.000	0.000	3.68 × 10^−6^	+2	+116
EA-E	12.27	9.63 × 10^−^^10^	3	0.000	0.000	8.09 × 10^−^^10^	+29	+125
EA-F	24.01	2.35 × 10^−^^18^	1	0.000	---	9.15 × 10^−^^19^	+61	+92
EGE-A	0.00	1.00	4	0.290	0.240	1.03	1617	1641
EGE-B	10.13	3.70 × 10^−8^	2	0.000	0.000	2.30 × 10^−8^	+9	+44
EGE-C	16.59	6.73 × 10^−^^13^	4	0.000	0.000	6.19 × 10^−^^13^	+47	−13
EGE-D	17.14	2.67 × 10^−^^13^	4	0.000	0.000	2.43 × 10^−^^13^	+38	−15
EGE-E	19.73	3.33 × 10^−^^15^	4	0.000	0.000	2.99 × 10^−^^15^	+50	−18
EGE-F	24.09	2.10 × 10^−^^18^	4	0.000	0.000	2.23 × 10^−^^18^	+85	−11
EDE-A	0.00	9.97 × 10^−1^	4	0.279	0.249	9.87 × 10^−1^	1634	1609
EDE-B	3.64	2.14 × 10^−3^	4	0.001	0.001	2.25 × 10^−3^	−9	+4
EDE-C	4.46	5.31 × 10^−4^	5	0.000	0.000	6.68 × 10^−4^	+4	−2
EDE-D	5.03	2.02 × 10^−4^	5	0.000	0.000	2.36 × 10^−4^	+3	−2
EDE-E	7.89	1.62 × 10^−6^	3	0.000	0.000	1.47 × 10^−6^	+24	−2
EDE-F	12.00	1.57 × 10^−9^	3	0.000	0.000	1.45 × 10^−9^	+1	−2
DEA-A	0.00	9.99 × 10^−1^	5	0.399	0.240	1.22	1635	1618
DEA-B	4.16	8.79 × 10^−4^	3	0.000	0.000	8.27 × 10^−4^	+8	+4
DEA-C	4.54	4.69 × 10^−4^	4	0.000	0.000	4.60 × 10^−4^	−2	+10
DEA-D	6.60	1.43 × 10^−5^	4	0.000	0.000	1.54 × 10^−5^	−21	+6
DEA-E	7.89	1.62 × 10^−6^	3	0.000	0.000	1.15 × 10^−6^	+28	+8
DEA-F	13.89	6.39 × 10^−^^11^	3	0.000	0.000	5.75 × 10^−^^11^	−14	−1
EGED-A	0.00	9.88 × 10^−1^	4	0.385	0.227	1.01	1676	1620
EGED-B	3.02	6.04 × 10^−3^	3	0.002	0.001	3.51 × 10^−3^	−1	+30
EGED-C	3.44	2.93 × 10^−3^	4	0.001	0.001	2.49 v 10^−3^	−16	+11
EGED-D	3.71	1.87 × 10^−3^	4	0.001	0.000	1.48 × 10^−3^	+4	+13
EGED-E	4.29	7.09 × 10^−4^	4	0.000	0.000	6.31 × 10^−4^	−7	+13
EGED-F	4.68	3.66 × 10^−4^	2	0.000	---	2.20 × 10^−4^	+1	+107
EDEA-A	0.00	9.29 × 10^−1^	7	0.260	0.223	1.34	1639	1604
EDEA-B	1.53	7.05 × 10^−2^	6	0.022	0.016	1.05 × 10^−1^	+13	+2
EDEA-C	7.87	1.55 × 10^−6^	5	0.000	0.000	1.91 × 10^−6^	+3	+8
EDEA-D	16.11	1.41 × 10^−^^12^	5	0.000	0.000	1.79 × 10^−^^12^	+32	−3
EDEA-E	34.60	3.84 × 10^−^^26^	6	0.000	0.000	5.42 × 10^−^^26^	+69	+11
EDEA-F	47.14	2.40 × 10^−^^35^	5	0.000	---	2.96 × 10^−^^35^	+100	+5
EGEDE-A	0.00	1.00	6	0.380	0.230	1.24	1650	1614
EGEDE-B	4.52	4.83 × 10^−4^	5	0.000	0.000	4.49 × 10^−4^	+9	+11
EGEDE-C	8.65	4.51 × 10^−7^	5	0.000	0.000	4.29 × 10^−7^	+32	−5
EGEDE-D	11.67	2.72 × 10^−9^	6	0.000	0.000	3.40 × 10^−9^	+2	+83
EGEDE-E	11.70	2.62 × 10^−9^	6	0.000	0.000	3.77 × 10^−9^	+8	−21
EGEDE-F	32.43	1.59 × 10^−^^24^	6	0.000	0.000	2.28 × 10^−^^24^	+48	−9
GEDEA-A	0.00	1.00	6	0.380	0.230	1.53	1654	1607
GEDEA-B	5.92	4.55 × 10^−5^	4	0.000	0.000	5.60 × 10^−5^	+7	+3
GEDEA-C	11.41	4.23 × 10^−9^	5	0.000	0.000	4.40 × 10^−9^	+19	+11
GEDEA-D	11.47	3.86 × 10^−9^	6	0.000	0.000	4.86 × 10^−9^	+18	−2
GEDEA-E	21.81	9.90 × 10^−^^17^	4	0.000	---	9.11 × 10^−^^17^	+15	+106
GEDEA-F	27.11	1.28 × 10^−^^20^	6	0.000	0.000	1.42 × 10^−^^20^	+52	+29
EGEDEA-A	0.00	9.20 × 10^−1^	7	0.368	0.221	1.51	1658	1610
EGEDEA-B	1.44	8.02 × 10^−2^	7	0.033	0.019	1.41 × 10^−1^	−1	+1
EGEDEA-C	11.19	5.70 × 10^−9^	6	0.000	0.000	8.84 × 10^−9^	+16	−6
EGEDEA-D	11.35	4.34 × 10^−9^	6	0.000	0.000	6.25 × 10^−9^	+7	−8
EGEDEA-E	11.73	2.29 × 10^−9^	6	0.000	0.000	3.55 × 10^−9^	−5	−6
EGEDEA-F	17.75	8.72 × 10^−^^14^	6	0.000	0.000	1.19 × 10^−^^13^	+7	−4

**Table 2:** Boltzmann sums of the total charge transferred via intramolecular hydrogen bonding (q_B_T), in millielectrons, e^−^, along with the Boltzmann sums of the amide I and αCOO− terminal ν(C=O) band positions for each peptide fragment of interest compared to the experimental Raman band positions, in wavenumbers, cm^−1^, from our previous investigation. Data reproduced with permission under license 5121961040490.

Structure	q_B_T	Boltzmann Sum of the Amide I ν(C=O) Band, cm^−1^	Experimental Amide I ν(C=O) Band, cm^−1^ [113]	Boltzmann Sum of the αCOO− Terminal ν(C=O) Band, cm^−1^	Experimental αCOO− Terminal ν(C=O) Band, cm^−1^ [113]
EG	0.611	1656	1671	1644	1648
ED	0.850	1631	1632	1609	1608
EA	0.727	1651	1651	1642	1628
EGE	1.030	1617	---	1641	---
EDE	0.990	1634	---	1609	---
DEA	1.220	1635	---	1618	---
EGED	1.016	1676	1686	1620	1622
EDEA	1.444	1640	1641	1605	1602
EGEDE	1.240	1650	---	1614	---
GEDEA	1.530	1654	---	1607	---
EGEDEA	1.650	1658	1658	1610	1611

## Data Availability

The authors confirm that all data are presented in the article and supplementary information.

## Supplementary Materials

The following are available online. Figures S1–S33 and Tables S1–S22.

## References

[1] Anfinsen C.B. (1973). Principles that Govern the Folding of Protein Chains. Science.

[2] Fleming P.J., Rose G.D. (2005). Do all backbone polar groups in proteins form hydrogen bonds?. Protein Sci..

[3] Pauling L., Corey R.B., Branson H.R. (1951). The structure of proteins: Two hydrogen-bonded helical configurations of the polypeptide chain. Proc. Natl. Acad. Sci. USA.

[4] Mikshiev V.Y., Pozharskii A.F., Filarowski A., Novikov A.S., Antonov A.S., Tolstoy P.M., Vovk M.A., Khoroshilova O.V. (2020). How Strong is Hydrogen Bonding to Amide Nitrogen?. Chemphyschem.

[5] Cox C., Wack H., Lectka T. (1999). Strong Hydrogen Bonding to the Amide Nitrogen Atom in an “Amide Proton Sponge”: Consequences for Structure and Reactivity. Angew. Chem. Int. Ed..

[6] Giubertoni G., Sofronov O.O., Bakker H.J. (2020). Effect of intramolecular hydrogen-bond formation on the molecular conformation of amino acids. Commun. Chem..

[7] Nevskaya N.A., Chirgadze Y.N. (1976). Infrared spectra and resonance interactions of amide-I and II vibrations of α-helix. Biopolymers.

[8] Mandal I., Paul S., Venkatramani R. (2018). Optical backbone-sidechain charge transfer transitions in proteins sensitive to secondary structure and modifications. Faraday Discuss..

[9] Myshakina N.S., Ahmed Z., Asher S.A. (2008). Dependence of Amide Vibrations on Hydrogen Bonding. J. Phys. Chem. B.

[10] Williams A.E., Davis J.E., Reynolds J.E., Fortenberry R.C., Hammer N.I., Reinemann D.N. (2020). Determination of Vibrational Band Positions in the E-Hook of β-Tubulin. Spectrochim. Acta A Mol. Biomol. Spectrosc..

[11] Rybka K., Toal S.E., Verbaro D.J., Mathieu D., Schwalbe H., Schweitzer-Stenner R. (2013). Disorder and order in unfolded and disordered peptides and proteins: A view derived from tripeptide conformational analysis. II. Tripeptides with short side chains populating asx and β-type like turn conformations. Proteins Struct. Funct. Bioinform..

[12] Ramos J., Cruz V.L. (2016). Conformational analysis of short polar side-chain amino-acids through umbrella sampling and DFT calculations. J. Mol. Modeling.

[13] Pogostin B.H., Malmendal A., Londergan C.H., Åkerfeldt K.S. (2019). pKa Determination of a Histidine Residue in a Short Peptide Using Raman Spectroscopy. Molecules.

[14] Ryall J.P., Dines T.J., Chowdhry B.Z., Leharne S.A., Withnall R. (2010). Vibrational spectra and structures of urazole and 4-methylurazole: DFT calculations of the normal modes in aqueous solution and in the solid state, and the influence of hydrogen bonding. Chem. Phys..

[15] Huang S.R., Liu Y., Tureček F. (2019). Non-covalent complexes of the peptide fragment Gly-Asn-Asn-Gln-Gln-Asn-Tyr in the gas-phase. Photodissociative cross-linking, Born–Oppenheimer molecular dynamics, and ab initio computational binding study. Phys. Chem. Chem. Phys..

[16] Silva C.B., da Silva Filho J.G., Pinheiro G.S., Teixeira A.M.R., de Sousa F.F., Freire P.T.C. (2020). High-pressure studies on l,l-dileucine crystals by Raman spectroscopy and synchrotron X-ray diffraction combined with DFT calculations. Spectrochim. Acta A Mol. Biomol. Spectrosc..

[17] Kecel-Gunduz S., Bicak B., Celik S., Akyuz S., Ozel A.E. (2017). Structural and spectroscopic investigation on antioxidant dipeptide, l-Methionyl-l-Serine: A combined experimental and DFT study. J. Mol. Struct..

[18] Derbel N., Hernández B., Pflüger F., Liquier J., Geinguenaud F., Jaïdane N., Ben Lakhdar Z., Ghomi M. (2007). Vibrational Analysis of Amino Acids and Short Peptides in Hydrated Media. I. l-glycine and l-leucine. J. Phys. Chem. B.

[19] Buchanan E.G., James W.H., Choi S.H., Guo L., Gellman S.H., Müller C.W., Zwier T.S. (2012). Single-conformation infrared spectra of model peptides in the amide I and amide II regions: Experiment-based determination of local mode frequencies and inter-mode coupling. J. Chem. Phys..

[20] Bhunia S., Srivastava S.K., Materny A., Ojha A.K. (2016). A vibrational and conformational characterization of arginine at different pH values investigated using Raman spectroscopy combined with DFT calculations. J. Raman Spectrosc..

[21] Eker F., Cao X., Nafie L., Schweitzer-Stenner R. (2002). Tripeptides Adopt Stable Structures in Water. A Combined Polarized Visible Raman, FTIR, and VCD Spectroscopy Study. J. Am. Chem. Soc..

[22] Walsh P.S., Dean J.C., McBurney C., Kang H., Gellman S.H., Zwier T.S. (2016). Conformation-specific spectroscopy of capped glutamine-containing peptides: Role of a single glutamine residue on peptide backbone preferences. Phys. Chem. Chem. Phys..

[23] Habka S., Sohn W.Y., Vaquero-Vara V., Géléoc M., Tardivel B., Brenner V., Gloaguen E., Mons M. (2018). On the turn-inducing properties of asparagine: The structuring role of the amide side chain, from isolated model peptides to crystallized proteins. Phys. Chem. Chem. Phys..

[24] Martial B., Lefèvre T., Auger M. (2018). Understanding amyloid fibril formation using protein fragments: Structural investigations via vibrational spectroscopy and solid-state NMR. Biophys. Rev..

[25] Jalkanen K.J., Suhai S. (1996). N-Acetyl-l-alanine N′-methylamide: A density functional analysis of the vibrational absorption and vibrational circular dichroism spectra. Chem. Phys..

[26] Jalkanen K.J., Elstner M., Suhai S. (2004). Amino acids and small peptides as building blocks for proteins: Comparative theoretical and spectroscopic studies. J. Mol. Struct. THEOCHEM.

[27] Correia C.F., Balaj P.O., Scuderi D., Maitre P., Ohanessian G. (2008). Vibrational Signatures of Protonated, Phosphorylated Amino Acids in the Gas Phase. J. Am. Chem. Soc..

[28] Bour P., Kubelka J., Keiderling T.A. (2002). Ab initio quantum mechanical models of peptide helices and their vibrational spectra. Biopolymers.

[29] Woutersen S., Pfister R., Hamm P., Mu Y., Kosov D.S., Stock G. (2002). Peptide conformational heterogeneity revealed from nonlinear vibrational spectroscopy and molecular-dynamics simulations. J. Chem. Phys..

[30] Lee K.-K., Oh K.-I., Lee H., Joo C., Han H., Cho M. (2007). Dipeptide Structure Determination by Vibrational Circular Dichroism Combined with Quantum Chemistry Calculations. Chemphyschem.

[31] Kolev T., Spiteller M., Koleva B. (2010). Spectroscopic and structural elucidation of amino acid derivatives and small peptides: Experimental and theoretical tools. Amino Acids.

[32] Kobko N., Dannenberg J.J. (2003). Cooperativity in Amide Hydrogen Bonding Chains. A Comparison between Vibrational Coupling through Hydrogen Bonds and Covalent Bonds. Implications for Peptide Vibrational Spectra. J. Phys. Chem. A.

[33] Pires D.A.T., Arake L.M.R., Silva L.P., Lopez-Castillo A., Prates M.V., Nascimento C.J., Bloch C. (2018). A previously undescribed hexapeptide His-Arg-Phe-Leu-Arg-His-NH2 from amphibian skin secretion shows CO_2_ and metal biding affinities. Peptides.

[34] Furić K., Mohaček Grošev V., Bonifacic M., Štefanić I. (1992). Raman spectroscopic study of H_2_O and D_2_O water solutions of glycine. J. Mol. Struct..

[35] Navarrete J.T.L., Hernández V., Ramírez F.J. (1995). Vibrational study of aspartic acid and glutamic acid dipeptides. J. Mol. Struct..

[36] Parameswari A., Premkumar S., Premkumar R., Milton Franklin Benial A. (2016). Surface enhanced Raman spectroscopy and quantum chemical studies on glycine single crystal. J. Mol. Struct..

[37] Mendham A.P., Dines T.J., Snowden M.J., Chowdhry B.Z., Withnall R. (2009). Vibrational spectroscopy and DFT calculations of di-amino acid cyclic peptides. Part I: Cyclo(Gly-Gly), cyclo(L-Ala-l-Ala) and cyclo(L-Ala-Gly) in the solid state and in aqueous solution. J. Raman Spectrosc..

[38] Zhu G., Zhu X., Fan Q., Wan X. (2011). Raman spectra of amino acids and their aqueous solutions. Spectrochim. Acta A Mol. Biomol. Spectrosc..

[39] Barron L.D., Gargaro A.R., Hecht L., Polavarapu P.L. (1991). Experimental and ab initio theoretical vibrational Raman optical activity of alanine. Spectrochim. Acta Part A Mol. Spectrosc..

[40] Rožman M. (2007). Aspartic Acid Side Chain Effect—Experimental and Theoretical Insight. J. Am. Soc. Mass Spectrom..

[41] Kausar N., Dines T.J., Chowdhry B.Z., Alexander B.D. (2009). Vibrational spectroscopy and DFT calculations of the di-amino acid peptide l-aspartyl-l-glutamic acid in the zwitterionic state. Phys. Chem. Chem. Phys..

[42] Peica N., Lehene C., Leopold N., Schlücker S., Kiefer W. (2007). Monosodium glutamate in its anhydrous and monohydrate form: Differentiation by Raman spectroscopies and density functional calculations. Spectrochim. Acta A Mol. Biomol. Spectrosc..

[43] Navarrete J.T.L., Hernández V., Ramírez F.J. (1994). Vibrational spectra of [15N]glutamic acid and [2H4]glutamic acid. J. Raman Spectrosc..

[44] Bouř P., Sopková J., Bednárová L., Maloň P., Keiderling T.A. (1997). Transfer of molecular property tensors in cartesian coordinates: A new algorithm for simulation of vibrational spectra. J. Comput. Chem..

[45] Bouř P., Keiderling T.A. (2002). Partial optimization of molecular geometry in normal coordinates and use as a tool for simulation of vibrational spectra. J. Chem. Phys..

[46] Jacob C.R., Reiher M. (2009). Localizing normal modes in large molecules. J. Chem. Phys..

[47] Herrmann C., Neugebauer J., Reiher M. (2007). Finding a needle in a haystack: Direct determination of vibrational signatures in complex systems. New J. Chem..

[48] Herrmann C., Ruud K., Reiher M. (2008). Importance of backbone angles versus amino acid configurations in peptide vibrational Raman optical activity spectra. Chem. Phys..

[49] Jacob C.R., Luber S., Reiher M. (2009). Analysis of Secondary Structure Effects on the IR and Raman Spectra of Polypeptides in Terms of Localized Vibrations. J. Phys. Chem. B.

[50] Kumar S., Mishra K.K., Singh S.K., Borish K., Dey S., Sarkar B., Das A. (2019). Observation of a weak intra-residue C5 hydrogen-bond in a dipeptide containing Gly-Pro sequence. J. Chem. Phys..

[51] Gorbitz C. (2010). Structures of dipeptides: The head-to-tail story. Acta Crystallogr. Sect. B.

[52] Hanyu M., Ninomiya D., Yanagihara R., Murashima T., Miyazawa T., Yamada T. (2005). Studies on intramolecular hydrogen bonding between the pyridine nitrogen and the amide hydrogen of the peptide: Synthesis and conformational analysis of tripeptides containing novel amino acids with a pyridine ring. J. Pept. Sci..

[53] Torii H., Tasumi M. (1998). Ab Initio Molecular Orbital Study of the Amide I Vibrational Interactions between the Peptide Groups in Di- and Tripeptides and Considerations on the Conformation of the Extended Helix. J. Raman Spectrosc..

[54] Barth A., Zscherp C. (2002). What vibrations tell about proteins. Q. Rev. Biophys..

[55] Wieczorek R., Dannenberg J.J. (2003). Hydrogen-Bond Cooperativity, Vibrational Coupling, and Dependence of Helix Stability on Changes in Amino Acid Sequence in Small 310-Helical Peptides. A Density Functional Theory Study. J. Am. Chem. Soc..

[56] Wang Y.-F., Yu Z.-Y., Wu J., Liu C.-B. (2009). Electron Delocalization and Charge Transfer in Polypeptide Chains. J. Phys. Chem. A.

[57] Vener M.V., Egorova A.N., Fomin D.P., Tsirelson V.G. (2010). DFT study of H-bonds in the peptide secondary structures: The backbone–side-chain and polar side-chains interactions. J. Mol. Struct..

[58] Prell J.S., O’Brien J.T., Steill J.D., Oomens J., Williams E.R. (2009). Structures of Protonated Dipeptides: The Role of Arginine in Stabilizing Salt Bridges. J. Am. Chem. Soc..

[59] Laurin Y., Eyer J., Robert C.H., Prevost C., Sacquin-Mora S. (2017). Mobility and Core-Protein Binding Patterns of Disordered C-Terminal Tails in β-Tubulin Isotypes. Biochemistry.

[60] Okada Y., Hirokawa N. (2000). Mechanism of the single-headed processivity: Diffusional anchoring between the K-loop of kinesin and the C terminus of tubulin. Proc. Natl. Acad. Sci. USA.

[61] Reinemann D.N., Norris S.R., Ohi R., Lang M.J. (2018). Processive Kinesin-14 HSET Exhibits Directional Flexibility Depending on Motor Traffic. Curr. Biol..

[62] Reinemann D.N., Sturgill E.G., Das D.K., Degen M.S., Vörös Z., Hwang W., Ohi R., Lang M.J. (2017). Collective Force Regulation in Anti-parallel Microtubule Gliding by Dimeric Kif15 Kinesin Motors. Curr. Biol..

[63] Thorn K.S., Ubersax J.A., Vale R.D. (2000). Engineering the Processive Run Length of the Kinesin Motor. J. Cell Biol..

[64] Wang Z., Sheetz M.P. (2000). The C-terminus of tubulin increases cytoplasmic dynein and kinesin processivity. Biophys. J..

[65] Woehlke G., Ruby A.K., Hart C.L., Ly B., Hom-Booher N., Vale R.D. (1997). Microtubule Interaction Site of the Kinesin Motor. Cell.

[66] Dixit R., Ross J.L., Goldman Y.E., Holzbaur E.L.F. (2008). Differential Regulation of Dynein and Kinesin Motor Proteins by Tau. Science.

[67] Freedman H., Luchko T., Luduena R.F., Tuszynski J.A. (2011). Molecular Dynamics Modeling of Tubulin C-Terminal Tail Interactions with the Microtubule Surface. Proteins Struct. Funct. Bioinform..

[68] Gennerich A., Vale R.D. (2009). Walking the Walk: How Kinesin and Dynein Coordinate Their Steps. Curr. Opin. Cell Biol..

[69] Goldstein L.S.B. (2001). Kinesin molecular motors: Transport pathways, receptors, and human disease. Proc. Natl. Acad. Sci. USA.

[70] Heald R., Khodjakov A. (2015). Thirty years of search and capture: The complex simplicity of mitotic spindle assembly. J. Cell Biol..

[71] Janke C., Bulinski J.C. (2011). Post-Translational Regulation of the Microtubule Cytoskeleton: Mechanisms and Functions. Nat. Rev. Mol. Cell Biol..

[72] Karsenti E., Vernos I. (2001). The Mitotic Spindle: A Self-Made Machine. Science.

[73] Lakämper S., Meyhöfer E. (2005). The E-Hook of Tubulin Interacts with Kinesin’s Head to Increase Processivity and Speed. Biophys. J..

[74] Lansky Z., Braun M., Lüdecke A., Schlierf M., ten Wolde P.R., Janson M.E., Diez S. (2015). Diffusible Crosslinkers Generate Directed Forces in Microtubule Networks. Cell.

[75] Luchko T., Huzil J.T., Stepanova M., Tuszynski J. (2008). Conformational Analysis of the Carboxy-Terminal Tails of Human β-Tubulin Isotypes. Biophys. J..

[76] Nogales E. (2000). Structural Insights into Microtubule Function. Annu. Rev. Biochem..

[77] Otter A., Kotovych G. (1988). The Solution Conformation of the Synthetic Tubulin Fragment Ac-Tubulin-α (430–441)-Amide Based on Two-Dimensional ROESY Experiments. Can. J. Chem..

[78] Panneerselvam M., Muthu K., Jayaraman M., Sridharan U., Jenardhanan P., Ramadas K. (2013). Molecular Dynamic Simulations of the Tubulin-Human Gamma Synuclein Complex: Structural Insight into the Regulatory Mechanism Involved in Inducing Resistance against Taxol. Mol. Biosyst..

[79] Serrano L., de la Torre J., Maccioni R.B., Avila J. (1984). Involvement of the carboxyl-terminal domain of tubulin in the regulation of its assembly. Proc. Natl. Acad. Sci. USA.

[80] Sirajuddin M., Rice L.M., Vale R.D. (2014). Regulation of microtubule motors by tubulin isotypes and post-translational modifications. Nat. Cell Biol..

[81] Wall K.P., Pagratis M., Armstrong G., Balsbaugh J.L., Verbeke E., Pearson C.G., Hough L.E. (2016). Molecular Determinants of Tubulin’s C-Terminal Tail Conformational Ensemble. ACS Chem. Biol..

[82] Westermann S., Weber K. (2003). Post-Translational Modifications Regulate Microtubule Function. Nat. Rev. Mol. Cell Biol..

[83] Wordeman L. (2010). How kinesin motor proteins drive mitotic spindle function: Lessons from molecular assays. Semin. Cell Dev. Biol..

[84] Yildiz A., Tomishige M., Vale R.D., Selvin P.R. (2004). Kinesin Walks Hand-Over-Hand. Science.

[85] Sahu N., Gadre S.R. (2016). Vibrational infrared and Raman spectra of polypeptides: Fragments-in-fragments within molecular tailoring approach. J. Chem. Phys..

[86] Yamamoto S., Bouř P., Wójcik M.J., Nakatsuji H., Kirtman B., Ozaki Y. (2018). Calculation of Vibrational Spectra of Large Molecules from Their Fragments. Frontiers of Quantum Chemistry.

[87] Yamamoto S. (2012). Conformational analyses of peptides and proteins by vibrational Raman optical activity. Anal. Bioanal. Chem..

[88] Becke A.D. (1993). Density-Functional Thermochemistry. III. The Role of Exact Exchange. J. Chem. Phys..

[89] Krishnan R.B.J.S., Binkley J.S., Seeger R., Pople J.A. (1980). Self-Consistent Molecular Orbital Methods. XX. A Basis Set for Correlated Wave Functions. J. Chem. Phys..

[90] Lee C., Yang W., Parr R.G. (1988). Development of the Colle-Salvetti correlation-energy formula into a functional of the electron density. Phys. Rev. B.

[91] Moller C., Plesset M.S. (1934). Note on an Approximation Treatment for Many-Electron Systems. Phys. Rev..

[92] Glendening E.D., Reed A.E., Carpenter J.E., Weinhold F. (2001). NBO Version 3.1.

[93] Zhao Y., Truhlar D.G. (2008). The M06 Suite of Density Functionals for Main Group Thermochemistry, Thermochemical Kinetics, Noncovalent Interactions, Excited States, and Transition Elements: Two New Functionals and Systematic Testing of Four M06-Class Functionals and 12 Other Function. Theor. Chem. Acc..

[94] Perdew J.P., Burke K., Ernzerhof M. (1996). Generalized Gradient Approximation Made Simple. Phys. Rev. Lett..

[95] Frisch M.J., Trucks G.W., Schlegel H.B., Scuseria G.E., Robb M.A., Cheeseman J.R., Scalmani G., Barone V., Petersson G.A., Nakatsuji H. (2016). Gaussian 16 Rev. B.01.

[96] Huang K. (2010). Introduction to Statistical Physics.

[97] Tien C.L.L., John H. (1985). Statistical Thermodynamics.

[98] Andersson M.P., Uvdal P. (2005). New Scale Factors for Harmonic Vibrational Frequencies Using the B3LYP Density Functional Method with the Triple-ζ Basis Set 6-311+G(d,p). J. Phys. Chem. A.

[99] Baldauf C., Günther R., Hofmann H.-J. (2004). δ-Peptides and δ-Amino Acids as Tools for Peptide Structure DesignA Theoretical Study. J. Org. Chem..

[100] Echenique P., Chass G.A. (2008). Efficient model chemistries for peptides. II. Basis set convergence in the B3LYP method. arXiv.

[101] Jiménez-Hoyos C.A., Janesko B.G., Scuseria G.E. (2008). Evaluation of range-separated hybrid density functionals for the prediction of vibrational frequencies, infrared intensities, and Raman activities. Phys. Chem. Chem. Phys..

[102] Sjöberg B., Foley S., Cardey B., Enescu M. (2014). An experimental and theoretical study of the amino acid side chain Raman bands in proteins. Spectrochim. Acta A Mol. Biomol. Spectrosc..

[103] Tirado-Rives J., Jorgensen W.L. (2008). Performance of B3LYP Density Functional Methods for a Large Set of Organic Molecules. J. Chem. Theory Comput..

[104] Lelimousin M., Limongelli V., Sansom M.S.P. (2016). Conformational Changes in the Epidermal Growth Factor Receptor: Role of the Transmembrane Domain Investigated by Coarse-Grained MetaDynamics Free Energy Calculations. J. Am. Chem. Soc..

[105] Valdés H., Řeha D., Hobza P. (2006). Structure of isolated tryptophyl-glycine dipeptide and tryptophyl-glycyl- glycine tripeptide: Ab initio SCC-DFTB-D molecular dynamics simulations and high-level correlated ab initio quantum chemical calculations. J. Phys. Chem. B.

[106] Mortenson P.N., Wales D.J. (2001). Energy landscapes, global optimization and dynamics of the polyalanine Ac(ala)8NHMe. J. Chem. Phys..

[107] Papaleo E., Mereghetti P., Fantucci P., Grandori R., De Gioia L. (2009). Free-energy landscape, principal component analysis, and structural clustering to identify representative conformations from molecular dynamics simulations: The myoglobin case. J. Mol. Graph. Model..

[108] Christen M., Van Gunsteren W.F. (2008). On searching in, sampling of, and dynamically moving through conformational space of biomolecular systems: A review. J. Comput. Chem..

[109] Maximova T., Moffatt R., Ma B., Nussinov R., Shehu A. (2016). Principles and Overview of Sampling Methods for Modeling Macromolecular Structure and Dynamics. PLoS Comput. Biol..

[110] Wei G., Xi W., Nussinov R., Ma B. (2016). Protein Ensembles: How Does Nature Harness Thermodynamic Fluctuations for Life? the Diverse Functional Roles of Conformational Ensembles in the Cell. Chem. Rev..

[111] Riccardi L., Nguyen P.H., Stock G. (2012). Construction of the Free Energy Landscape of Peptide Aggregation from Molecular Dynamics Simulations. J. Chem. Theory Comput..

[112] Verma A., Wenzel W. (2009). A free-energy approach for all-atom protein simulation. Biophys. J..

[113] Lippert J.L., Tyminski D., Desmeules P.J. (1976). Determination of the Secondary Structure of Proteins by Laser Raman Spectroscopy. J. Am. Chem. Soc..

[114] Choi J.-H., Ham S., Cho M. (2003). Local Amide I Mode Frequencies and Coupling Constants in Polypeptides. J. Phys. Chem. B.

[115] Jansen T.l.C., Dijkstra A.G., Watson T.M., Hirst J.D., Knoester J. (2006). Modeling the amide I bands of small peptides. J. Chem. Phys..

[116] Rygula A., Majzner K., Marzec K.M., Kaczor A., Pilarczyk M., Baranska M. (2013). Raman spectroscopy of proteins: A review. J. Raman Spectrosc..

[117] Roll-Mecak A. (2015). Intrinsically disordered tubulin tails: Complex tuners of microtubule functions?. Semin. Cell Dev. Biol..

[118] Verhey K.J., Gaertig J. (2007). The Tubulin Code. Cell Cycle.

