# Supplementary material for: Tracking the Amide I and αCOO− Terminal ν(C=O) Raman Bands in a Family of l-Glutamic Acid-Containing Peptide Fragments: A Raman and DFT Study
Source: Molecules. 2021 Aug 7;26(16):4790. doi: 10.3390/molecules26164790 (PMC8399447; doi:10.3390/molecules26164790)
Supplement: Supplementary file 1 [file molecules-26-04790-s001.zip › molecules-1323150-SI.pdf]

Supporting Information for

“Tracking the Amide I and αCOO− Terminal ν(C=O) Raman Bands in a Family of L-Glutamic Acid Containing Peptide Fragments: A Raman and DFT Study”

Ashley E. Williams[1], Nathan I. Hammer[1], Ryan C. Fortenberry[1]*, and Dana N. Reinemann[2,3]*

[1]Department of Chemistry and Biochemistry, University of Mississippi, University, MS 38677. [2]Department of Biomedical Engineering, University of Mississippi, University, MS 38677. [3]Department of Chemical Engineering, University of Mississippi, University, MS 38677.

*Corresponding Authors: r410@olemiss.edu, dnreinem@olemiss.edu

Figure S1: Molecular geometries of the EG dipeptide fragment, computed at the B3LYP/6-311++G(2*df*,2*pd*) level of theory. Intramolecular hydrogen bonds are represented by dotted lines and the magnitudes are computed from natural bond order computations, presented in milielectrons, me−.

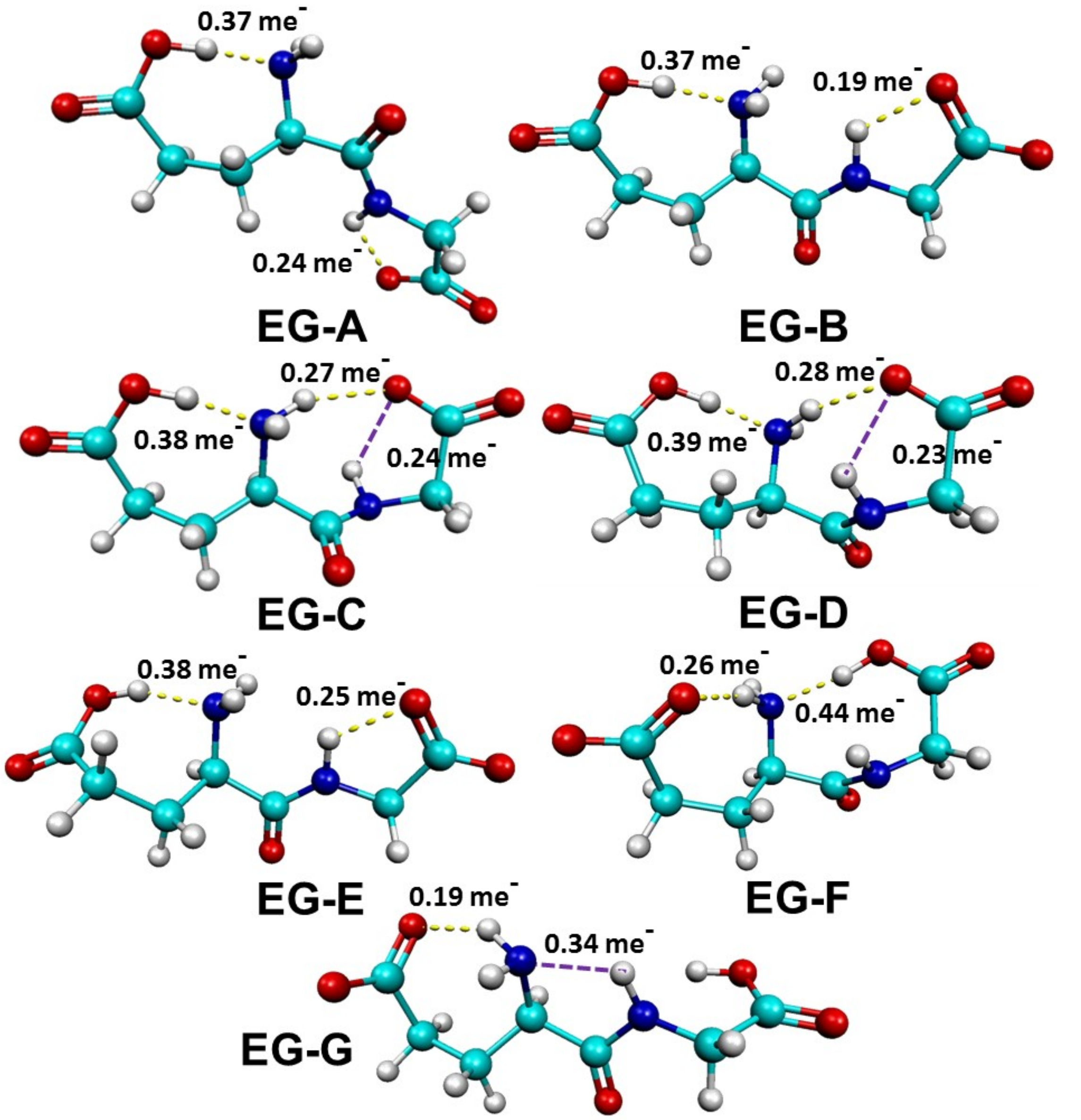

Table S1: Relative electronic energies of the EG dipeptide fragment, computed at the B3LYP/6-311++G(2*df*,2*pd*) level of theory. The magnitudes of the intramolecular hydrogen bonds are computed from natural bond order computations and are presented in milielectrons, me−. q_T represents the total charge transferred via intramolecular hydrogen bonding.

	**ΔE**	**N$_i$**	**HBs**	**αNH_3^+-H bond, Δq, me$^-$**	**αCOO⁻-H bond, Δq, me$^-$**	**Other H bond, Δq, me$^-$**	**qT**	**Amide I v(C=O) Band Position, cm^{-1}**	**αCOO⁻ Terminal v(C=O) Band Position, cm^{-1}**
**EG-A**	0.00	0.973630	2	0.37	0.24	---	0.61	1656	1644
**EG-B**	2.39	0.017313	2	0.37	0.19	---	0.56	1652	1644
**EG-C**	3.03	0.005936	3	0.38	0.27	0.24	0.89	1685	1638
**EG-D**	3.65	0.002055	3	0.39	0.28	0.23	0.90	1682	1632
**EG-E**	4.04	0.001066	2	0.38	0.25	---	0.63	1653	1643
**EG-F**	8.91	2.83E-07	2	0.26	0.44	---	0.70	1698	1725
**EG-G**	12.06	1.38E-09	2	0.19	0.34	---	0.53	1689	1773

Figure S2: Molecular geometries of the ED dipeptide fragment, computed at the B3LYP/6-311++G(2*df*,2*pd*) level of theory. Intramolecular hydrogen bonds are represented by dotted lines and the magnitudes are computed from natural bond order computations, presented in milielectrons, me−.

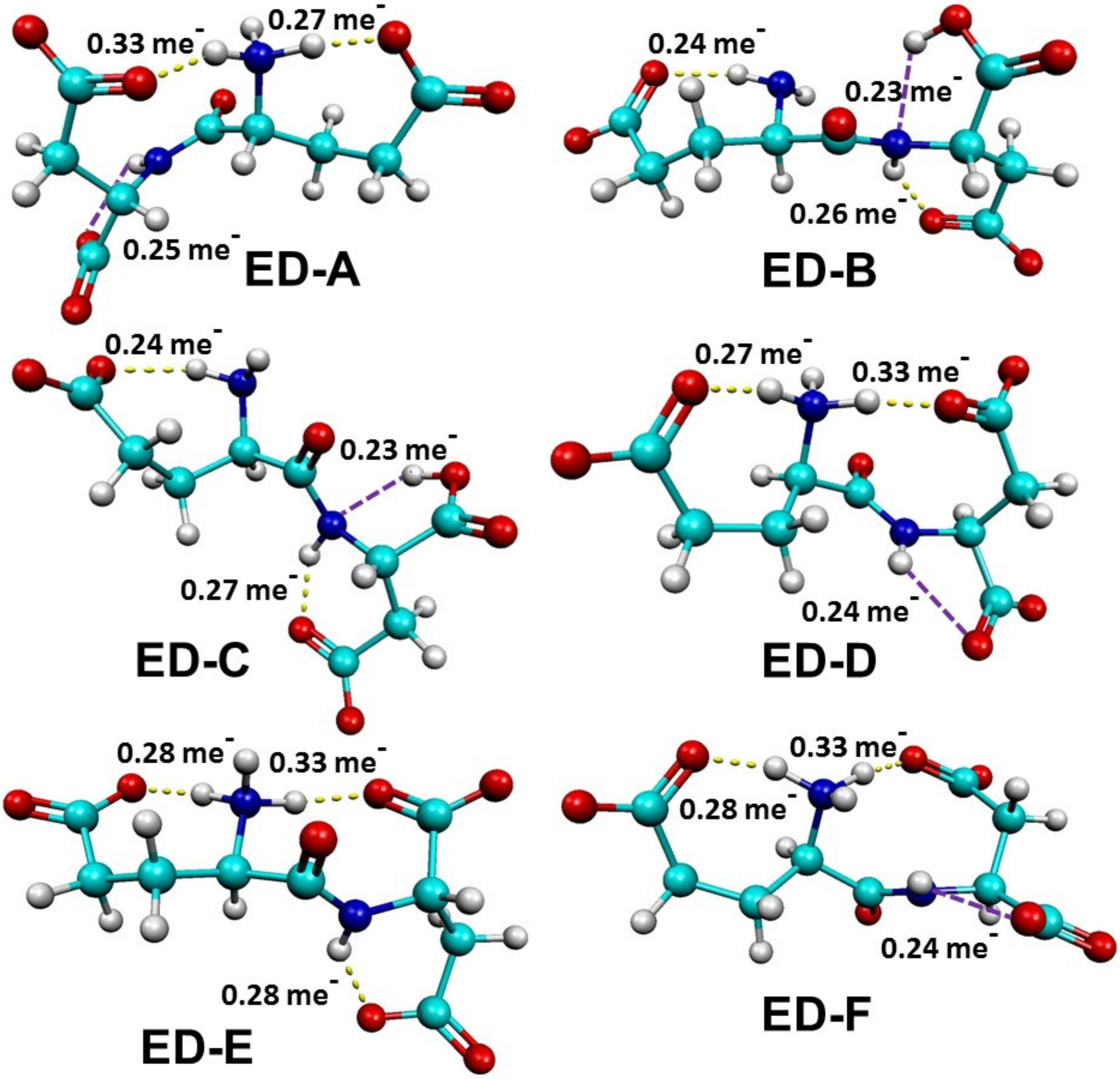

Table S2: Relative electronic energies of the ED dipeptide fragment, computed at the B3LYP/6-311++G(2*df*,2*pd*) level of theory. The magnitudes of the intramolecular hydrogen bonds are computed from natural bond order computations and are presented in milielectrons, me−. q_T represents the total charge transferred via intramolecular hydrogen bonding.

	**ΔE**	**N_i**	**HBs**	**αNH_3^+-H bond, Δq, me^-**	**αCOO^--H bond, Δq, me^-**	**Other H bond, Δq, me^-**	**qT**	**Amide I ν(C=O) Band Position, cm^{-1}**	**αCOO^- Terminal ν(C=O) Band Position, cm^{-1}**
**ED-A**	0.00	0.998719	3	0.27	0.25	0.33	0.85	1631	1609
**ED-B**	4.06	0.001041	3	0.24	0.23	0.26	0.73	1665	1747
**ED-C**	4.95	0.000233	4	0.24	0.23	0.27	0.74	1668	1756
**ED-D**	7.03	6.94E-06	3	0.27	0.24	0.33	0.84	1652	1608
**ED-E**	9.45	1.16E-07	3	0.28	0.33	0.28	0.89	1667	1613
**ED-F**	9.69	7.81E-08	3	0.28	0.24	0.33	0.85	1669	1612

Figure S3: Molecular geometries of the EA dipeptide fragment, computed at the B3LYP/6-311++G(2*df*,2*pd*) level of theory. Intramolecular hydrogen bonds are represented by dotted lines and the magnitudes are computed from natural bond order computations, presented in milielectrons, me−.

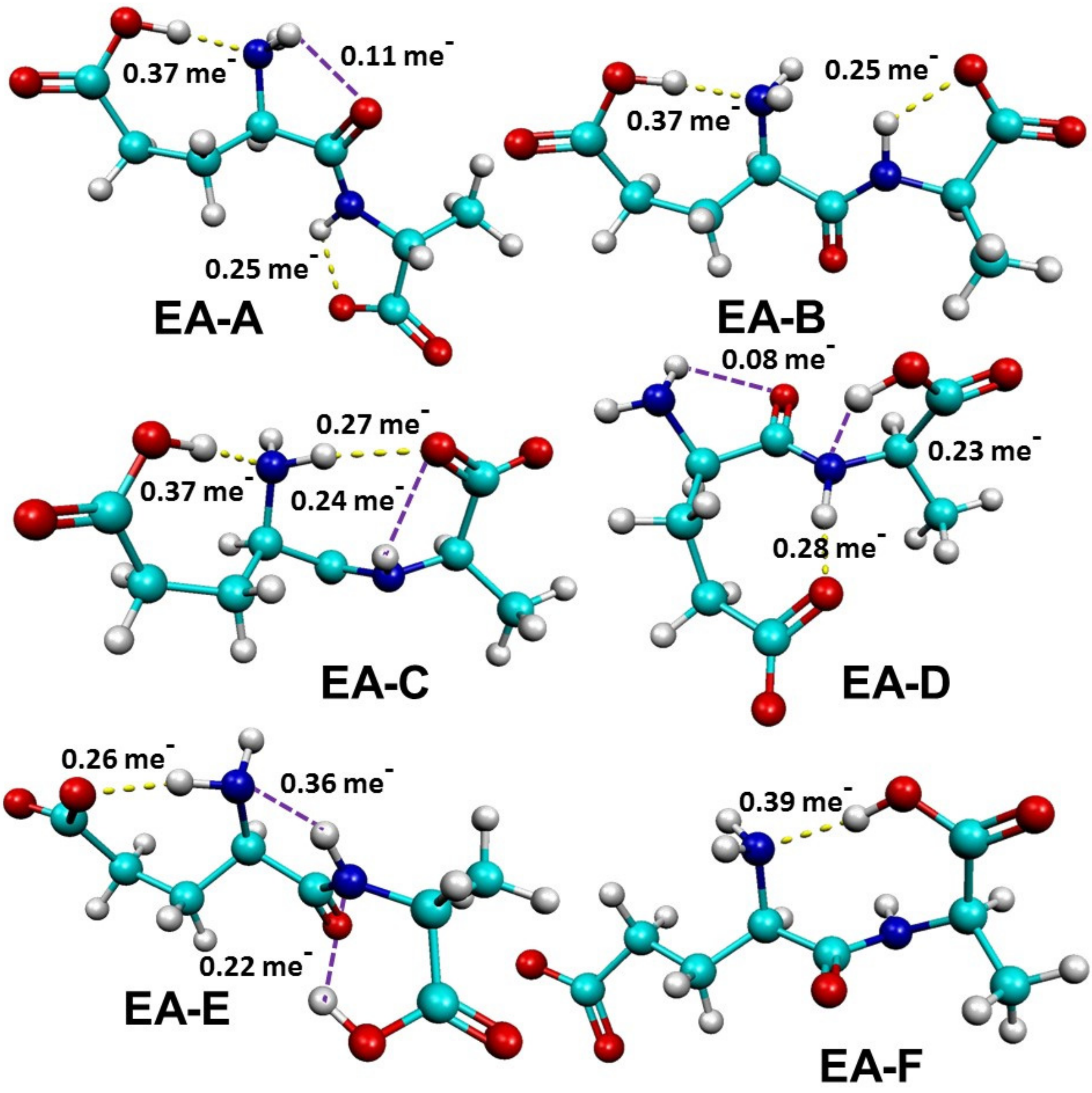

Table S3: Relative electronic energies of the EA dipeptide fragment, computed at the B3LYP/6-311++G(2*df*,2*pd*) level of theory. The magnitudes of the intramolecular hydrogen bonds are computed from natural bond order computations and are presented in milielectrons, me−. q_T represents the total charge transferred via intramolecular hydrogen bonding.

	ΔE	N_i	HBs	αNH_3^+-H bond, Δq, me^-	αCOO^--H bond, Δq, me^-	Other H bond, Δq, me^-	qT	Amide I v(C=O) Band Position, cm^{-1}	αCOO^- Terminal v(C=O) Band Position, cm^{-1}
**EA-A**	0.00	0.970975	3	0.37	0.25	0.11	0.73	1651	1642
**EA-B**	2.13	0.026362	2	0.37	0.25	---	0.62	1649	1642
**EA-C**	3.49	0.002656	3	0.37	0.27	0.24	0.88	1679	1629
**EA-D**	7.08	6.24E-06	3	0.08	0.23	0.28	0.59	1653	1759
**EA-E**	12.27	9.63E-10	3	0.26	0.22	0.36	0.84	1680	1767
**EA-F**	24.01	2.35E-18	1	0.39	---	---	0.39	1712	1734

Figure S4: Molecular geometries of the EGE tripeptide fragment, computed at the B3LYP/6-311++G(2*df*,2*pd*) level of theory. Intramolecular hydrogen bonds are represented by dotted lines and the magnitudes are computed from natural bond order computations, presented in milielectrons, me−.

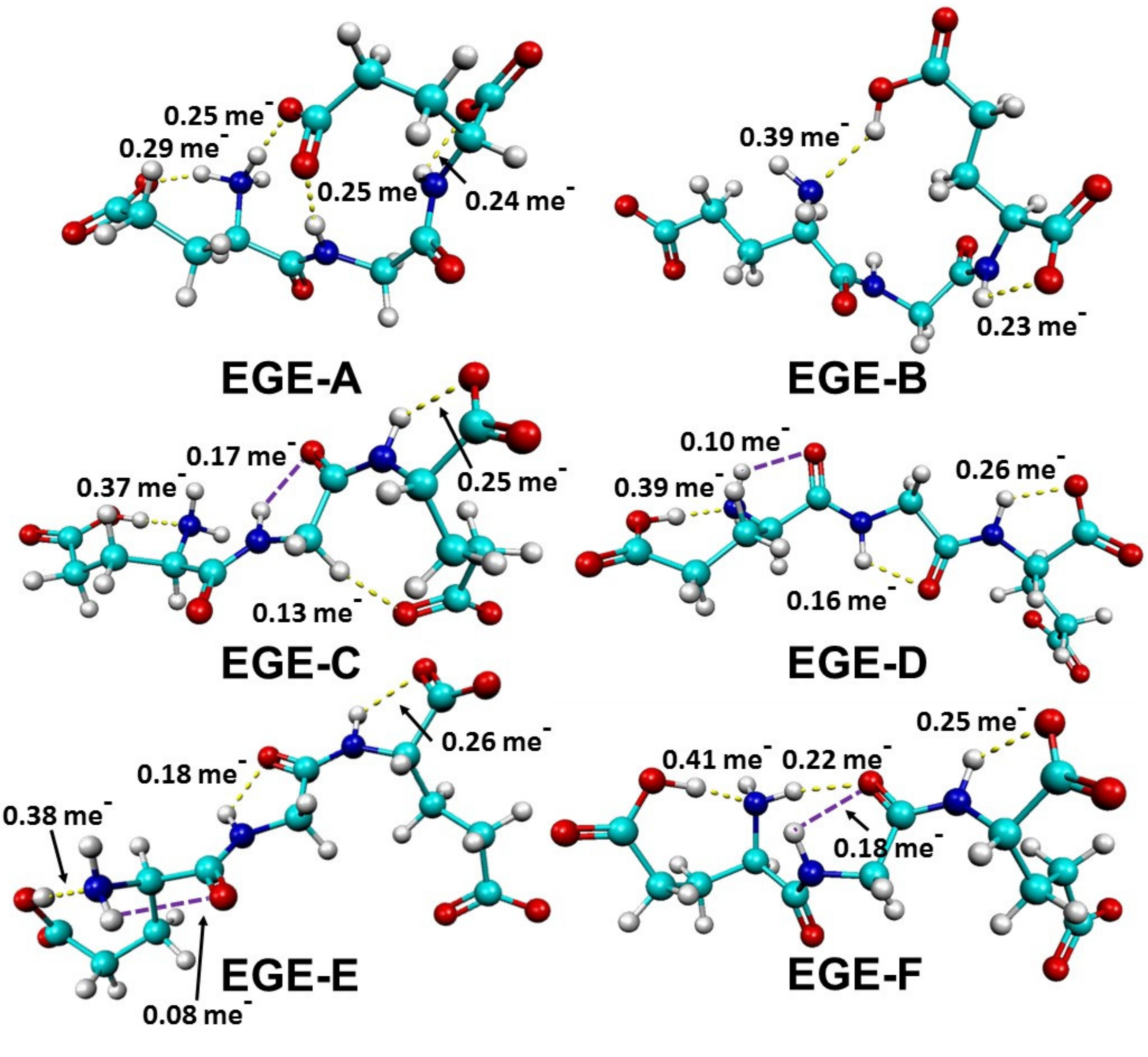

Table S4: Relative electronic energies of the EGE tripeptide fragment, computed at the B3LYP/6-311++G(2*df*,2*pd*) level of theory. The magnitudes of the intramolecular hydrogen bonds are computed from natural bond order computations and are presented in milielectrons, me−. q_T represents the total charge transferred via intramolecular hydrogen bonding.

	ΔE	N_i	HBs	αNH_3^+-H bond, Δq, me-	αCOO^--H bond, Δq, me-	Other H bond, Δq, me-	Other H bond, Δq, me-	qT	Amide I v(C=O) Band, cm^{-1}	αCOO^- Terminal v(C=O), cm^{-1}
**EGE-A**	0.00	1.000000	4	0.29	0.24	0.25	0.25	1.03	1617	1641
**EGE-B**	10.13	3.70E-08	2	0.39	0.23	---	---	0.62	1626	1685
**EGE-C**	16.59	6.73E-13	4	0.37	0.25	0.17	0.13	0.92	1664	1629
**EGE-D**	17.14	2.67E-13	4	0.39	0.26	0.10	0.16	0.91	1655	1627
**EGE-E**	19.73	3.33E-15	4	0.38	0.26	0.08	0.18	0.90	1667	1623
**EGE-F**	24.09	2.10E-18	4	0.41	0.25	0.22	0.18	1.06	1702	1631

Figure S5: Molecular geometries of the EDE tripeptide fragment, computed at the B3LYP/6-311++G(2*df*,2*pd*) level of theory. Intramolecular hydrogen bonds are represented by dotted lines and the magnitudes are computed from natural bond order computations, presented in milielectrons, me−.

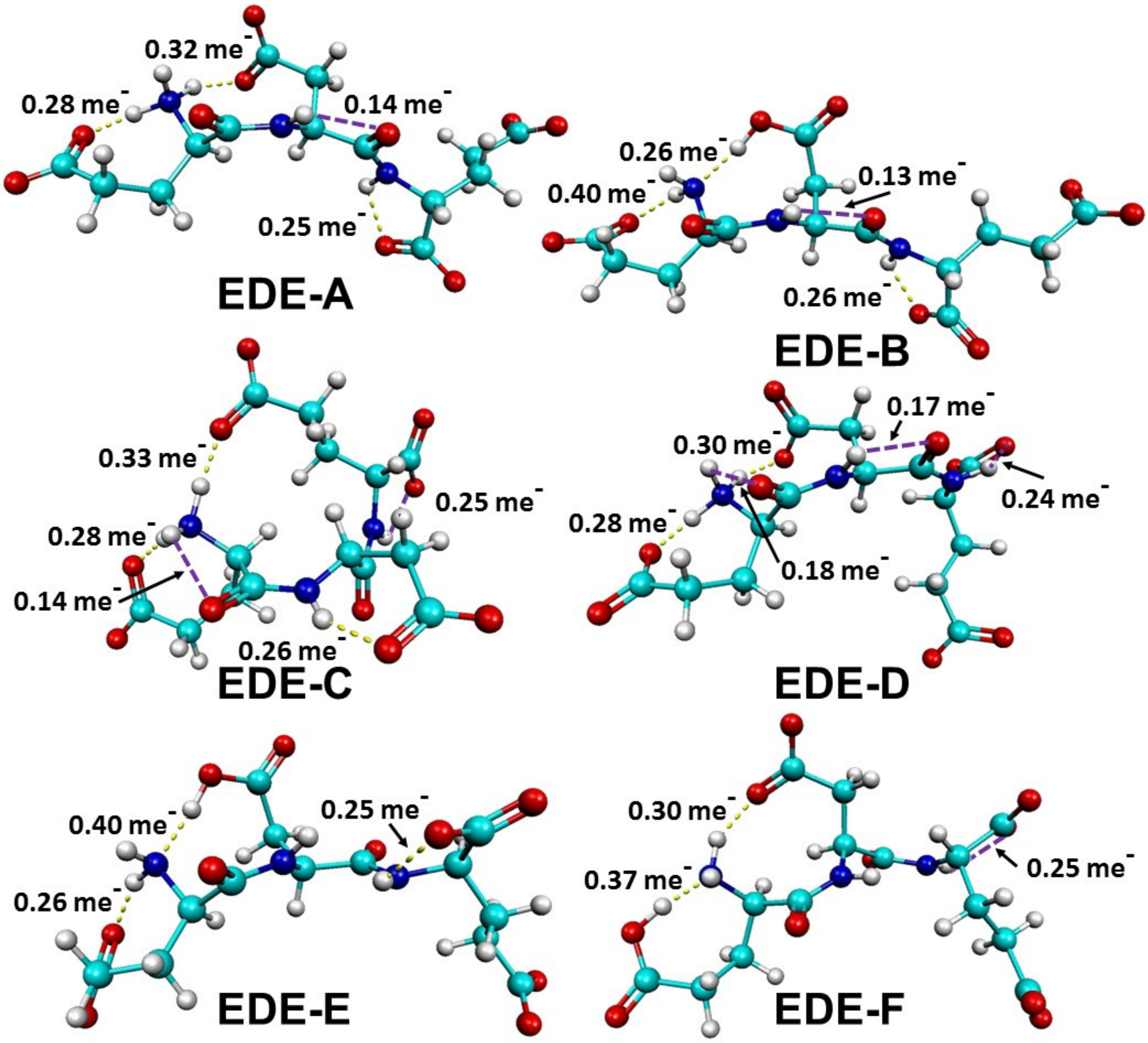

Table S5: Relative electronic energies of the EDE tripeptide fragment, computed at the B3LYP/6-311++G(2*df*,2*pd*) level of theory. The magnitudes of the intramolecular hydrogen bonds are computed from natural bond order computations and are presented in milielectrons, me−. q_T represents the total charge transferred via intramolecular hydrogen bonding.

	ΔE	N_i	HBs	αNH_3^+-H bond, Δq, me^-	αCOO^--H bond, Δq, me^-	Other H bond, Δq, me^-	Other H bond, Δq, me^-	Other H bond, Δq, me^-	qT	Amide I ν(C=O) Band, cm^{-1}	αCOO^- Terminal ν(C=O) Band, cm^{-1}
**EDE-A**	0.00	0.997126	4	0.28	0.25	0.32	0.14		0.99	1634	1609
**EDE-B**	3.64	0.002141	4	0.4	0.26	0.26	0.13		1.05	1626	1613
**EDE-C**	4.46	0.000531	5	0.33	0.25	0.28	0.26	0.14	1.26	1638	1611
**EDE-D**	5.03	0.000202	5	0.3	0.18	0.28	0.24	0.17	1.17	1637	1605
**EDE-E**	7.89	1.62E-06	3	0.4	0.25	0.26	---	---	0.91	1659	1606
**EDE-F**	12.00	1.57E-09	3	0.37	0.25	0.3	---		0.92	1635	1619

Figure S6: Molecular geometries of the DEA tripeptide fragment, computed at the B3LYP/6-311++G(2*df*,2*pd*) level of theory. Intramolecular hydrogen bonds are represented by dotted lines and the magnitudes are computed from natural bond order computations, presented in milielectrons, me−.

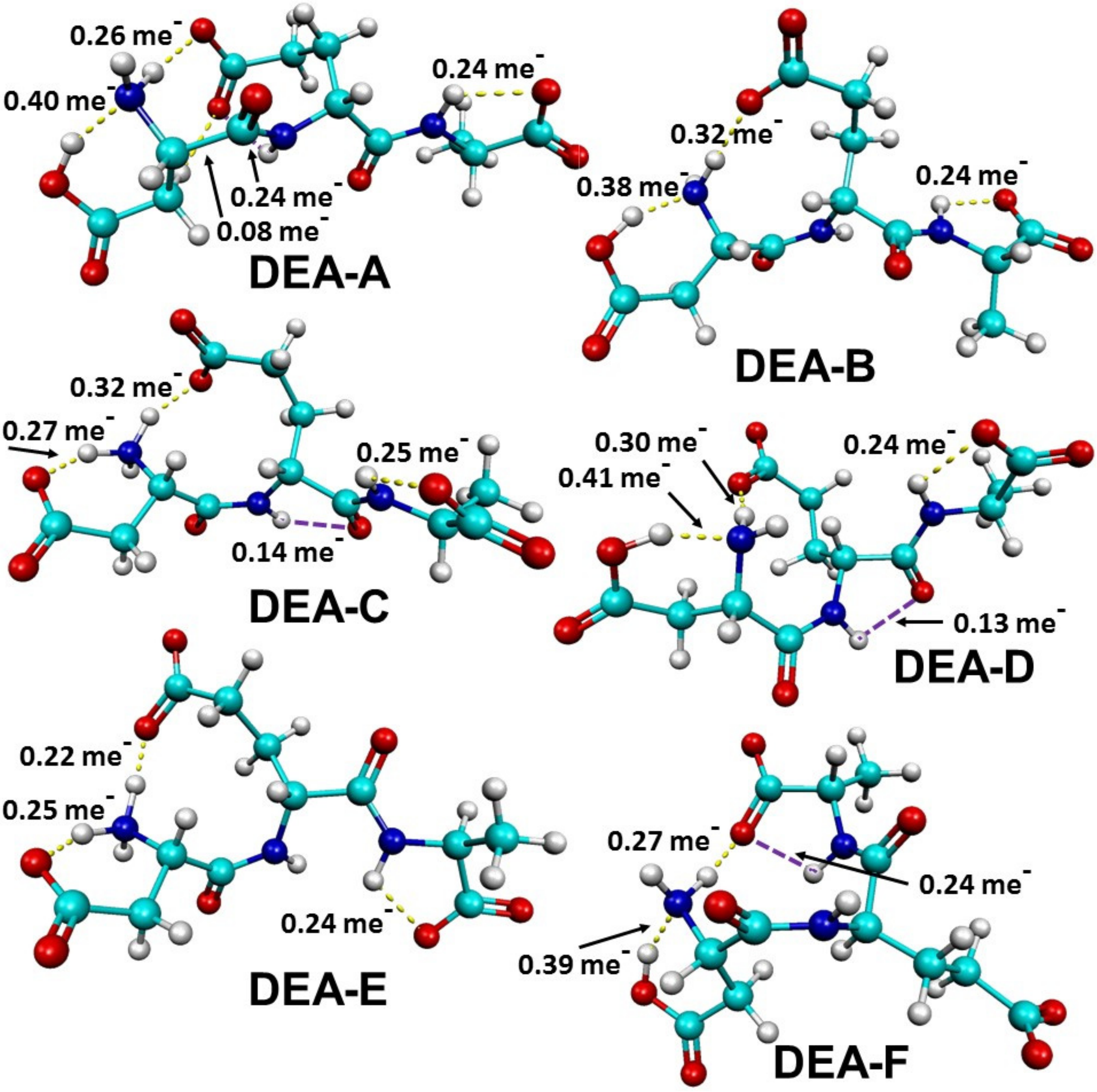

Table S6: Relative electronic energies of the DEA tripeptide fragment, computed at the B3LYP/6-311++G(2*df*,2*pd*) level of theory. The magnitudes of the intramolecular hydrogen bonds are computed from natural bond order computations and are presented in milielectrons, me–. q_T represents the total charge transferred via intramolecular hydrogen bonding.

	**ΔE**	**N_i**	**HBs**	**αNH_3^+-H bond, Δq, me^-**	**αCOO^--H bond, Δq, me^-**	**Other H bond, Δq, me^-**	**Other H bond, Δq, me^-**	**Other H bond, Δq, me^-**	**qT**	**Amide I ν(C=O) Band, cm^{-1}**	**αCOO^- Terminal ν(C=O) Band, cm^{-1}**
**DEA-A**	0.00	0.998636	5	0.4	0.24	0.26	0.08	0.24	1.22	1635	1618
**DEA-B**	4.16	0.000879	3	0.38	0.24	0.32			0.94	1643	1622
**DEA-C**	4.54	0.000469	4	0.32	0.25	0.27	0.14		0.98	1633	1628
**DEA-D**	6.60	1.43E-05	4	0.41	0.24	0.3	0.13		1.08	1614	1624
**DEA-E**	7.89	1.62E-06	3	0.25	0.24	0.22			0.71	1664	1626
**DEA-F**	13.89	6.39E-11	3	0.39	0.27	0.24			0.9	1622	1617

Figure S7: Molecular geometries of the EGED tetrapeptide fragment, computed at the B3LYP/6-311++G(2*df*,2*pd*) level of theory. Intramolecular hydrogen bonds are represented by dotted lines and the magnitudes are computed from natural bond order computations, presented in milielectrons, me−.

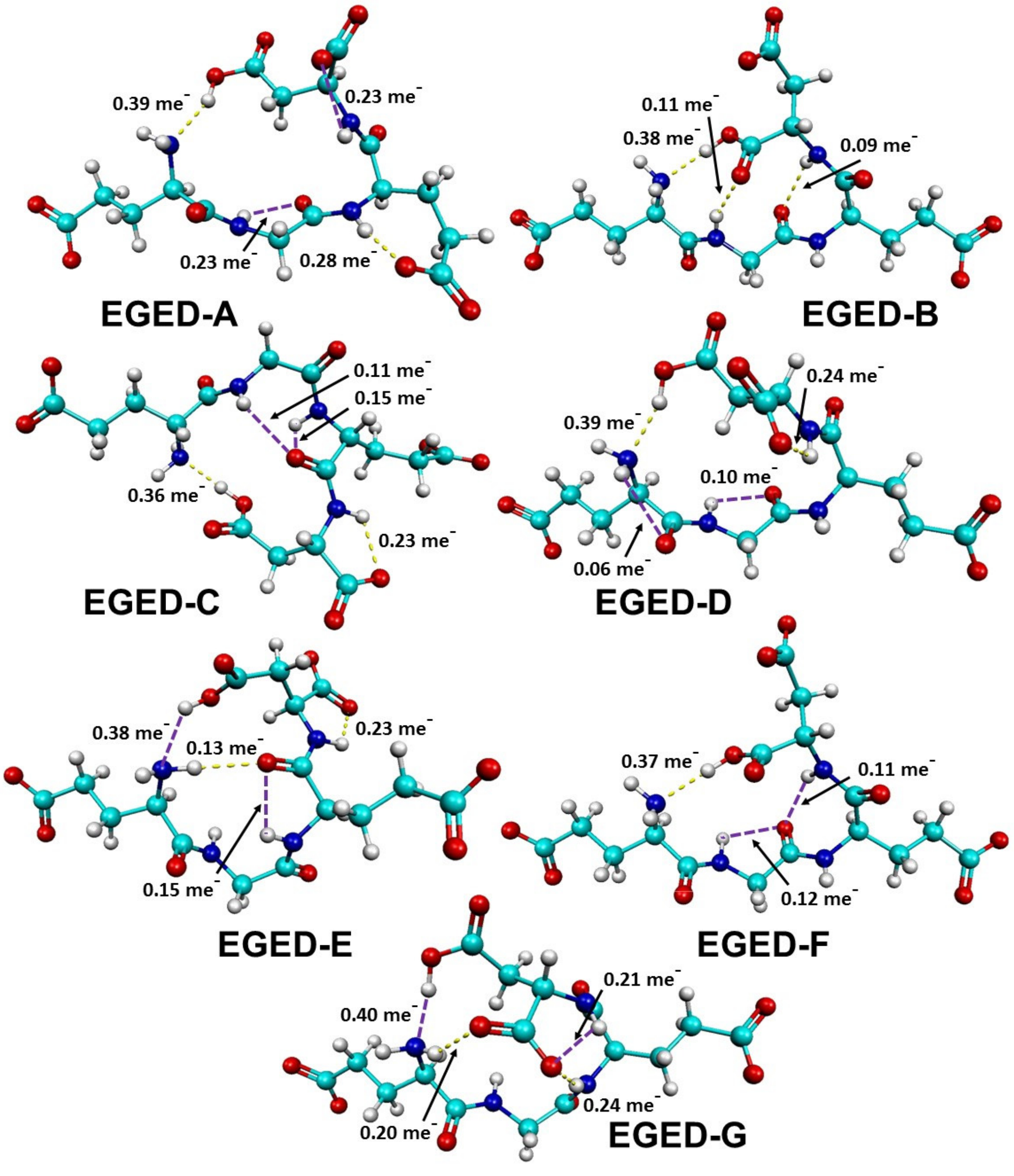

Table S7: Relative electronic energies of the EGED tetrapeptide fragment, computed at the B3LYP/6-311++G(2*df*,2*pd*) level of theory. The magnitudes of the intramolecular hydrogen bonds are computed from natural bond order computations and are presented in milielectrons, me−. q_T represents the total charge transferred via intramolecular hydrogen bonding.

	**ΔE**	**N$_i$**	**HBs**	**αNH_3^+-H bond, Δq, me$^-$**	**αCOO⁻-H bond, Δq, me$^-$**	**Other H bond, Δq, me$^-$**	**Other H bond, Δq, me$^-$**	**qT**	**Amide I v(C=O) Band, cm^{-1}**	**αCOO⁻ Terminal v(C=O) Band, cm^{-1}**
**EGED-A**	0.00	0.988073	4	0.39	0.23	0.28	0.12	1.02	1676	1620
**EGED-B**	3.02	0.006044	3	0.38	0.11	0.09		0.58	1675	1650
**EGED-C**	3.44	0.002932	4	0.36	0.23	0.15	0.11	0.85	1661	1631
**EGED-D**	3.71	0.001874	4	0.39	0.24	0.06	0.1	0.79	1680	1633
**EGED-E**	4.29	0.000709	4	0.38	0.23	0.13	0.15	0.89	1669	1633
**EGED-F**	4.68	0.000366	2	0.37	---	0.11	0.12	0.6	1677	1727
**EGED-G**	8.21	9.34E-07	4	0.4	0.2	0.24	0.21	1.05	1678	1617

Figure S8: Molecular geometries of the EDEA tetrapeptide fragment, computed at the B3LYP/6-311++G(2*df*,2*pd*) level of theory. Intramolecular hydrogen bonds are represented by dotted lines and the magnitudes are computed from natural bond order computations, presented in milielectrons, me−.

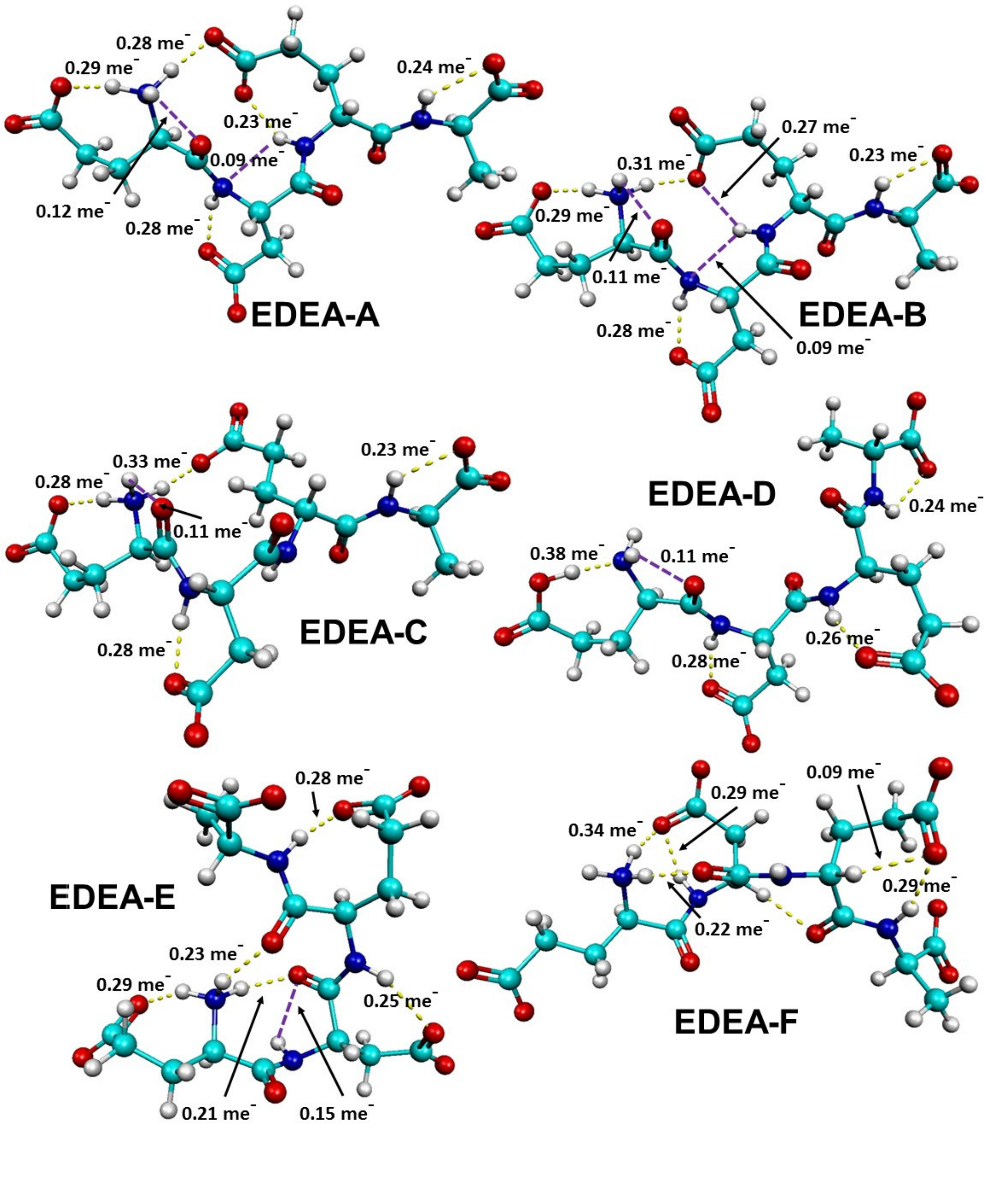

Table S8: Relative electronic energies of the EDEA tetrapeptide fragment, computed at the B3LYP/6-311++G(2*df*,2*pd*) level of theory. The magnitudes of the intramolecular hydrogen bonds are computed from natural bond order computations and are presented in milielectrons, me−. q_T represents the total charge transferred via intramolecular hydrogen bonding.

	ΔE	N_i	HBs	αNH_3^+- H bond, Δq, me^-	αCOO^-- H bond, Δq, me^-	Other H bond, Δq, me^-	Other H bond, Δq, me^-	Other H bond, Δq, me^-	Other H bond, Δq, me^-	qT	Amide I ν(C=O) Band, cm^{-1}	αCOO^- Terminal ν(C=O) Band, cm^{-1}
**EDEA-A**	0.00	0.929480	7	0.28	0.24	0.29	0.28	0.23	0.12	1.44	1639	1604
**EDEA-B**	1.53	0.070518	6	0.31	0.23	0.29	0.27	0.28	0.11	1.49	1652	1606
**EDEA-C**	7.87	1.55E-06	5	0.33	0.23	0.28	0.28	0.11		1.23	1642	1612
**EDEA-D**	16.11	1.41E-12	5	0.38	0.24	0.11	0.28	0.26		1.27	1671	1601
**EDEA-E**	34.60	3.84E-26	6	0.29	0.28	0.23	0.21	0.25	0.15	1.41	1708	1615
**EDEA-F**	47.14	2.40E-35	5	0.34	---	0.22	0.29	0.29	0.09	1.23	1739	1609

Figure S9: Molecular geometries of the EGEDE pentapeptide fragment, computed at the B3LYP/6-311++G(2*df*,2*pd*) level of theory. Intramolecular hydrogen bonds are represented by dotted lines and the magnitudes are computed from natural bond order computations, presented in milielectrons, me−.

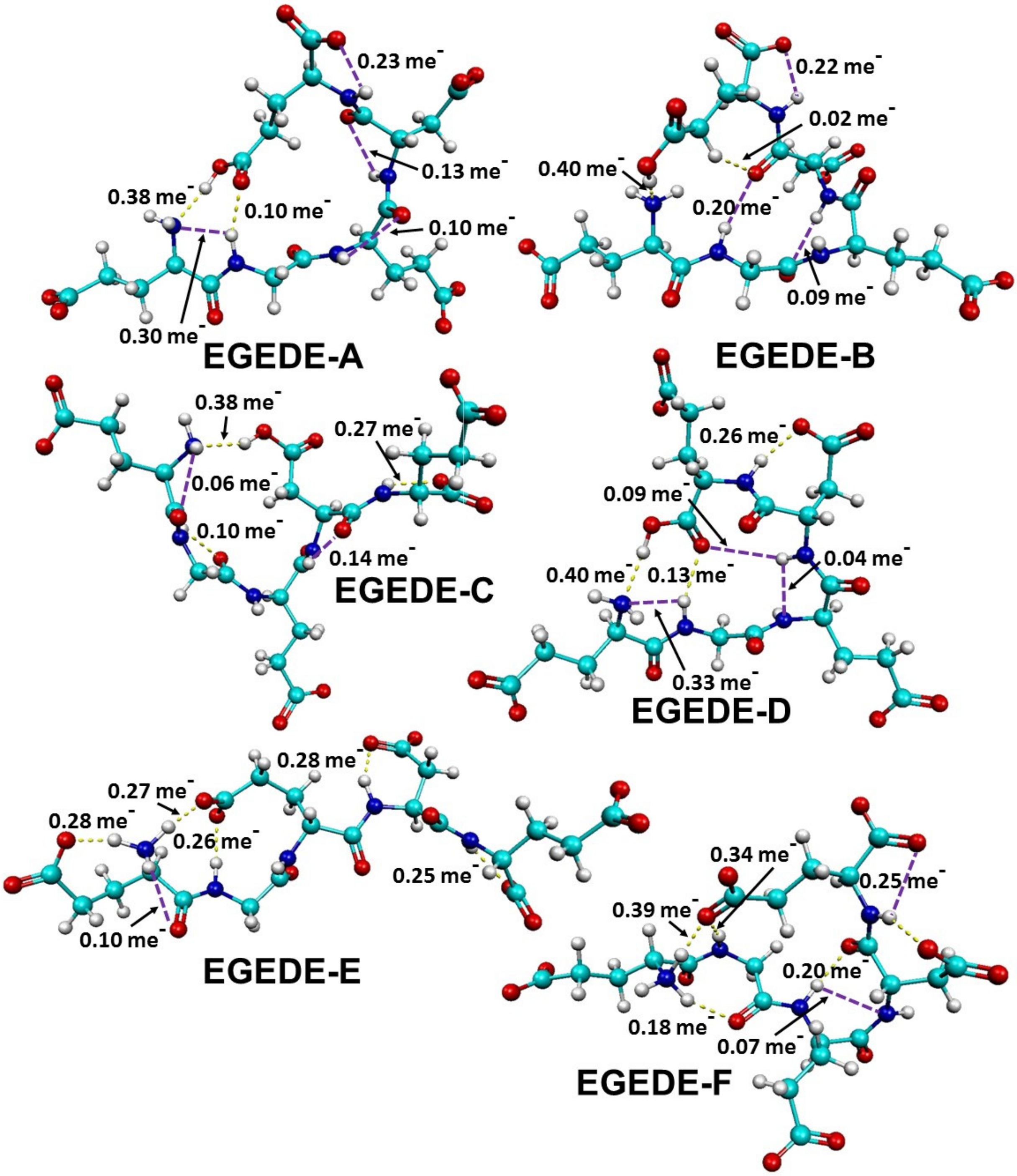

Table S9: Relative electronic energies of the EGEDE pentapeptide fragment, computed at the B3LYP/6-311++G(2*df*,2*pd*) level of theory. The magnitudes of the intramolecular hydrogen bonds are computed from natural bond order computations and are presented in milielectrons, me–. q_T represents the total charge transferred via intramolecular hydrogen bonding.

	**ΔE**	**N*i***	**HBs**	**αNH_3^+-H bond, Δq, me^-**	**αCOO^--H bond, Δq, me^-**	**Other H bond, Δq, me^-**	**Other H bond, Δq, me^-**	**Other H bond, Δq, me^-**	**Other H bond, Δq, me^-**	**qT**	**Amide I ν(C=O) Band, cm^{-1}**	**αCOO^- Terminal ν(C=O) Band, cm^{-1}**
**EGEDE-A**	0.00	0.999516	6	0.38	0.23	0.3	0.1	0.1	0.13	1.24	1650	1614
**EGEDE-B**	4.52	0.000483	5	0.4	0.22	0.2	0.02	0.09		0.93	1659	1625
**EGEDE-C**	8.65	4.51E-07	5	0.38	0.27	0.06	0.1	0.14		0.95	1682	1609
**EGEDE-D**	11.67	2.72E-09	6	0.4	0.26	0.33	0.13	0.09	0.04	1.25	1652	1698
**EGEDE-E**	11.70	2.62E-09	6	0.28	0.25	0.27	0.1	0.28	0.26	1.44	1658	1593
**EGEDE-F**	32.43	1.59E-24	6	0.39	0.25	0.34	0.18	0.07	0.2	1.43	1698	1605

Figure S10: Molecular geometries of the GEDEA pentapeptide fragment, computed at the B3LYP/6-311++G(2*df*,2*pd*) level of theory. Intramolecular hydrogen bonds are represented by dotted lines and the magnitudes are computed from natural bond order computations, presented in milielectrons, me−.

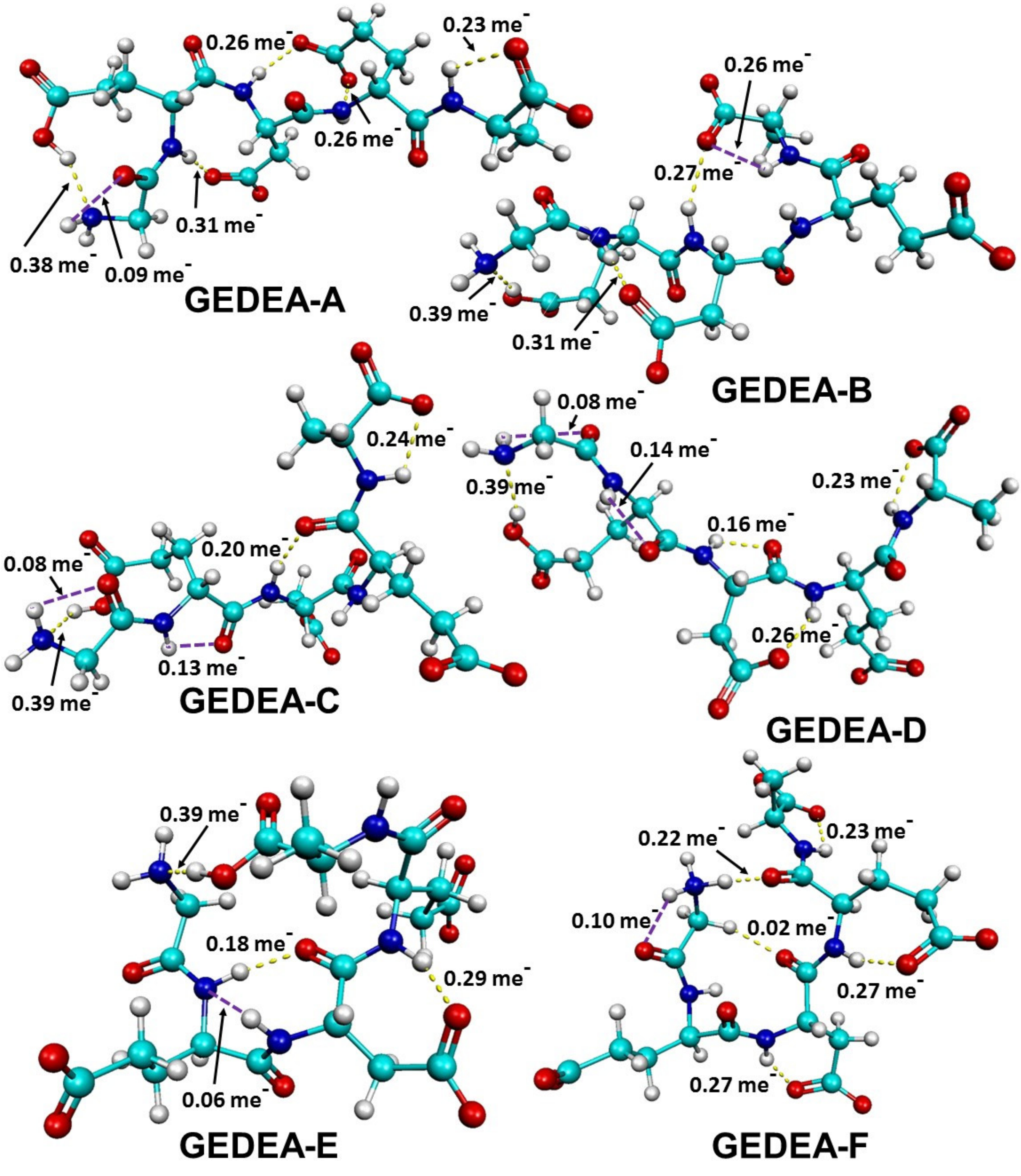

Table S10: Relative electronic energies of the GEDEA pentapeptide fragment, computed at the B3LYP/6-311++G(2*df*,2*pd*) level of theory. The magnitudes of the intramolecular hydrogen bonds are computed from natural bond order computations and are presented in milielectrons, me−. q_T represents the total charge transferred via intramolecular hydrogen bonding.

	**ΔE**	**N_i**	**HBs**	**αNH_3^+-H bond, Δq, me^-**	**αCOO⁻-H bond, Δq, me^-**	**Other H bond, Δq, me^-**	**Other H bond, Δq, me^-**	**Other H bond, Δq, me^-**	**Other H bond, Δq, me^-**	**qT**	**Amide I v(C=O) Band, cm^{-1}**	**αCOO⁻ Terminal v(C=O) Band, cm^{-1}**
**GEDEA-A**	0.00	0.999954	6	0.38	0.23	0.31	0.26	0.26	0.09	1.53	1654	1607
**GEDEA-B**	5.92	4.55E-05	4	0.39	0.27	0.26	0.31			1.23	1661	1610
**GEDEA-C**	11.41	4.23E-09	5	0.39	0.24	0.08	0.2	0.13		1.04	1673	1618
**GEDEA-D**	11.47	3.86E-09	6	0.39	0.23	0.08	0.14	0.16	0.26	1.26	1672	1605
**GEDEA-E**	21.81	9.9E-17	4	0.39	---	0.18	0.06	0.29		0.92	1668	1713
**GEDEA-F**	27.11	1.28E-20	6	0.22	0.23	0.1	0.27	0.27	0.02	1.11	1706	1636

Figure S11: Molecular geometries of the EGEDEA hexapeptide fragment, computed at the B3LYP/6-311++G(2*df*,2*pd*) level of theory. Intramolecular hydrogen bonds are represented by dotted lines and the magnitudes are computed from natural bond order computations, presented in milielectrons, me−.

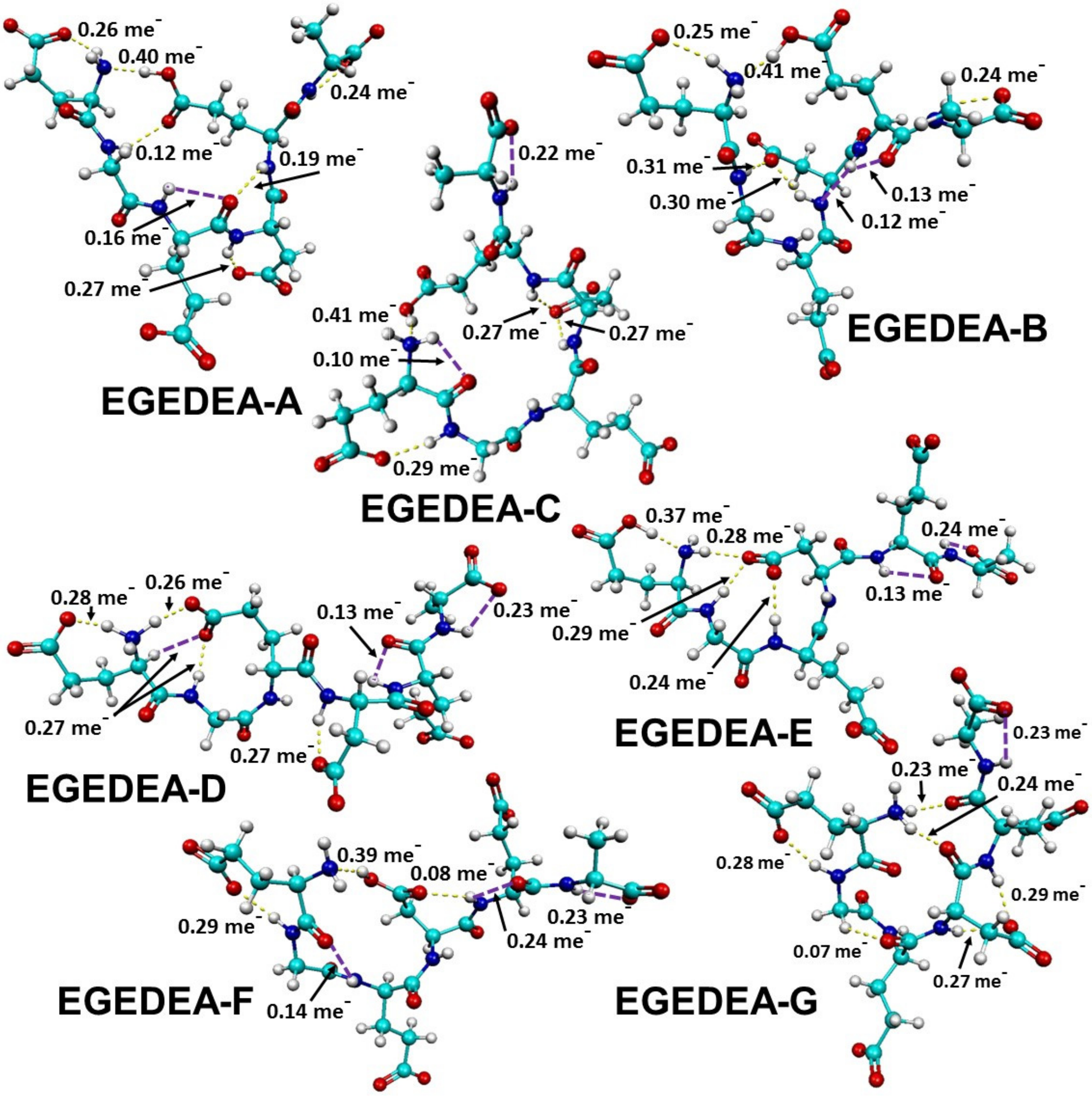

Table S11: Relative electronic energies of the EGEDEA hexaapeptide fragment, computed at the B3LYP/6-311++G(2*df*,2*pd*) level of theory. The magnitudes of the intramolecular hydrogen bonds are computed from natural bond order computations and are presented in milielectrons, me−. q_T represents the total charge transferred via intramolecular hydrogen bonding.

	ΔE	N_i	HBs	αNH_3^+-H bond, Δq, me^-	αCOO^--H bond, Δq, me^-	Other H bond, Δq, me^-	Other H bond, Δq, me^-	Other H bond, Δq, me^-	Other H bond, Δq, me^-	qT	Amide I v(C=O) Band, cm^{-1}	αCOO^- Terminal v(C=O) Band, cm^{-1}
**EGEDEA-A**	0.00	0.919794	7	0.4	0.24	0.26	0.27	0.19	0.16	1.64	1658	1610
**EGEDEA-B**	1.44	0.080206	7	0.41	0.24	0.25	0.31	0.3	0.12	1.76	1657	1611
**EGEDEA-C**	11.19	5.7E-09	6	0.4	0.22	0.27	0.27	0.29	0.1	1.55	1673	1604
**EGEDEA-D**	11.35	4.34E-09	6	0.28	0.23	0.26	0.27	0.27	0.13	1.44	1665	1602
**EGEDEA-E**	11.73	2.29E-09	6	0.37	0.24	0.28	0.29	0.24	0.13	1.55	1653	1604
**EGEDEA-F**	17.75	8.72E-14	6	0.39	0.23	0.29	0.14	0.24	0.08	1.37	1665	1606
**EGEDEA-G**	38.39	6.29E-29	7	0.24	0.23	0.23	0.28	0.07	0.27	1.61	1696	1616

Figure S12: Comparison of the simulated Raman spectra of the energetic minimum of the EG dipeptide computed with a variety of methods- B3LYP, M06-2X, PBEPBE, and MP2- to experiment, computed with the 6-311++G(2*df*,2*pd*) basis set and presented in cm^{-1}.

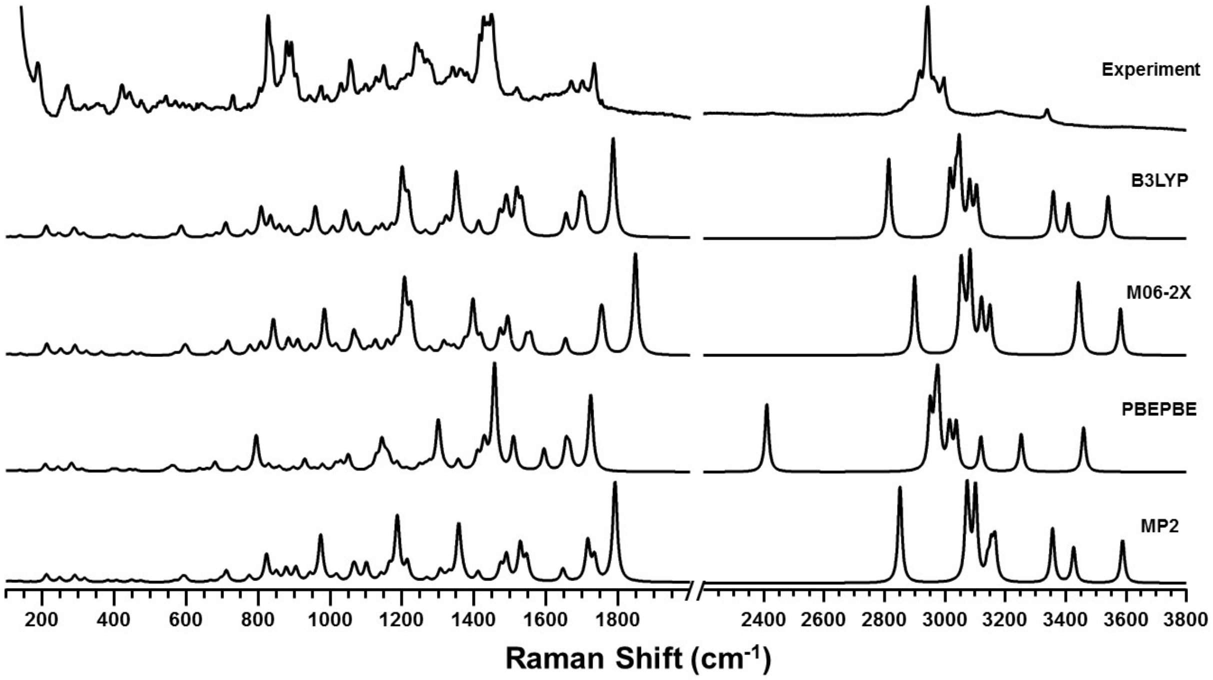

Figure S13: Comparison of the simulated Raman spectra of each energetic minimum of the EG dipeptide to experiment, computed at the B3LYP/6-311++G(2*df*,2*pd*) level of theory and presented in cm^{-1}.

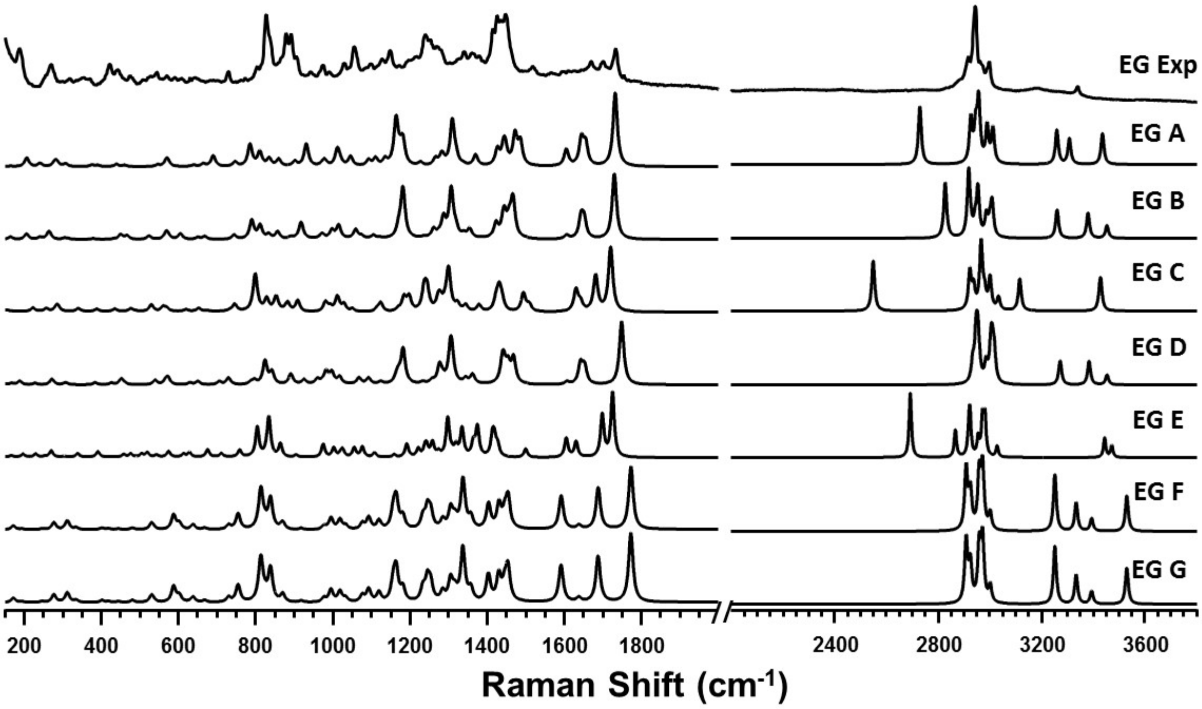

Figure S14: Comparison of the simulated Raman spectra of each energetic minimum of the ED dipeptide to experiment, computed at the B3LYP/6-311++G(2*df*,2*pd*) level of theory and presented in cm^{-1}.

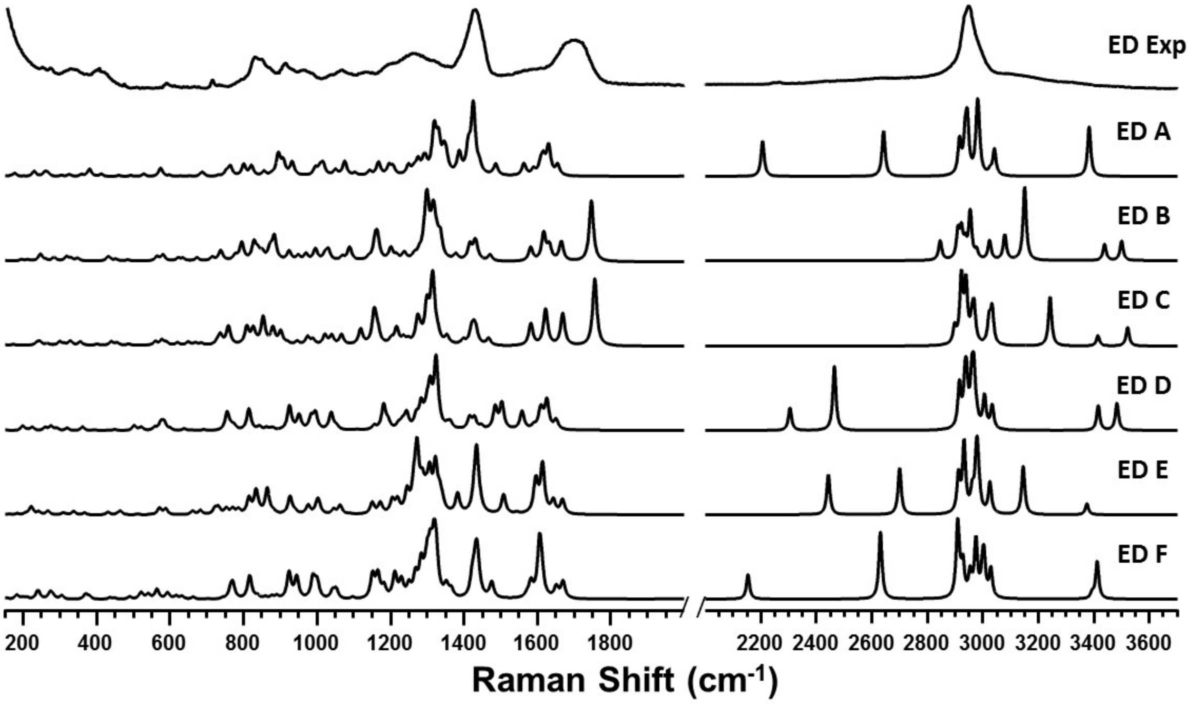

Figure S15: Comparison of the simulated Raman spectra of each energetic minimum of the EA dipeptide to experiment, computed at the B3LYP/6-311++G(2*df*,2*pd*) level of theory and presented in cm^{-1}.

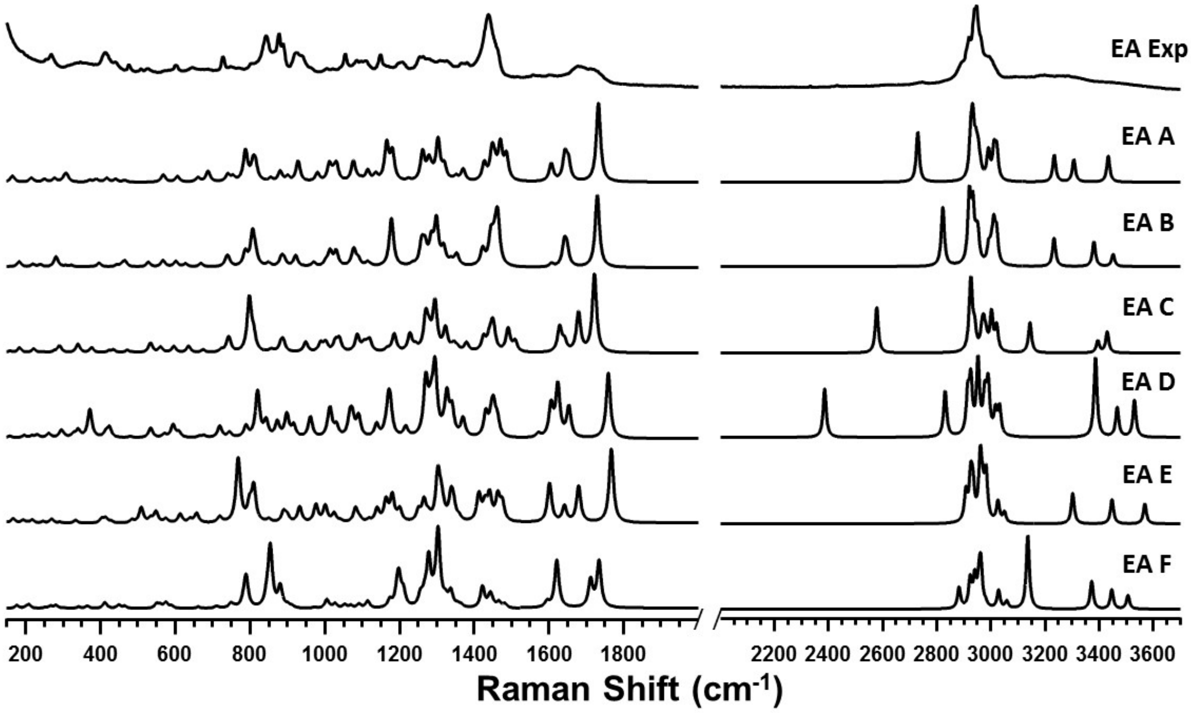

Figure S16: Comparison of the simulated Raman spectra of each energetic minimum of the EGE tripeptide to the Boltzmann summed simulated spectrum, computed at the B3LYP/6-311++G(2*df*,2*pd*) level of theory and presented in cm^{-1}.

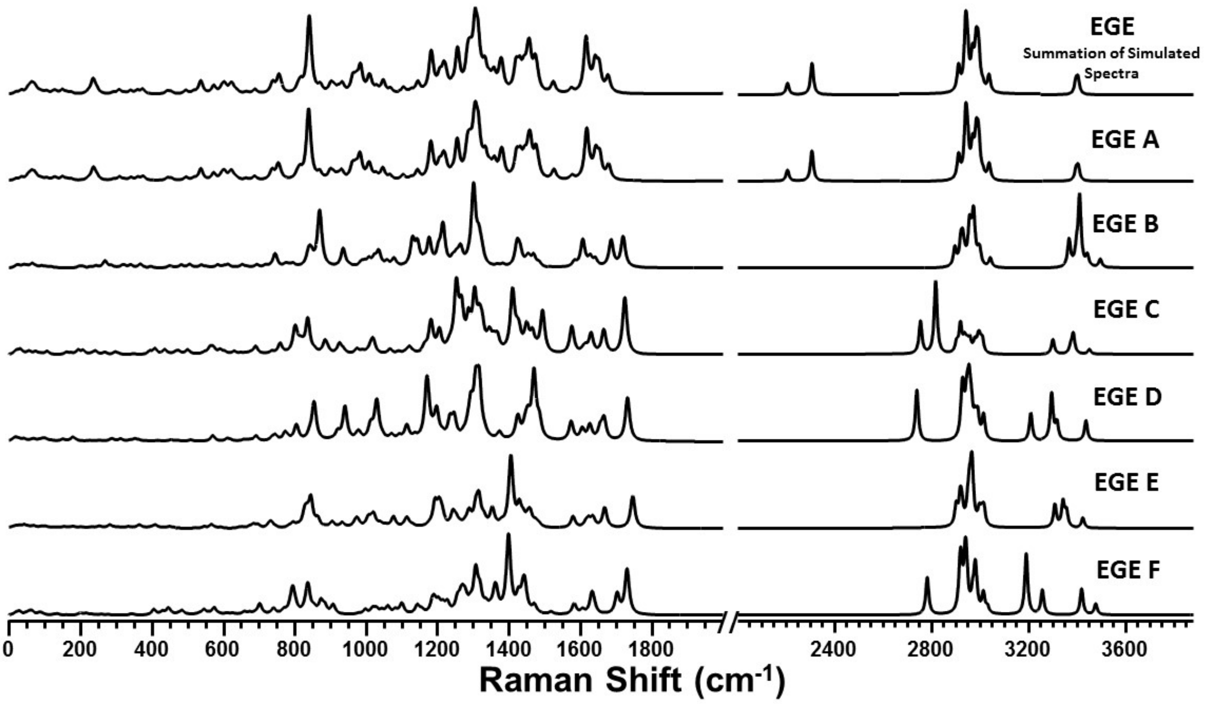

Figure S17: Comparison of the simulated Raman spectra of each energetic minimum of the EDE tripeptide to the Boltzmann summed simulated spectrum, computed at the B3LYP/6-311++G(2*df*,2*pd*) level of theory and presented in cm^{-1}.

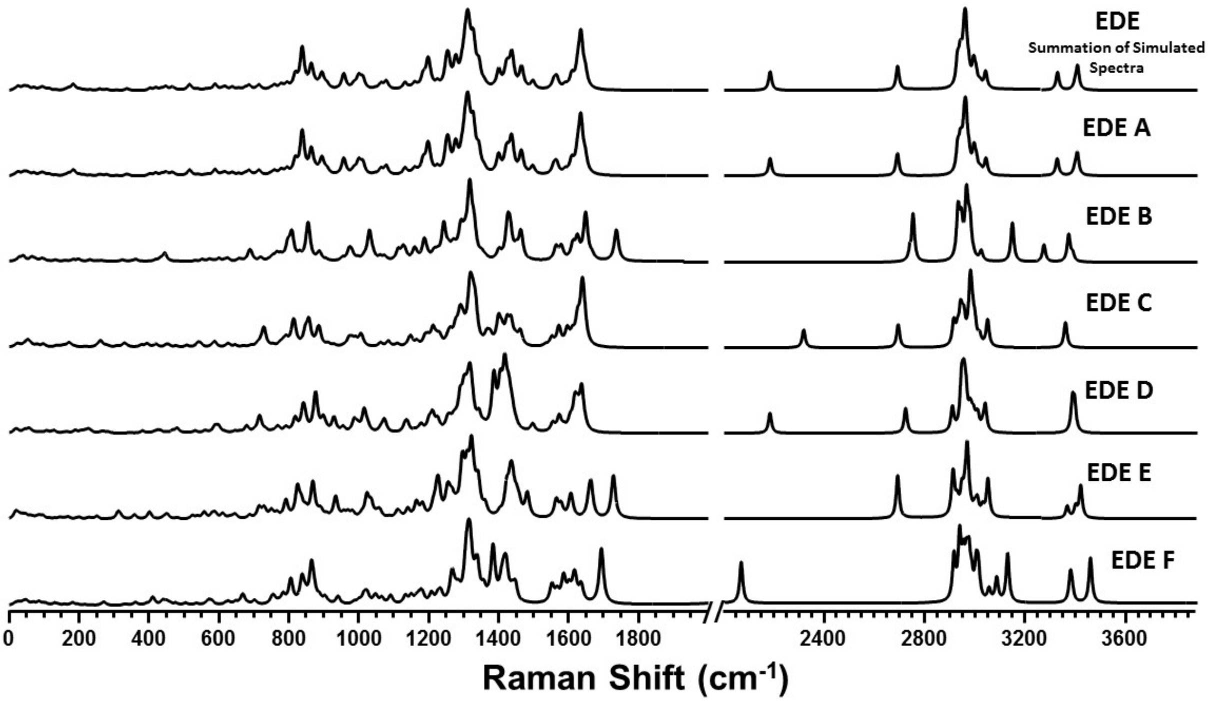

Figure S18: Comparison of the simulated Raman spectra of each energetic minimum of the DEA tripeptide to the Boltzmann summed simulated spectrum, computed at the B3LYP/6-311++G(2*df*,2*pd*) level of theory and presented in cm^{-1}.

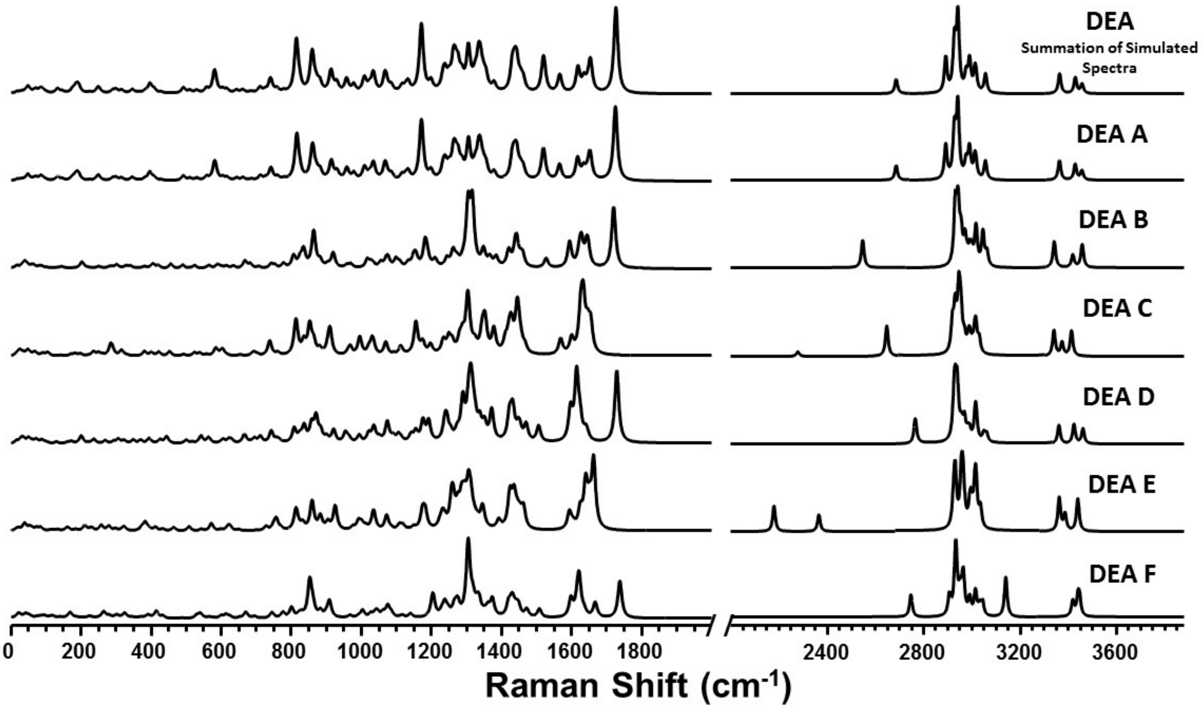

Figure S19: Comparison of the simulated Raman spectra of each energetic minimum of the EGED tetrapeptide to experiment, computed at the B3LYP/6-311++G(2*df*,2*pd*) level of theory and presented in cm^{-1}.

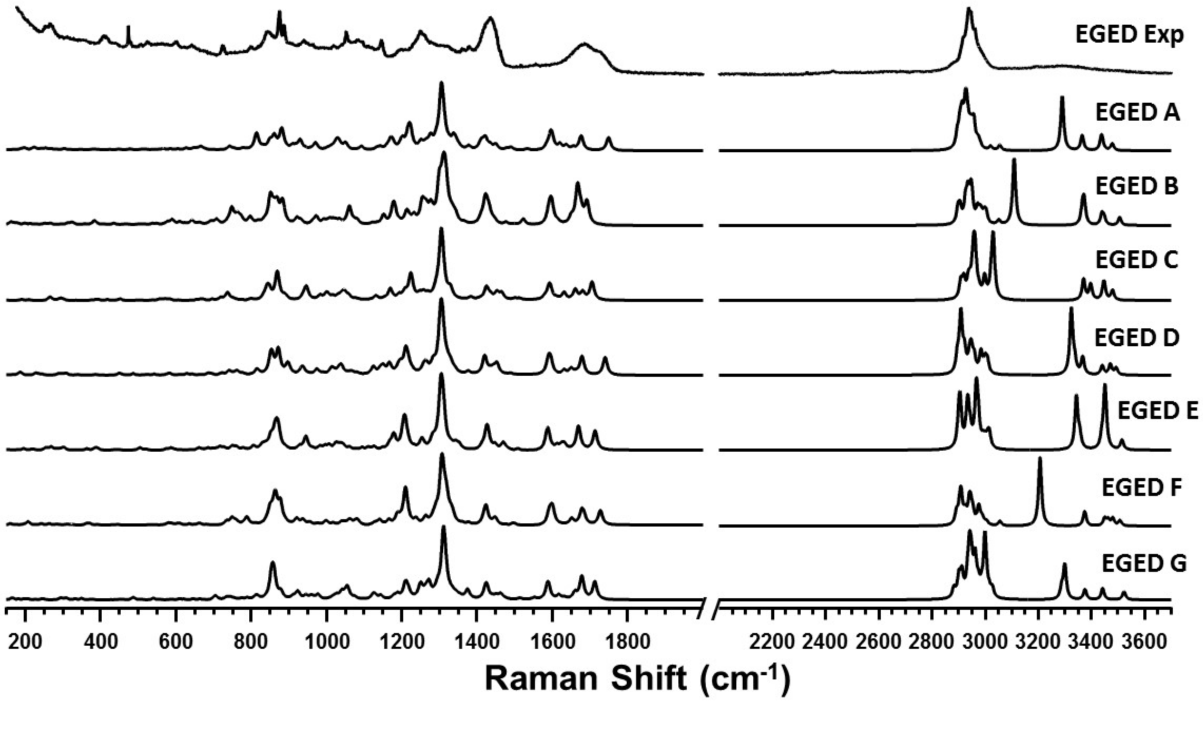

Figure S20: Comparison of the simulated Raman spectra of each energetic minimum of the EDEA tetrapeptide to experiment, computed at the B3LYP/6-311++G(2*df*,2*pd*) level of theory and presented in cm^{-1}.

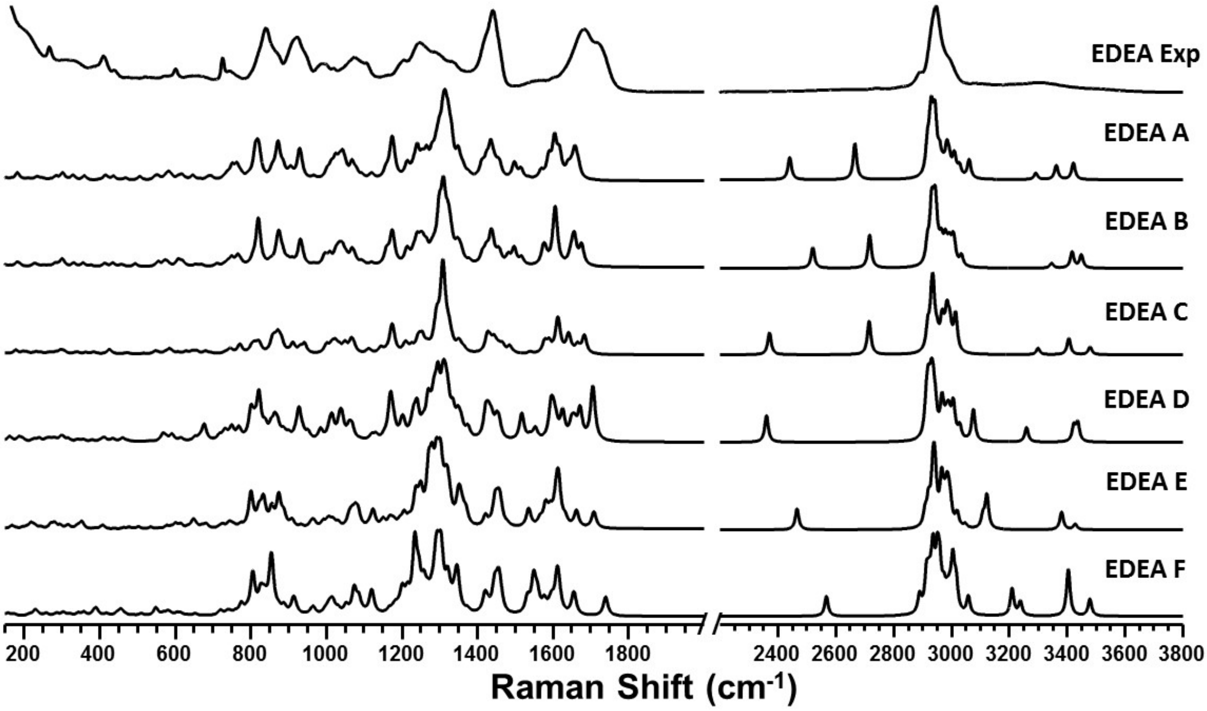

Figure S21: Comparison of the simulated Raman spectra of each energetic minimum of the EGEDE pentapeptide to the Boltzmann summed simulated spectrum, computed at the B3LYP/6-311++G(2*df*,2*pd*) level of theory and presented in cm^{-1}.

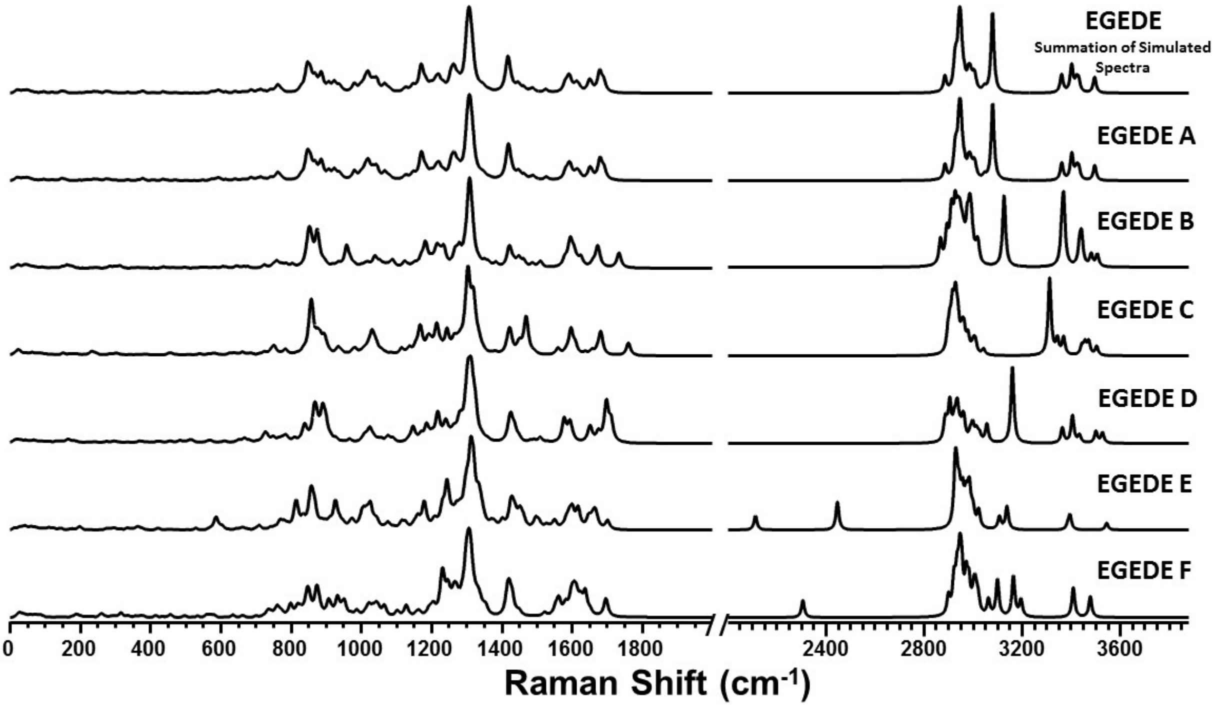

Figure S22: Comparison of the simulated Raman spectra of each energetic minimum of the GEDEA pentapeptide to the Boltzmann summed simulated spectrum, computed at the B3LYP/6-311++G(2*df*,2*pd*) level of theory and presented in cm^{-1}.

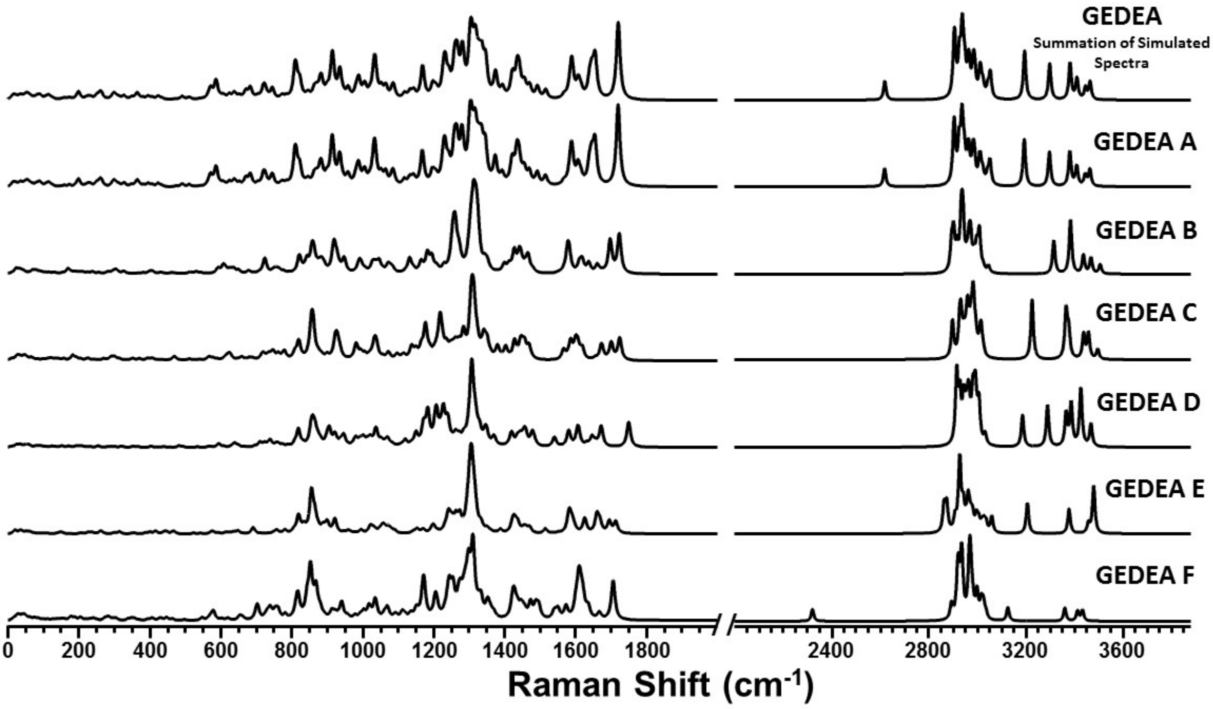

Figure S23: Comparison of the simulated Raman spectra of each energetic minimum of the EGEDEA hexapeptide to experiment, computed at the B3LYP/6-311++G(2*df*,2*pd*) level of theory and presented in cm^{-1}.

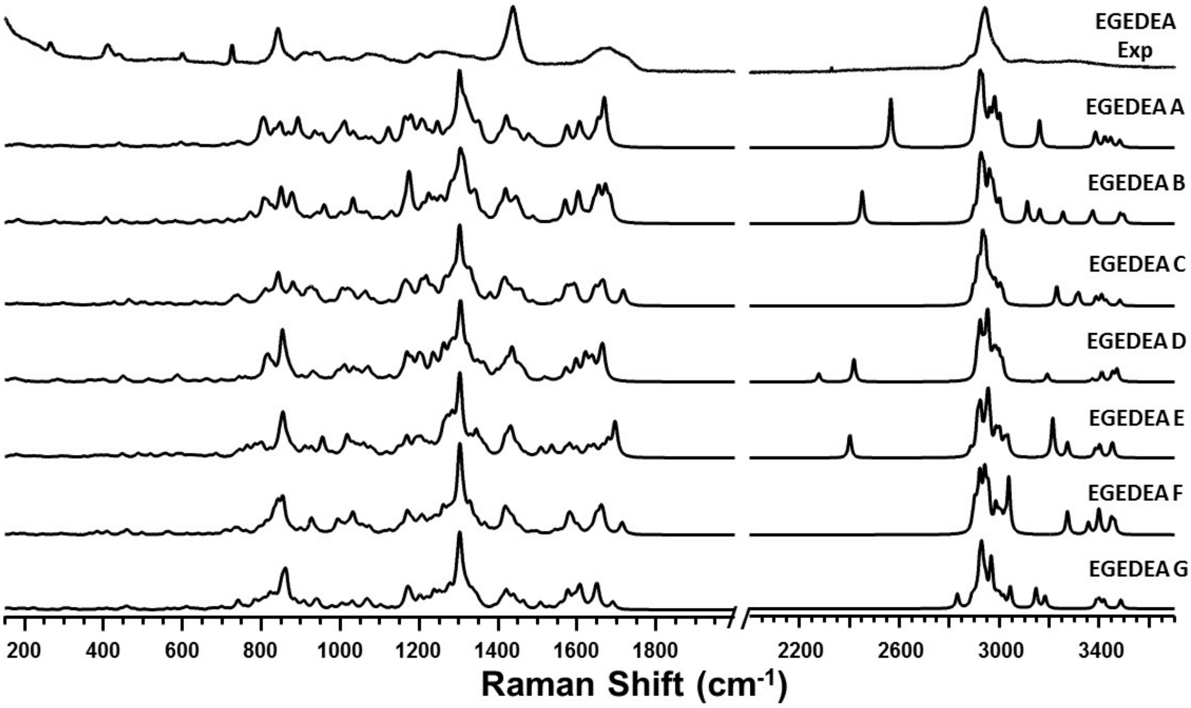

Figure S24: Ramachandran plot of the computed φ and ψ bond angles for each of the energetic minima of the EG dipeptide (Figure S1), presented in degrees (^{O}). The Boltzmann sums of the weighted values are also presented.

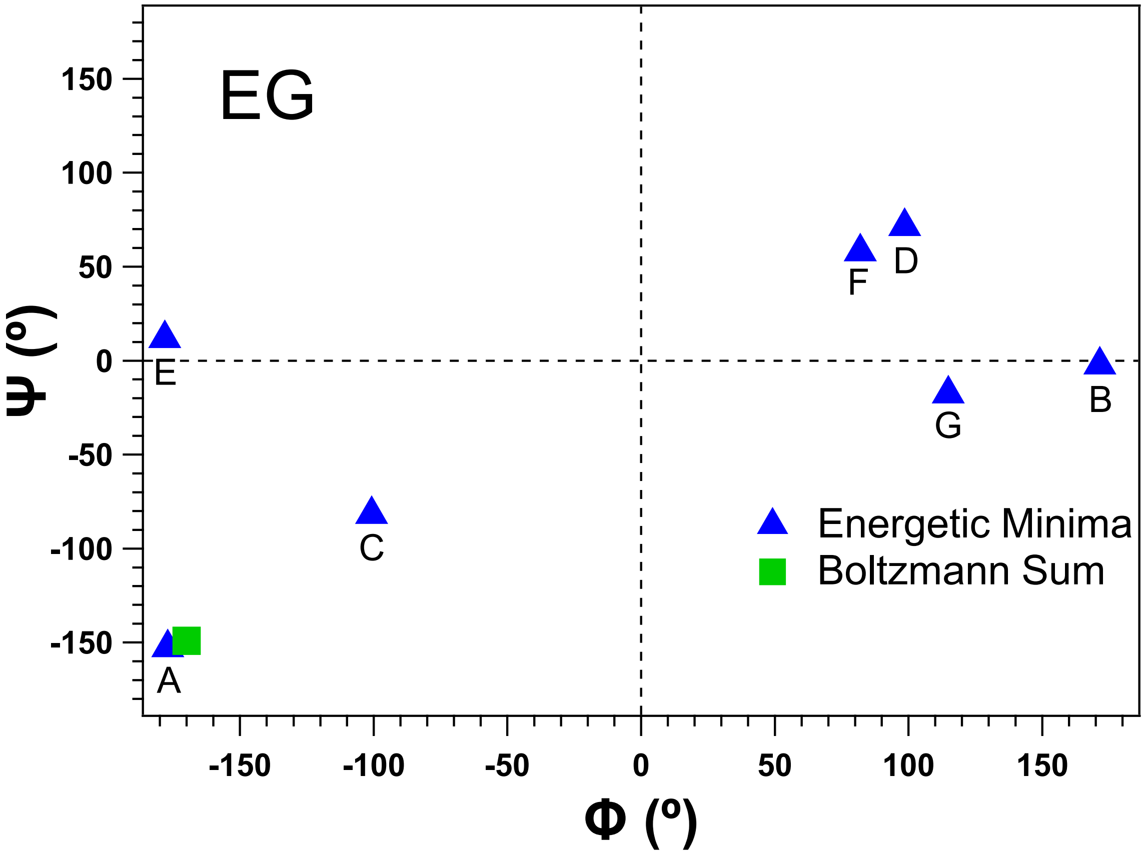

Figure S25: Ramachandran plot of the computed φ and ψ bond angles for each of the energetic minima of the ED dipeptide (Figure S2), presented in degrees (^{O}). The Boltzmann sums of the weighted values are also presented.

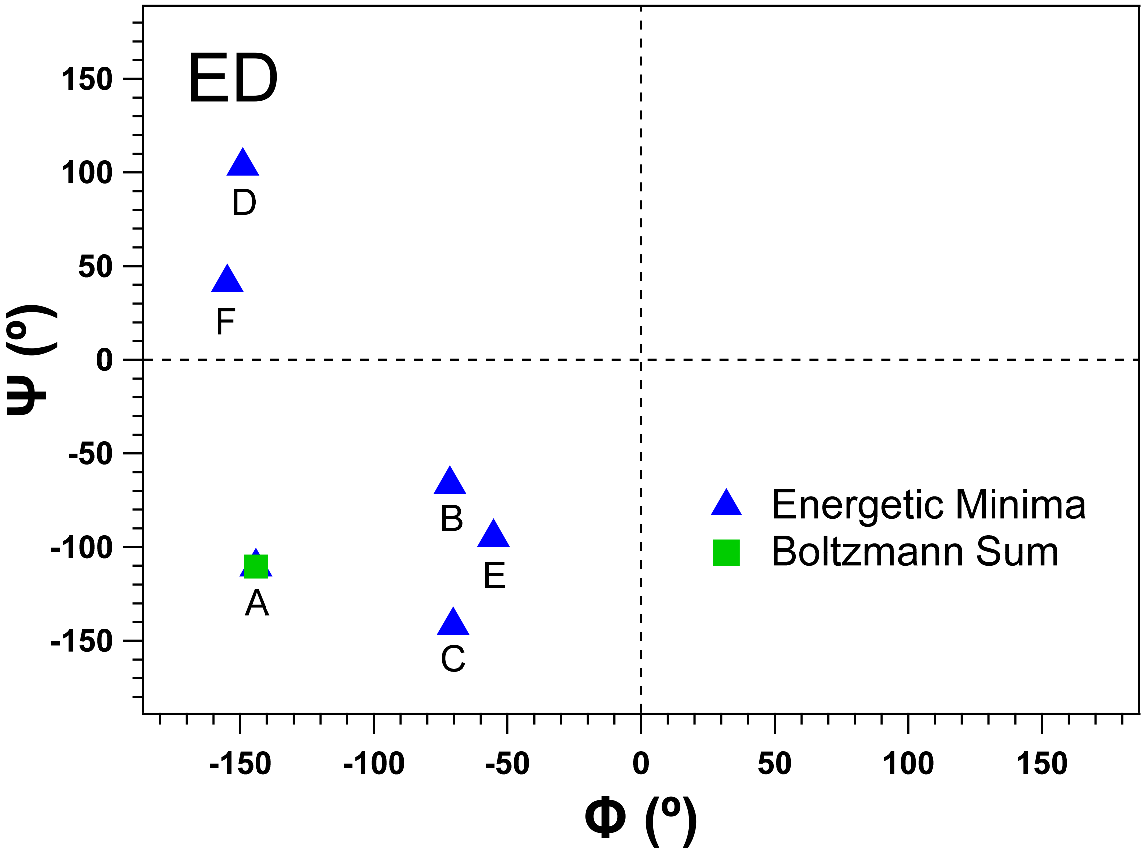

Figure S26: Ramachandran plot of the computed φ and ψ bond angles for each of the energetic minima of the EA dipeptide (Figure S3), presented in degrees (°). The Boltzmann sums of the weighted values are also presented.

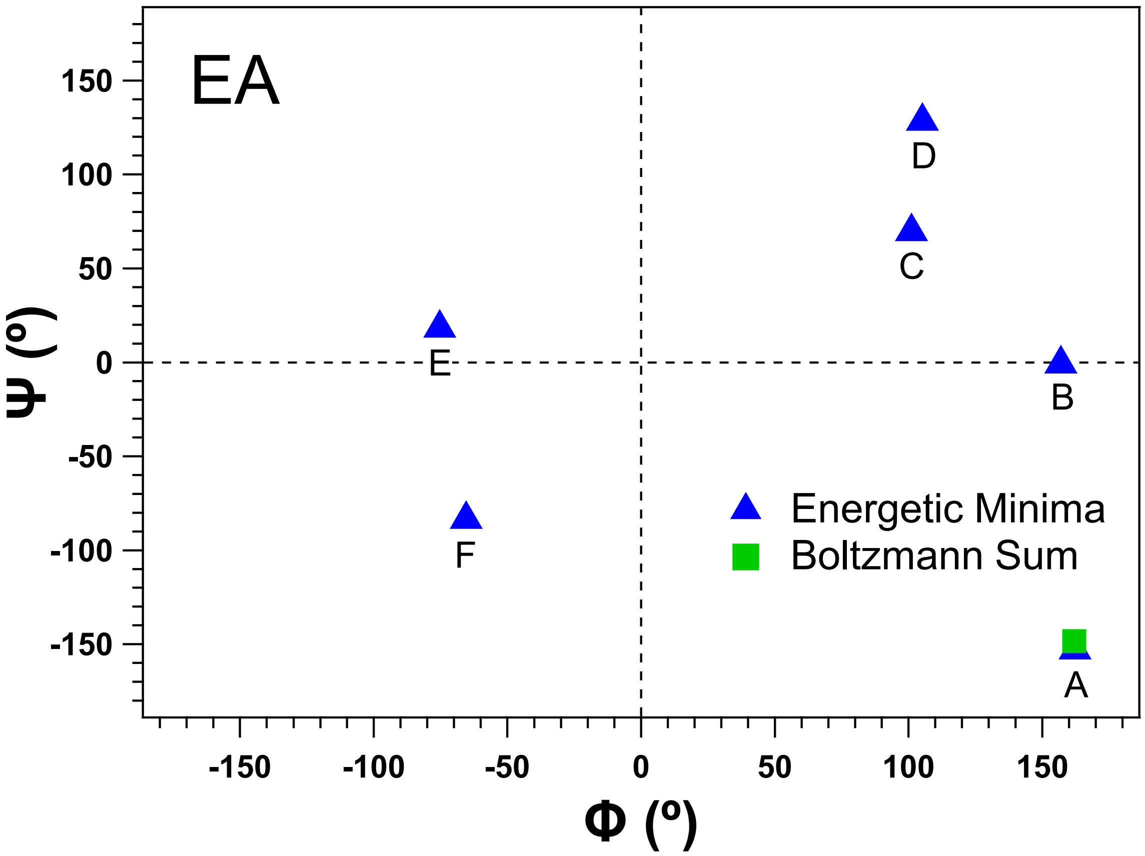

Figure S27: Ramachandran plot of the computed φ and ψ bond angles for each of the energetic minima of the EGE tripeptide (Figure S4), presented in degrees (^{O}). The Boltzmann sums of the weighted values are also presented.

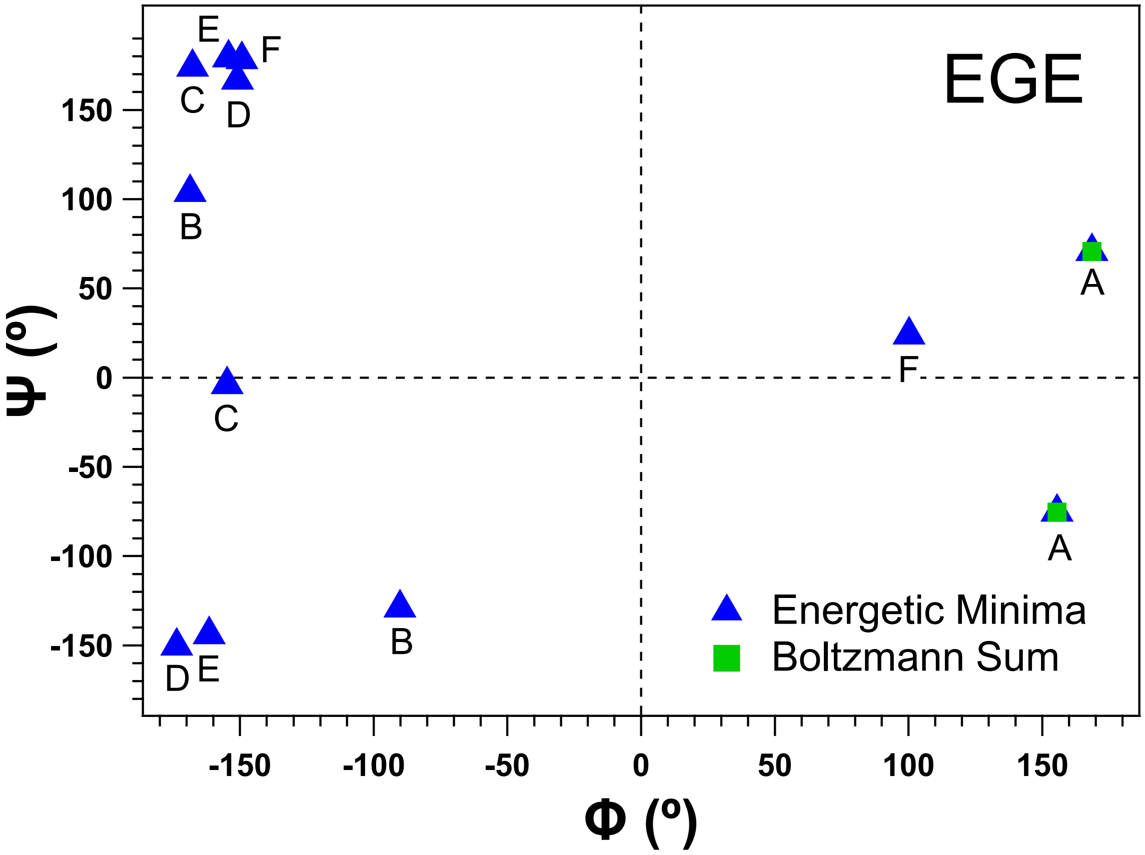

Figure S28: Ramachandran plot of the computed φ and ψ bond angles for each of the energetic minima of the EDE tripeptide (Figure S5), presented in degrees (^{O}). The Boltzmann sums of the weighted values are also presented.

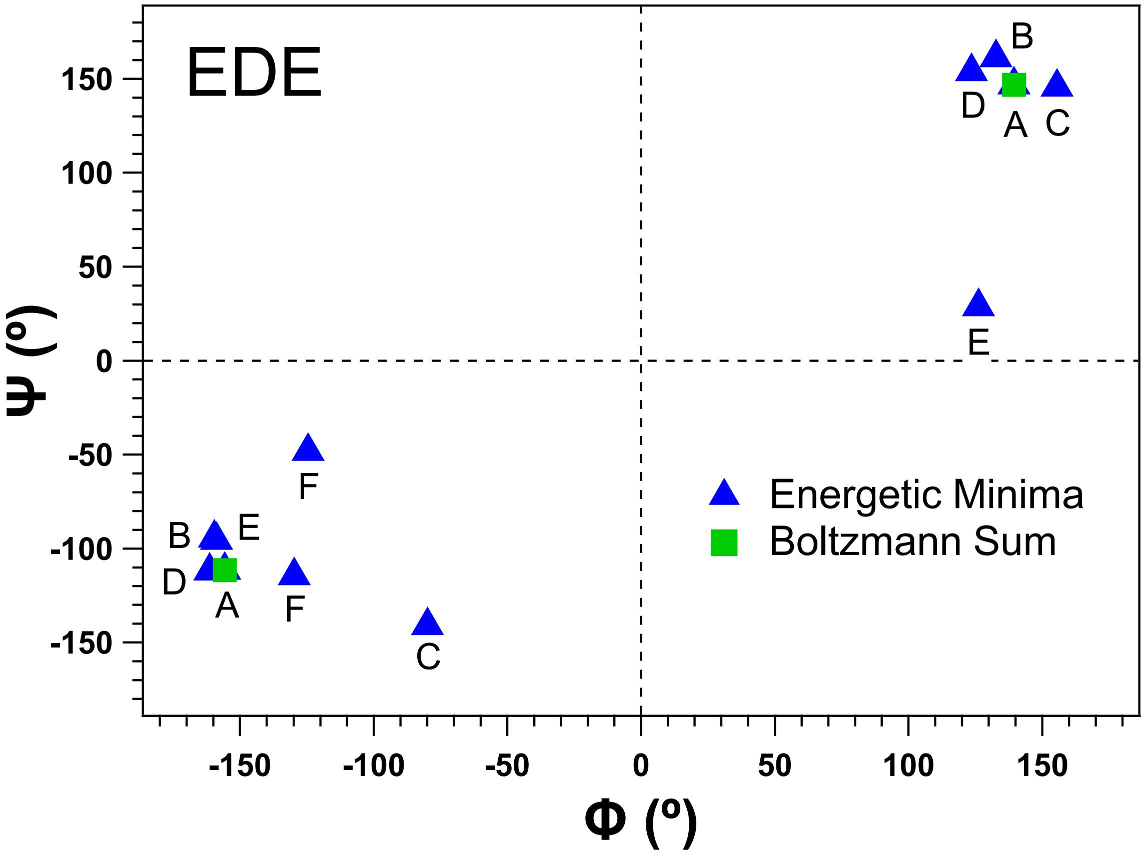

Figure S29: Ramachandran plot of the computed φ and ψ bond angles for each of the energetic minima of the DEA tripeptide (Figure S6), presented in degrees (°). The Boltzmann sums of the weighted values are also presented.

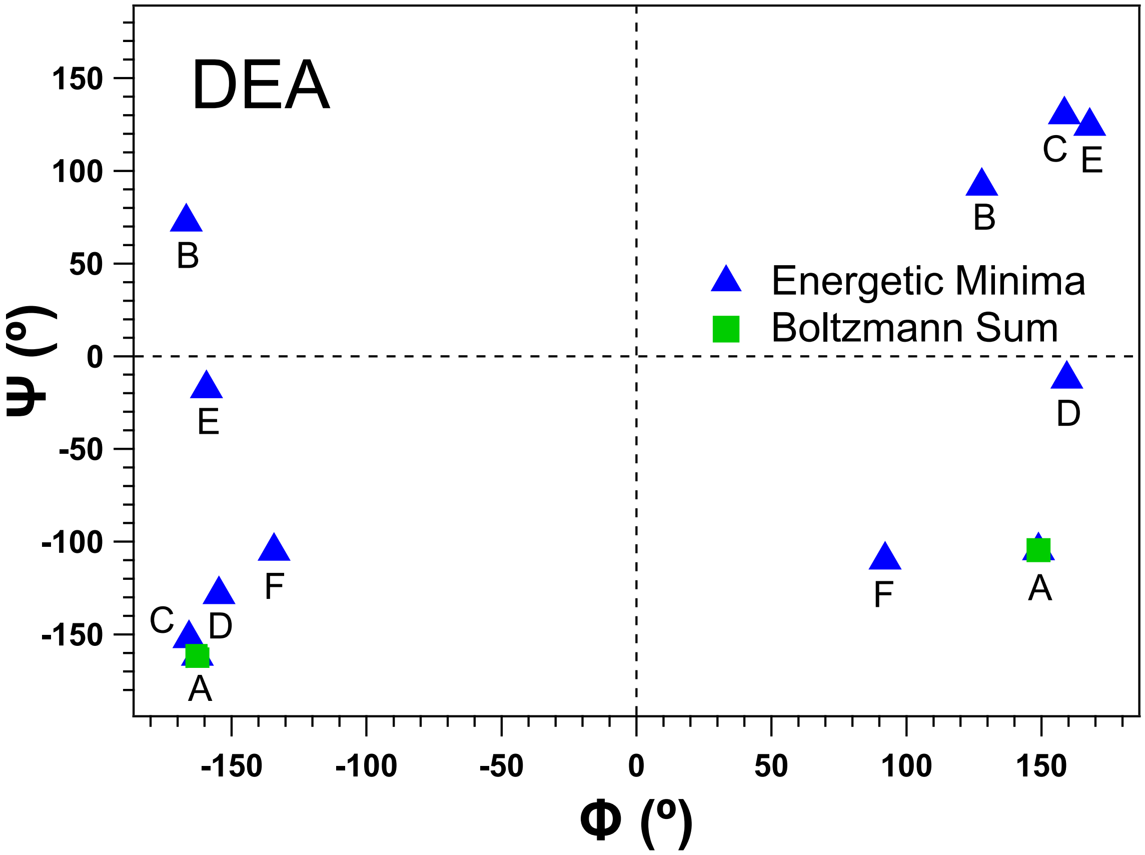

Figure S30: Ramachandran plot of the computed φ and ψ bond angles for each of the energetic minima of the EGED tetrapeptide (Figure S7), presented in degrees (^{O}). The Boltzmann sums of the weighted values are also presented.

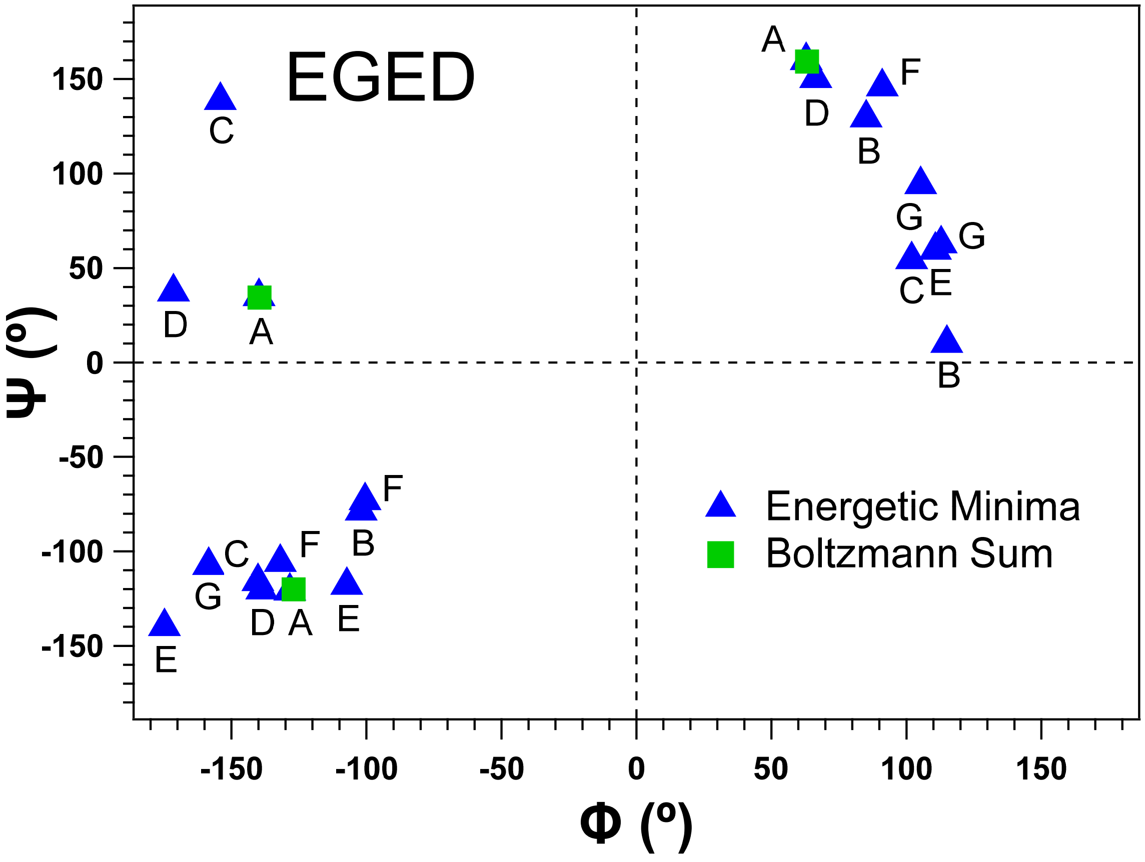

Figure S31: Ramachandran plot of the computed φ and ψ bond angles for each of the energetic minima of the EDEA tetrapeptide (Figure S8), presented in degrees (°). The Boltzmann sums of the weighted values are also presented.

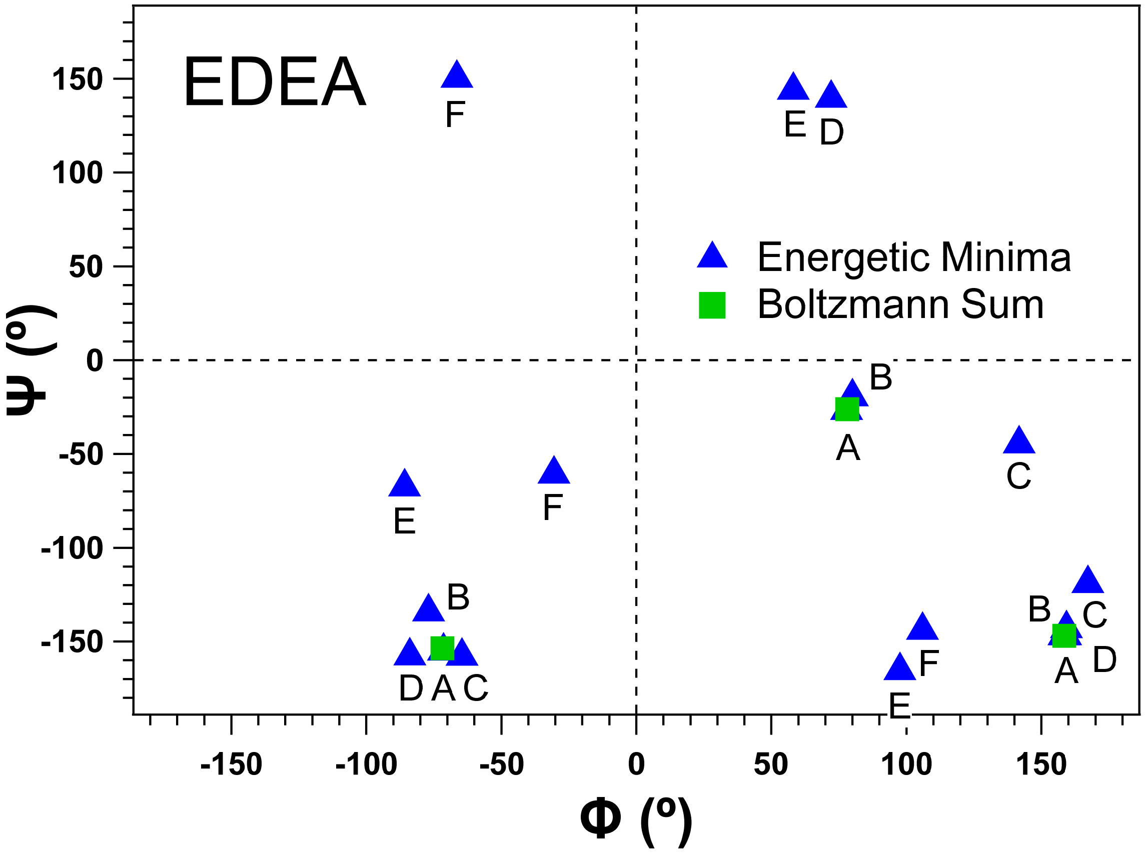

Figure S32: Ramachandran plot of the computed φ and ψ bond angles for each of the energetic minima of the EGEDE pentapeptide (Figure S9), presented in degrees (^{O}). The Boltzmann sums of the weighted values are also presented.

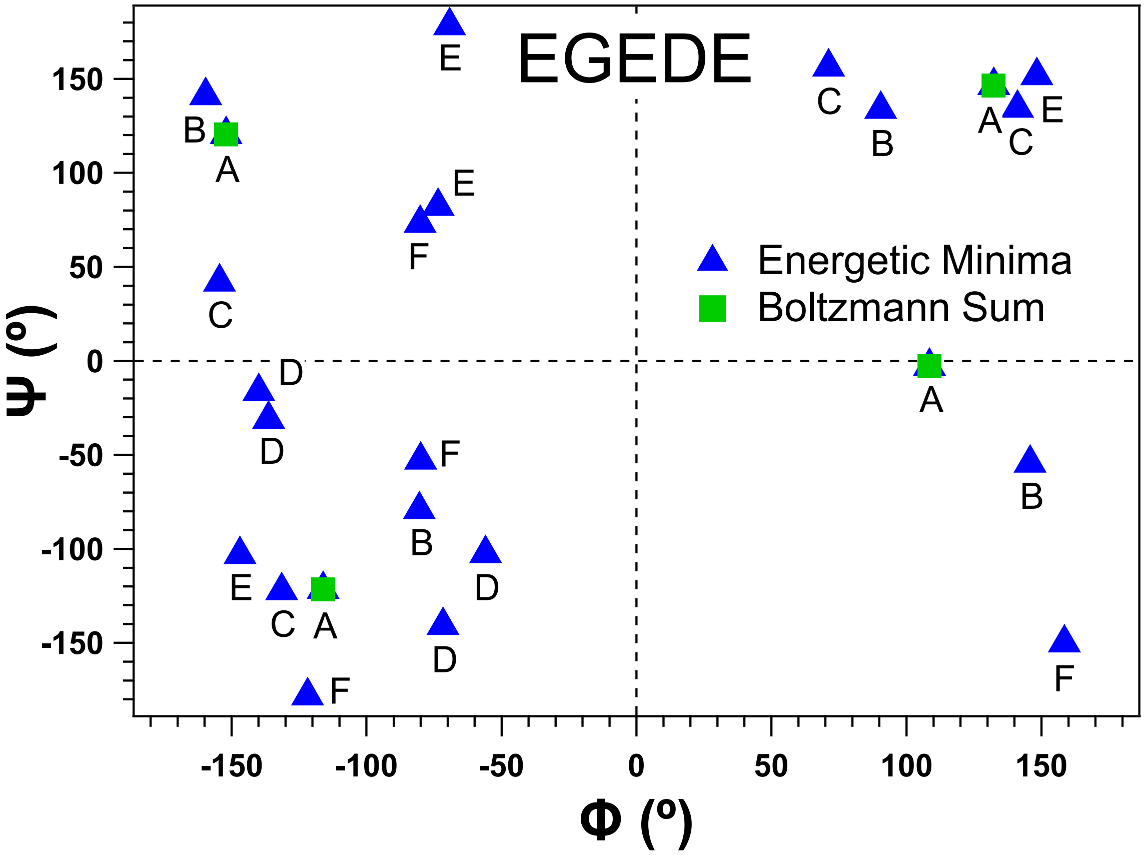

Figure S33: Ramachandran plot of the computed φ and ψ bond angles for each of the energetic minima of the GEDEA pentapeptide (Figure S10), presented in degrees (^{O}). The Boltzmann sums of the weighted values are also presented.

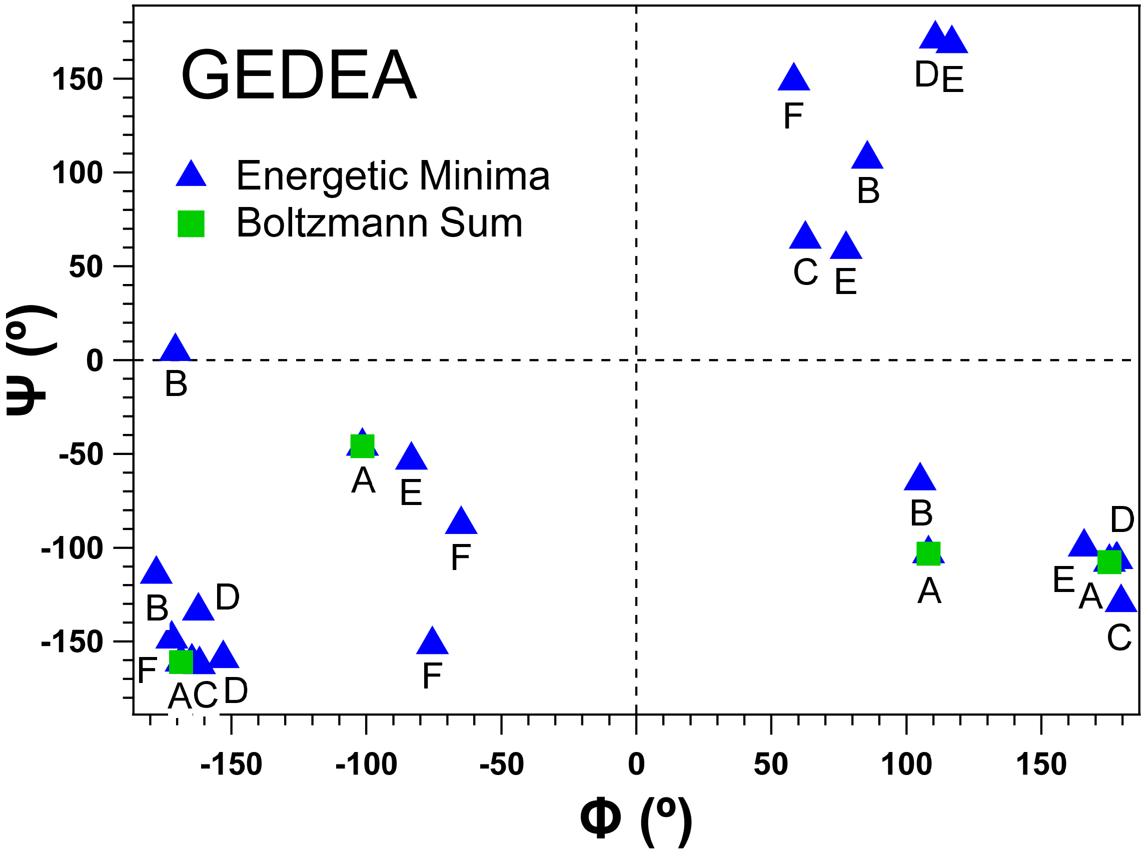

Table S12: Cartesian coordinates for the lowest energy molecular geometry of the **EG** dipeptide computed at the B3LYP method and 6-311+G(2*df*,2*pd*) level of theory.

**Standard Orientation**					
Center Number	Atomic Number	Atomic Type	Coordinates (Angstroms) X	Y	Z
1	7	0	-1.50704	-1.52097	0.824535
2	6	0	0.710256	-0.98551	-0.1146
3	7	0	1.784215	-0.22323	0.041741
4	6	0	4.050813	0.683902	0.081254
5	8	0	5.245218	0.593102	-0.23918
6	8	0	3.456714	1.570023	0.750347
7	8	0	0.675092	-2.07717	-0.69195
8	6	0	-3.86911	0.645715	-0.10249
9	8	0	-4.84815	1.264159	-0.45513
10	8	0	-3.95557	-0.62464	0.281078
11	1	0	1.787874	0.656831	0.565861
12	1	0	-1.29428	-1.85609	1.756963
13	1	0	-3.05243	-1.00746	0.538763
14	6	0	-2.49256	1.308979	-0.04349
15	1	0	-2.59586	2.233905	-0.60678
16	1	0	-2.3249	1.602985	0.998361
17	6	0	-1.2704	0.521816	-0.55749
18	1	0	-1.53486	-0.05963	-1.44435
19	1	0	-0.52015	1.244876	-0.87922
20	6	0	-0.60256	-0.41086	0.466161
21	1	0	-0.38242	0.16508	1.36708
22	6	0	3.135399	-0.48419	-0.42336
23	1	0	3.175793	-0.53531	-1.51219
24	1	0	3.506834	-1.43376	-0.03654
25	1	0	-1.26328	-2.28493	0.190872

EG computed electronic energy: -759.4111962 Hartrees

Table S13: Cartesian coordinates for the lowest energy molecular geometry of the **ED** dipeptide computed at the B3LYP method and 6-311+G(2*df*,2*pd*) level of theory.

Standard Orientation					
Center Number	Atomic Number	Atomic Type	Coordinates (Angstroms)		
			X	Y	Z
1	6	0	-0.19786	0.582797	1.284099
2	6	0	4.248087	0.141246	-0.54997
3	8	0	3.891639	-1.01087	-0.13222
4	8	0	5.402057	0.486209	-0.84381
5	8	0	0.141523	0.744635	2.466265
6	6	0	-1.79508	-2.22218	-0.26224
7	8	0	-0.61862	-2.11429	-0.74691
8	8	0	-2.26273	-3.2192	0.311715
9	6	0	-3.25331	1.46818	-0.61783
10	8	0	-3.69975	1.986406	0.435827
11	7	0	1.402552	-1.11325	0.55448
12	6	0	0.909252	0.266326	0.265058
13	1	0	0.511991	0.222833	-0.74374
14	6	0	2.037607	1.300188	0.343623
15	1	0	2.480037	1.268462	1.340602
16	1	0	1.561139	2.280342	0.257843
17	6	0	3.126738	1.197148	-0.74189
18	1	0	3.621401	2.163268	-0.84079
19	1	0	2.65631	0.992161	-1.71026
20	6	0	-2.11316	0.401238	-0.41793
21	1	0	-1.40095	0.502152	-1.23046
22	6	0	-2.73569	-1.00678	-0.44585
23	1	0	-3.2255	-1.11561	-1.41725
24	1	0	-3.51858	-1.07007	0.310945
25	7	0	-1.46154	0.675915	0.868082
26	1	0	-2.13258	1.042914	1.538121
27	1	0	2.435607	-1.19107	0.204526
28	8	0	-3.60868	1.6539	-1.79945
29	1	0	0.698067	-1.76519	0.096123
30	1	0	1.402771	-1.25209	1.562679

ED computed electronic energy: -986.7598636 Hartrees

Table S14: Cartesian coordinates for the lowest energy molecular geometry of the **EA** dipeptide computed at the B3LYP method and 6-311+G(2*df*,2*pd*) level of theory.

Standard Orientation					
Center Number	Atomic Number	Atomic Type	Coordinates (Angstroms)		
			X	Y	Z
1	6	0	0.504134	-0.92433	-0.11506
2	6	0	-4.10863	0.612088	-0.09329
3	8	0	-4.18316	-0.70093	0.105159
4	8	0	-5.08652	1.257812	-0.39694
5	8	0	0.523906	-1.93007	-0.83244
6	6	0	3.601081	1.121288	-0.00271
7	8	0	2.911148	1.913841	0.692605
8	7	0	-1.74181	-1.62695	0.628701
9	1	0	-3.28347	-1.1022	0.346215
10	1	0	-1.45117	-2.28919	-0.09343
11	7	0	1.548515	-0.16658	0.197257
12	1	0	1.468162	0.714798	0.715694
13	6	0	-0.84895	-0.46181	0.474193
14	1	0	-0.68509	-0.01449	1.456307
15	6	0	-1.48931	0.594273	-0.44173
16	1	0	-1.70011	0.138205	-1.41236
17	1	0	-0.74201	1.367468	-0.62343
18	6	0	-2.75076	1.283248	0.116347
19	1	0	-2.85019	2.274051	-0.3216
20	1	0	-2.635	1.436923	1.1948
21	6	0	2.9019	-0.25039	-0.34293
22	1	0	2.848747	-0.34173	-1.42962
23	6	0	3.686583	-1.43461	0.218904
24	1	0	3.218072	-2.38132	-0.05291
25	1	0	4.700498	-1.40375	-0.17636
26	1	0	3.742917	-1.37136	1.307922
27	8	0	4.748185	1.256347	-0.45451
28	1	0	-1.56313	-2.08727	1.51368

EA computed electronic energy: -798.7399453 Hartrees

Table S15: Cartesian coordinates for the lowest energy molecular geometry of the **EGE** tripeptide computed at the B3LYP method and 6-311+G(2*df*,2*pd*) level of theory.

Standard Orientation					
Center Number	Atomic Number	Atomic Type	Coordinates (Angstroms)		
			X	Y	Z
1	7	0	-2.16378	-0.43475	1.232108
2	6	0	-1.41429	1.860448	0.543477
3	7	0	-0.28877	1.595623	-0.13035
4	6	0	2.091064	1.772178	-0.67786
5	8	0	2.470311	2.094607	-1.80362
6	8	0	-1.58724	2.873662	1.226066
7	6	0	-5.03145	-0.92608	-0.00654
8	8	0	-4.63341	-1.21193	1.176452
9	8	0	-6.20387	-0.7572	-0.36753
10	1	0	-0.18826	0.714306	-0.66122
11	1	0	-1.87085	-0.18118	2.171371
12	6	0	-3.90402	-0.80648	-1.06243
13	1	0	-3.27782	-1.69857	-0.98768
14	1	0	-4.35447	-0.80724	-2.0543
15	6	0	-2.99142	0.436746	-0.95218
16	1	0	-2.12249	0.2687	-1.58738
17	1	0	-3.51635	1.313642	-1.33867
18	6	0	-2.53029	0.799461	0.467707
19	1	0	-3.36581	1.238107	1.008598
20	6	0	0.878698	2.466326	-0.04133
21	1	0	1.062378	2.712569	1.007312
22	1	0	0.706293	3.398346	-0.57967
23	1	0	-1.35244	-1.03145	0.812387
24	6	0	4.429206	-0.63958	0.926875
25	6	0	0.623302	-1.55824	-0.46265
26	8	0	0.230346	-0.81759	-1.39708
27	8	0	-0.08479	-1.88058	0.540606
28	8	0	3.932161	-0.30955	2.031997
29	8	0	5.446137	-1.32603	0.695391
30	6	0	3.10883	-1.27927	-1.20363
31	1	0	3.964114	-1.90551	-1.46306
32	1	0	2.692717	-0.86977	-2.12555
33	6	0	2.040905	-2.14504	-0.5195
34	1	0	2.341169	-2.4154	0.492566
35	1	0	1.946311	-3.08491	-1.07563
36	1	0	-3.11487	-0.9656	1.298244
37	7	0	2.629867	0.800916	0.069525
38	1	0	2.417807	0.701514	1.057302
39	6	0	3.672667	-0.1241	-0.35034
40	1	0	4.394949	0.41858	-0.96135

EGE computed electronic energy: -1234.1921064 Hartrees

Table S16: Cartesian coordinates for the lowest energy molecular geometry of the **EDE** tripeptide computed at the B3LYP method and 6-311+G(2*df*,2*pd*) level of theory.

Standard Orientation					
Center Number	Atomic Number	Atomic Type	Coordinates (Angstroms)		
			X	Y	Z
1	6	0	2.42797	-0.05598	1.222741
2	6	0	6.430282	0.889471	-0.61327
3	8	0	6.105899	-0.08418	-1.37563
4	8	0	7.546262	1.428099	-0.55523
5	8	0	3.163494	-0.06131	2.214271
6	6	0	1.237269	-2.61036	-0.46684
7	8	0	1.786543	-3.45906	0.258642
8	8	0	1.707219	-2.14672	-1.55802
9	6	0	-1.25952	0.069141	0.95146
10	8	0	-1.40199	-0.02338	2.176152
11	7	0	3.866934	-1.04897	-0.51024
12	6	0	3.056783	0.163147	-0.16112
13	1	0	2.281416	0.214085	-0.91755
14	6	0	3.897988	1.455525	-0.23151
15	1	0	3.323019	2.230005	0.280407
16	1	0	3.947058	1.753138	-1.28132
17	6	0	5.329644	1.427572	0.337745
18	1	0	5.347434	0.839258	1.257431
19	1	0	5.6227	2.439062	0.61815
20	6	0	0.052345	-0.47233	0.320484
21	1	0	0.273199	0.062698	-0.59976
22	6	0	-0.08731	-1.98247	0.013112
23	1	0	-0.84566	-2.09201	-0.764
24	1	0	-0.42908	-2.49669	0.910096
25	7	0	1.097357	-0.2121	1.317576
26	1	0	0.737436	-0.30238	2.262758
27	1	0	4.799103	-0.7283	-0.98744
28	1	0	3.228658	-1.67494	-1.08013
29	1	0	4.111074	-1.55487	0.337773
30	6	0	-3.47181	2.527562	-0.55481
31	6	0	-6.46213	-1.03812	-0.46004
32	8	0	-7.00993	-1.04463	0.670342
33	8	0	-6.85605	-1.62762	-1.50136
34	8	0	-2.72079	2.461535	-1.56518
35	8	0	-4.28702	3.421159	-0.25153
36	6	0	-3.34698	1.345489	0.476147
37	1	0	-3.14801	1.795422	1.450224
38	6	0	-4.62655	0.493861	0.622261
39	1	0	-5.40194	1.162445	0.996215
40	1	0	-4.45803	-0.24443	1.409266
41	6	0	-5.14291	-0.21199	-0.6314
42	1	0	-4.38899	-0.89797	-1.02815
43	1	0	-5.32332	0.515539	-1.42706
44	7	0	-2.1477	0.580171	0.101395
45	1	0	-1.87161	0.831827	-0.84752

EDE computed electronic energy: -1461.4753134 Hartrees

Table S17: Cartesian coordinates for the lowest energy molecular geometry of the **DEA** tripeptide computed at the B3LYP method and 6-311+G(2*df*,2*pd*) level of theory.

Standard Orientation					
Center Number	Atomic Number	Atomic Type	Coordinates (Angstroms)		
			X	Y	Z
1	6	0	-1.79799	0.374823	-0.22595
2	6	0	1.404949	-2.1252	-0.97336
3	8	0	1.841771	-1.37095	-1.87397
4	8	0	2.089104	-2.71893	-0.09413
5	8	0	-1.44423	1.022837	-1.20937
6	6	0	4.633507	1.593796	-0.5993
7	8	0	5.538172	0.737836	-0.11623
8	8	0	4.967737	2.59703	-1.19233
9	6	0	1.472322	0.328847	1.436997
10	8	0	1.239205	0.147793	2.632804
11	7	0	0.544357	0.256468	0.468223
12	6	0	-0.76911	-0.33327	0.678535
13	1	0	-1.03695	-0.15302	1.718769
14	6	0	-0.73081	-1.86799	0.452903
15	1	0	-1.75248	-2.24139	0.554634
16	1	0	-0.13862	-2.3081	1.256729
17	6	0	-0.13228	-2.32038	-0.88895
18	1	0	-0.60719	-1.8036	-1.72331
19	1	0	-0.32943	-3.39066	-0.99784
20	6	0	2.932194	0.592692	1.019615
21	1	0	3.31371	1.272715	1.785171
22	6	0	3.177349	1.209478	-0.36629
23	1	0	2.574892	2.102975	-0.515
24	1	0	2.901506	0.4762	-1.13586
25	7	0	3.711122	-0.66301	1.127124
26	1	0	0.822089	0.350097	-0.4992
27	7	0	-3.07687	0.196068	0.143157
28	6	0	-5.45897	0.6896	0.488656
29	8	0	-5.25381	0.050074	1.550715
30	8	0	-6.50118	1.255527	0.101
31	1	0	-3.33517	-0.22249	1.033162
32	6	0	-4.2421	0.787739	-0.50207
33	1	0	-4.04666	1.847004	-0.68349
34	6	0	-4.5661	0.118627	-1.84013
35	1	0	-3.73178	0.220393	-2.5348
36	1	0	-4.76949	-0.94472	-1.69258
37	1	0	-5.45562	0.582739	-2.26453
38	1	0	3.259414	-1.41586	0.576115
39	1	0	3.693793	-0.97205	2.092147
40	1	0	5.03575	0.010726	0.388511

DEA computed electronic energy: -1234.2003724 Hartrees

Table S18: Cartesian coordinates for the lowest energy molecular geometry of the **EGED** tetrapeptide computed at the B3LYP method and 6-311+G(2*df*,2*pd*) level of theory.

Standard Orientation					
Center Number	Atomic Number	Atomic Type	Coordinates (Angstroms)		
			X	Y	Z
1	7	0	3.292105	-0.89883	0.682917
2	6	0	2.178859	1.234195	0.71969
3	7	0	1.268552	1.725826	-0.15631
4	6	0	-1.0702	1.454805	-0.6816
5	8	0	-0.67774	0.959007	-1.74059
6	8	0	2.10015	1.375877	1.93412
7	6	0	7.216014	1.371151	-0.32578
8	8	0	8.276111	0.703675	-0.24411
9	8	0	7.0836	2.611045	-0.443
10	1	0	1.281345	1.361749	-1.10053
11	1	0	3.170812	-0.79328	1.686559
12	1	0	2.158497	-2.42612	0.050556
13	6	0	5.893851	0.524729	-0.3284
14	1	0	5.678935	0.30143	-1.38112
15	1	0	6.119335	-0.42966	0.152431
16	6	0	4.679881	1.207837	0.302818
17	1	0	4.835433	1.341263	1.376612
18	1	0	4.600327	2.210405	-0.12231
19	6	0	3.347849	0.460701	0.09449
20	1	0	3.176267	0.354787	-0.98031
21	6	0	-0.05975	2.162	0.236913
22	1	0	-0.17747	3.244905	0.135404
23	1	0	-0.22987	1.911169	1.281593
24	6	0	-3.03152	-0.84662	-1.15747
25	6	0	-4.83236	3.376329	0.553832
26	8	0	-3.79364	3.115636	1.22666
27	8	0	-5.54681	4.396146	0.634423
28	8	0	-3.35029	-1.42885	-2.19116
29	6	0	0.656787	-3.60117	-0.4699
30	8	0	0.330406	-4.67865	-0.90039
31	8	0	1.974357	-3.33377	-0.2855
32	6	0	-2.16598	-3.41285	1.314723
33	8	0	-1.75255	-2.79001	2.324028
34	7	0	-2.33349	1.383575	-0.24371
35	6	0	-3.34316	0.668681	-1.01702
36	1	0	-3.33776	1.024311	-2.05002
37	6	0	-4.73603	0.896879	-0.4115
38	1	0	-4.72679	0.578152	0.633356
39	1	0	-5.4103	0.221058	-0.94245
40	6	0	-5.28518	2.329143	-0.5094
41	1	0	-6.37604	2.302035	-0.46038
42	1	0	-5.04708	2.752133	-1.49184
43	6	0	-1.77892	-2.7303	-0.05326
44	1	0	-2.13582	-3.32248	-0.88825
45	6	0	-0.26859	-2.44724	-0.15759
46	1	0	0.046775	-1.99019	0.78344

47	1	0	-0.09811	-1.70536	-0.9451
48	7	0	-2.44079	-1.42	-0.09467
49	1	0	-2.26349	-0.84704	0.717118
50	1	0	-2.71142	2.02138	0.490296
51	8	0	-2.85044	-4.45258	1.252061
52	1	0	4.187205	-1.35452	0.541485

EGED computed electronic energy: -1669.5877995 Hartrees

Table S19: Cartesian coordinates for the lowest energy molecular geometry of the **EDEA** tetrapeptide computed at the B3LYP method and 6-311+G(2*df*,2*pd*) level of theory.

Standard Orientation					
Center Number	Atomic Number	Atomic Type	Coordinates (Angstroms)		
			X	Y	Z
1	6	0	2.226392	0.244428	0.785553
2	8	0	1.926505	-0.18575	1.902997
3	6	0	1.506945	4.090526	-0.44877
4	8	0	1.518653	5.298601	-0.14429
5	8	0	2.401917	3.447978	-1.07844
6	6	0	-0.80791	1.186938	0.903952
7	8	0	-1.62152	1.507649	1.766105
8	7	0	3.543084	-1.77982	0.558523
9	7	0	-1.01189	0.185477	0.023172
10	1	0	-0.27044	-0.09282	-0.61283
11	6	0	3.401298	-0.39998	0.020901
12	1	0	3.127173	-0.48268	-1.02876
13	6	0	4.699351	0.404868	0.184687
14	1	0	4.970619	0.427107	1.244498
15	1	0	4.464149	1.433439	-0.0964
16	6	0	0.505667	1.987781	0.762509
17	1	0	0.774274	2.262549	1.784438
18	6	0	0.240296	3.266445	-0.06597
19	1	0	-0.45132	3.891894	0.491551
20	1	0	-0.24841	2.975014	-0.99973
21	7	0	1.594675	1.236289	0.153395
22	1	0	2.061734	1.773594	-0.59734
23	1	0	2.853294	-2.44938	0.107794
24	1	0	4.574085	-2.07863	0.49645
25	6	0	-3.45165	0.006505	-0.51724
26	6	0	0.609106	-2.59329	-0.65645
27	8	0	0.809458	-1.62159	-1.41458
28	8	0	1.49898	-3.37935	-0.20861
29	8	0	-3.3955	0.745364	-1.49546
30	6	0	-6.97287	-1.06312	0.191444
31	8	0	-8.12735	-0.98802	-0.28687
32	7	0	-4.60436	-0.35941	0.079494
33	1	0	-4.62598	-1.03418	0.834914
34	6	0	-2.18464	-0.6681	0.070212
35	1	0	-2.38785	-0.90639	1.118033
36	6	0	-1.94114	-1.9733	-0.71544
37	1	0	-1.72963	-1.71353	-1.75404
38	1	0	-2.88601	-2.52096	-0.70728
39	6	0	-0.83092	-2.88807	-0.18412
40	1	0	-1.04079	-3.92234	-0.47225
41	1	0	-0.81509	-2.88894	0.909015
42	6	0	-6.34334	1.394434	-0.10471
43	1	0	-5.63937	2.092748	-0.55782
44	1	0	-7.34646	1.578776	-0.48964
45	1	0	-6.34792	1.560834	0.974943
46	8	0	-6.54608	-1.82638	1.091993

47	6	0	6.754127	-1.25203	-0.13092
48	8	0	6.132474	-2.19648	0.454756
49	8	0	7.977449	-1.2027	-0.34679
50	6	0	5.904301	-0.06274	-0.65603
51	1	0	6.587278	0.775614	-0.79165
52	1	0	5.558454	-0.33483	-1.65964
53	1	0	3.252974	-1.72147	1.538896
54	6	0	-5.93833	-0.05087	-0.41645
55	1	0	-5.94533	-0.1805	-1.50085

EDEA computed electronic energy: -1708.9470997 Hartrees

Table S20: Cartesian coordinates for the lowest energy molecular geometry of the **EGEDE** pentapeptide computed at the B3LYP method and 6-311+G(2*df*,2*pd*) level of theory.

Standard Orientation					
Center Number	Atomic Number	Atomic Type	Coordinates (Angstroms)		
			X	Y	Z
1	7	0	4.670552	-1.02122	-0.5056
2	6	0	4.281051	1.338627	0.292677
3	7	0	3.059588	0.879638	0.647076
4	6	0	0.861462	1.9783	0.2073
5	8	0	1.056789	2.090265	-0.99376
6	8	0	4.651888	2.492682	0.481452
7	6	0	9.153152	-0.38851	0.007008
8	8	0	10.09721	-0.4291	-0.82159
9	8	0	9.212646	-0.45954	1.255746
10	1	0	2.789933	-0.0761	0.44777
11	1	0	4.589175	-1.43047	0.421741
12	6	0	7.723507	-0.28414	-0.62711
13	1	0	7.817256	0.237201	-1.58321
14	1	0	7.445266	-1.31817	-0.87032
15	6	0	6.645227	0.375506	0.234959
16	1	0	6.620094	-0.10848	1.214684
17	1	0	6.906506	1.417975	0.413588
18	6	0	5.2369	0.343371	-0.39555
19	1	0	5.31275	0.723729	-1.4189
20	6	0	2.026086	1.730996	1.189473
21	1	0	2.479175	2.697504	1.409472
22	1	0	1.649492	1.304187	2.121604
23	6	0	-2.62428	1.401137	0.669119
24	6	0	-3.41143	5.984087	-0.29792
25	8	0	-2.8436	6.575624	0.651503
26	8	0	-4.18941	6.471466	-1.15604
27	8	0	-2.80695	1.319918	1.881394
28	6	0	-6.62556	-1.24977	-0.11125
29	8	0	-6.46196	-1.77504	1.011089
30	8	0	-7.63793	-1.28827	-0.85582
31	6	0	-3.28431	-1.73513	-0.61268
32	8	0	-2.92495	-1.66174	-1.79435
33	7	0	-0.3421	2.107556	0.806946
34	6	0	-1.60392	2.430996	0.127459
35	1	0	-1.42799	2.283346	-0.93708
36	6	0	-1.99702	3.898247	0.397954
37	1	0	-1.10741	4.504397	0.218098
38	1	0	-2.23086	4.00862	1.457412
39	6	0	-3.14414	4.445922	-0.45016
40	1	0	-4.08121	3.933529	-0.21412
41	1	0	-2.96076	4.255153	-1.51157
42	6	0	-4.07737	-0.55426	-0.00621
43	1	0	-4.19668	-0.66317	1.067224
44	6	0	-5.45416	-0.36762	-0.65968
45	1	0	-5.37833	-0.5051	-1.73883
46	1	0	-5.75865	0.669462	-0.49586

47	7	0	-3.21563	0.620276	-0.2551
48	1	0	-2.91254	0.711677	-1.21216
49	1	0	-0.43651	1.897557	1.788462
50	1	0	5.347775	-1.59729	-0.99437
51	6	0	-2.46714	-5.14482	0.694154
52	6	0	1.356993	-2.10227	-0.61053
53	8	0	2.242597	-2.03212	-1.61576
54	8	0	1.645527	-1.72393	0.512455
55	8	0	-2.08134	-6.23696	0.212772
56	8	0	-3.04295	-4.92001	1.779753
57	7	0	-2.93768	-2.74067	0.206337
58	1	0	-3.38781	-2.85024	1.108667
59	6	0	-2.11212	-3.88638	-0.17904
60	1	0	-2.31071	-4.12466	-1.22301
61	6	0	-0.6088	-3.596	0.015064
62	1	0	-0.08563	-4.55311	-0.03073
63	1	0	-0.46682	-3.19182	1.017835
64	6	0	0.007325	-2.63214	-1.00951
65	1	0	-0.63505	-1.75696	-1.13099
66	1	0	0.074484	-3.09109	-1.99518
67	1	0	3.072554	-1.59986	-1.27176

EGEDE computed electronic energy: -2144.2804542 Hartrees

Table S21: Cartesian coordinates for the lowest energy molecular geometry of the **GEDEA** pentapeptide computed at the B3LYP method and 6-311+G(2*df*,2*pd*) level of theory.

**Standard Orientation**					
Center Number	Atomic Number	Atomic Type	Coordinates (Angstroms)		
			X	Y	Z
1	6	0	2.194579	0.899559	-1.17193
2	8	0	2.589605	1.696269	-2.02392
3	6	0	1.654069	1.027619	2.885428
4	8	0	1.525392	1.518829	4.016166
5	8	0	2.726043	0.520002	2.415683
6	6	0	-0.88771	-0.06308	-0.0344
7	8	0	-0.87562	-0.92623	-0.90881
8	7	0	3.211788	-0.89971	0.2601
9	7	0	-2.00781	0.569835	0.372716
10	1	0	-1.92718	1.325307	1.039724
11	6	0	2.898236	-0.4842	-1.101
12	1	0	2.210558	-1.20805	-1.54895
13	6	0	4.195347	-0.48249	-1.93839
14	1	0	3.965738	-0.16181	-2.95105
15	1	0	4.538676	-1.51526	-1.97636
16	6	0	0.459146	0.356816	0.619929
17	1	0	0.989697	-0.58322	0.712445
18	6	0	0.372996	1.02218	2.000678
19	1	0	-0.38993	0.512269	2.592712
20	1	0	0.03727	2.05733	1.905532
21	7	0	1.173716	1.200033	-0.34548
22	1	0	0.732044	2.108313	-0.53843
23	6	0	-4.31295	-0.38305	0.439169
24	6	0	-1.75343	3.388641	-0.34916
25	8	0	-2.09641	3.623887	0.83407
26	8	0	-0.6189	3.546912	-0.86459
27	8	0	-4.23411	-0.56458	1.65079
28	6	0	-7.26095	-2.20173	-1.05363
29	8	0	-8.18366	-2.99148	-0.75119
30	7	0	-5.33002	-0.8447	-0.31888
31	1	0	-5.32999	-0.77125	-1.33006
32	6	0	-3.27397	0.467532	-0.33235
33	1	0	-3.05775	-0.02814	-1.2796
34	6	0	-3.85147	1.876465	-0.60488
35	1	0	-4.15749	2.324515	0.342653
36	1	0	-4.74977	1.748751	-1.21306
37	6	0	-2.86588	2.829382	-1.28822
38	1	0	-3.42193	3.697556	-1.66049
39	1	0	-2.39374	2.359316	-2.15303
40	6	0	-7.39233	-0.77977	1.057401
41	1	0	-7.79038	0.068124	0.494103
42	1	0	-8.22735	-1.39885	1.384897
43	1	0	-6.85122	-0.3994	1.923784
44	8	0	-6.90504	-1.8097	-2.19122
45	6	0	6.692148	-0.12645	-1.54102
46	8	0	7.418477	0.156564	-2.46858

47	8	0	7.138523	-1.00019	-0.61238
48	6	0	5.301249	0.433144	-1.34951
49	1	0	5.122581	0.572788	-0.28243
50	1	0	5.255621	1.407458	-1.82653
51	1	0	3.024604	-0.24267	1.068877
52	6	0	3.731801	-2.0975	0.540689
53	8	0	3.860575	-3.04574	-0.24387
54	6	0	4.353261	-2.19978	1.929669
55	1	0	3.986659	-3.1062	2.422376
56	1	0	4.107708	-1.3304	2.536154
57	7	0	5.816293	-2.23698	1.686687
58	1	0	6.326569	-2.18045	2.559226
59	1	0	6.05052	-3.11757	1.241252
60	6	0	-6.46555	-1.61682	0.167919
61	1	0	-6.09673	-2.46031	0.756242
62	1	0	6.506816	-1.19283	0.118437

GEDEA computed electronic energy: -1917.0039035 Hartrees

Table S22: Cartesian coordinates for the lowest energy molecular geometry of the **EGEDEA** hexapeptide computed at the B3LYP method and 6-311+G(2*df*,2*pd*) level of theory.

Standard Orientation					
Center Number	Atomic Number	Atomic Type	Coordinates (Angstroms)		
			X	Y	Z
1	6	0	4.448842	1.51965	0.276874
2	7	0	3.181407	1.847858	-0.06507
3	6	0	1.079522	3.146036	0.117765
4	7	0	-0.06949	2.788785	0.723738
5	6	0	-2.27172	1.917974	0.419084
6	7	0	-3.04793	1.639953	-0.62803
7	7	0	-2.46849	-1.36395	-0.23097
8	6	0	-1.97371	-3.5734	0.725679
9	7	0	-2.00357	-4.88656	0.42811
10	7	0	5.366478	-0.71791	0.112946
11	6	0	-2.59346	-7.27501	0.647986
12	6	0	-5.05147	1.668301	-2.83241
13	8	0	5.004758	1.963264	1.279787
14	8	0	1.161717	3.846509	-0.88275
15	8	0	-2.17668	1.193335	1.422725
16	8	0	-6.01724	1.592819	-3.61609
17	8	0	-3.99954	2.355103	-2.97091
18	8	0	-2.02972	-3.13113	1.871885
19	8	0	-2.58071	-7.2269	-0.6057
20	8	0	-2.88898	-8.23558	1.393024
21	1	0	5.694751	-0.44865	1.036353
22	1	0	6.167905	-1.20414	-0.31303
23	1	0	2.729792	1.288886	-0.77607
24	1	0	-0.04159	2.147869	1.501657
25	1	0	-3.12811	2.206428	-1.49661
26	1	0	-2.34577	-0.90784	0.6646
27	1	0	-2.10059	-5.22445	-0.52399
28	6	0	-0.9001	-6.19297	2.224433
29	1	0	-0.63175	-5.29276	2.777766
30	1	0	-1.07099	-7.01351	2.920739
31	1	0	-0.06676	-6.46198	1.571164
32	6	0	5.14262	0.524101	-0.65976
33	1	0	4.443471	0.278022	-1.45911
34	6	0	6.415213	1.108478	-1.32305
35	1	0	6.171829	2.126348	-1.64472
36	1	0	6.590698	0.527439	-2.23083
37	6	0	2.339704	2.62813	0.824812
38	1	0	2.084576	2.044675	1.71458
39	1	0	2.907278	3.494255	1.164547
40	6	0	-1.42693	3.203113	0.351547
41	1	0	-1.39054	3.588531	-0.66544
42	6	0	-1.90761	4.297548	1.333965
43	1	0	-1.15914	5.090118	1.331123
44	1	0	-1.89554	3.866072	2.338604
45	6	0	-3.98253	0.515666	-0.65735
46	1	0	-4.28708	0.329575	0.375408

47	6	0	-5.22312	0.879692	-1.49215
48	1	0	-5.86508	1.509297	-0.86882
49	1	0	-5.78033	-0.02706	-1.71601
50	6	0	-1.80308	-2.62986	-0.48228
51	1	0	-2.3044	-3.06067	-1.34799
52	6	0	-2.16975	-5.96249	1.399218
53	1	0	-2.97958	-5.69571	2.08166
54	6	0	1.963368	-1.51313	-0.22142
55	8	0	2.957795	-1.90587	0.565202
56	8	0	2.12394	-0.82218	-1.21321
57	6	0	-0.31307	-2.45437	-0.86727
58	1	0	-0.27175	-1.74811	-1.69597
59	1	0	0.060795	-3.41204	-1.23894
60	6	0	0.597122	-1.96032	0.261888
61	1	0	0.712747	-2.70823	1.042917
62	1	0	0.145936	-1.08158	0.73328
63	6	0	8.600805	-0.16501	-0.60343
64	8	0	8.018488	-1.24121	-0.9121
65	8	0	9.817006	-0.0053	-0.35042
66	6	0	7.718959	1.118821	-0.50788
67	1	0	7.507394	1.303786	0.547652
68	1	0	8.343949	1.953413	-0.83128
69	6	0	-3.58863	6.178788	1.960441
70	8	0	-4.80874	6.433388	2.113307
71	8	0	-2.60727	6.818847	2.41222
72	6	0	-3.27146	4.929247	1.064289
73	1	0	-4.0811	4.209324	1.188955
74	1	0	-3.3259	5.269289	0.024286
75	6	0	-3.30753	-0.79526	-1.14239
76	8	0	-3.54196	-1.29621	-2.23234
77	1	0	3.861163	-1.4974	0.299762

EGEDEA computed electronic energy: -2391.7359474 Hartrees
